# Supplementary figures and images for: Lower termite (Coptotermes heimi) gut fibrolytic bacterial consortium: Isolation, phylogenetic characterization, fibre degradation potential and in vitro digestibility
Source: PLoS One. 2025 Mar 10;20(3):e0318090. doi: 10.1371/journal.pone.0318090 (PMC11892857; doi:10.1371/journal.pone.0318090)

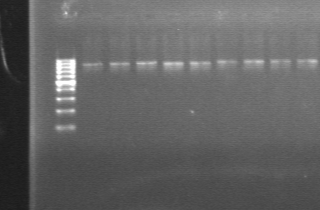


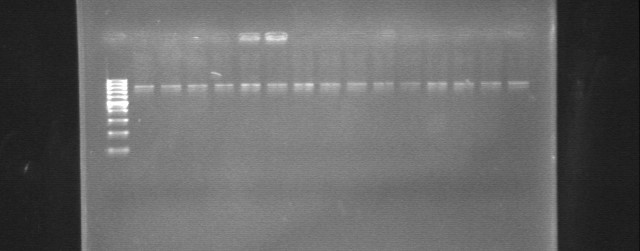

Supplement: S1 File — (DOCX) [file pone.0318090.s001.docx]
