# Supplementary material for: Lower termite (Coptotermes heimi) gut fibrolytic bacterial consortium: Isolation, phylogenetic characterization, fibre degradation potential and in vitro digestibility
Source: PLoS One. 2025 Mar 10;20(3):e0318090. doi: 10.1371/journal.pone.0318090 (PMC11892857; doi:10.1371/journal.pone.0318090)
Supplement: Attachment — Submitted filename: PONE-D-24-05915_R1.pdf [file pone.0318090.s004.pdf]

PLOS ONE

Lower termite (Coptotermes heimi) gut fibrolytic bacterial consortium: Isolation, phylogenetic characterization, fibre degradation potential and in vitro digestibility

--Manuscript Draft--

**Manuscript Number:**	PONE-D-24-05915R1
**Article Type:**	Research Article
**Full Title:**	Lower termite (Coptotermes heimi) gut fibrolytic bacterial consortium: Isolation, phylogenetic characterization, fibre degradation potential and in vitro digestibility
**Short Title:**	Phylogenetic and functional characterization of fibrolytic bacteria from lower termite gut
**Corresponding Author:**	Artabandhu Sahoo<br>NRCC: National Research Centre on Camel<br>Bikaner, INDIA
**Keywords:**	Lower termites, Coptotermes heimi, gut, 16S rRNA, fibrolytic enzymes, digestibility
**Abstract:**	Lower termites produce wide array of fibrolytic enzymes and serves as prospective microbial enzymes source for enhancing biodegradability of recalcitrant ligno-cellulosic fibrous feeds. The present study was aimed to isolate and characterize anaerobic fibrolytic bacteria from gut of termite Coptotermes heimi for screening promising isolates to improve fiber digestibility in ruminants. A total of 141 isolates were obtained from 97 termite gut samples, and 24 selected isolates (TM1 to TM24) isolates were characterized as obligatory anaerobes and catalase negative except, TM8, TM9, TM14 and TM22. Overall fibrolytic enzyme activity was highest in isolate TM23, TM6 and TM22. The highest FPase activity was observed in isolate TM5 (12.05 U/ml) while, it was lowest in TM19 (6.41 U/ml). The phylogenetic analysis depicted four major families i.e., Clostridiales, Bacillales, Lactobacillales and Enterobacterales under phyla Firmicutes and Proteobacteria. The in vitro dry matter digestibility of the substrate was increased by 9.4 to 36.0% with the inoculation of isolated bacterial strains. Among the screened isolates, TM6 exhibited highest ability to improve in vitro dry matter digestibility. The findings of the present study revealed that fibrolytic bacteria isolated from the termite gut can be used for commercial enzyme production or in rumen biotechnological application for enhancing fibrous feed utilization in ruminants.
**Order of Authors:**	Pankaj Kumar Kumawat<br>Srobana Sarkar<br>Satish Kumar<br>Artabandhu Sahoo
**Response to Reviewers:**	Dear Editor,<br>Thank you for your useful suggestions on the structure of our manuscript. The suggestions were helpful in improving our manuscript and we have addressed them in the revised version.<br>Here we are also providing the detailed responses to the comments and a word file has also been attached<br>CommentsResponse to comments<br>Line 14: Please provide the full name of the authorAgreed and complied<br>Line 15: spacing before "Principal".Corrected<br>Line 19: change to "The present study was aimed to..."Sentence modified as suggested<br>LIne 20: missing a spacing - of anaerobic; from gut; termite Coptotermes; inhabiting under; semi arid.Needful corrections done<br>Line 21: missing a spacing before "A total".Needful corrections done<br>Line 22: missing a spacing before "isolates".Needful corrections done<br>Line 23: activity.Corrected as suggested<br>Line 24: The highest........the lowest...Sentence modified as suggested<br>Line 25: missing a spacing before "phylogenetic".Needful corrections done<br>Line 26: missing a spacing before "In vitro"; between "substrate was".Needful corrections done<br>Line 27: change to "TM6 revealed the highest".Needful corrections done

	Line 28: missing a spacing - the gut of C. heimi.Needful corrections done<br>Line 30: missing a spacing before "utilization".Needful corrections done<br>Line 33: to improve.Needful corrections done<br>Line 33: poor quality of fibres.Needful corrections done<br>Line 34: missing a spacing before Coptotermes, gut.Needful corrections done<br>Line 37: into.Corrected as suggested<br>Line 40: higher and lower? incomplete sentence.Sentence completed<br>Line 41:harbour.Corrected as suggested<br>Line 41: no comma before and after "while".Needful corrections done<br>Line 41: contain..Corrected as suggested<br>Line 43: work, help.Corrected as suggested<br>Line 43: breaking down complexCorrected as suggested<br>Line 48: poor-quality. Delete "adding".Corrected as suggested<br>Line 50: have the ability.Corrected as suggested<br>Line 51: missing a spacing before "Furthermore".Corrected as suggested<br>Line 52: missing a comma between acetogenesis and methanogenesis. rewrite the last sentence.<br>Line 54: typo error - heimi.Sentence rewritten as suggested<br>Line 55: C. heimi.Needful corrections done<br>Line 56: C. heimi.Needful corrections done<br>Line 56: harbour a diverse.Corrected as suggested<br>Line 58: from the gut.Corrected as suggested<br>Line 59: of C. heimi.Corrected as suggested<br>Line 59: delete microbiota never studied beforeCorrected as suggested<br>Line 60: roughage-basedCorrected as suggested<br>Line 65: C. heimi.Corrected as suggested<br>Line 66: what type of nest? What are the criteria in the site/tree selection?The Coptotermes is part of family Rhinotermitidae are construct their nests within the wood they consume. These termites carve out intricate networks of tunnels within the wood, devoid<br>of any visible external entrances, except for temporary ones formed during swarming periods.<br>The tunnels within the nests are divided by partitions composed of their waste material and<br>are often covered or lined with a plaster-like substance also made from their waste.<br>The criteria used to identifying termite infested trees are as followed<br>1. Visible Damage<br>2. Frass or Termite Droppings<br>3. Mud Tubes<br>4. Wood Density and Moisture Content<br><br>Line 67: delete "trees". Replace "on March" with "in March". How did you collect the samples?Corrected as suggested<br>Line 80: 48 h.Corrected as suggested<br>Line 82: was repeated.....Corrected as suggested<br>Line 93: The experiment was conducted to estimate the enzymatic activity instead of the enzyme itself. Please rephrase the sentence.Corrected as suggested<br>Line 96: activities are expressed.Corrected as suggested<br>Line 104-105: rewrite.Sentence rewritten as suggested<br>Line 107: 39°C.Corrected as suggested<br>Line 110: rewrite.Sentence rewritten as suggested<br>Line 111-112: rewrite.Sentence rewritten as suggested<br>Line 113-116: rewrite.Sentence rewritten as suggested<br>Line 123: 10 d.Corrected as suggested<br>Line 125: was measured.Corrected as suggested<br>Line 128: Get the correct name of the kit.Needful corrections done<br>Line 133: missing a spacing between a numeric and a unit.Corrected as suggested<br>Line 134: How many different types of conditions?The conditions are mentioned in text and rewritten for clarity.<br>Line 135: 72ºCNeedful corrections done<br>Line 136: 1.5 KbNeedful corrections done<br>Line 145: isolates were processed.....replicates...Text rewritten for clarity.<br>Line 147: How many types of content?Text rewritten for clarity.<br>Line 150: each bottle........condition.Needful corrections done

	Line 152: 100 mL.Needful corrections done<br>Line 154: 24 h.Needful corrections done<br>Line 156: italic - in vitro. the samples were strained.....Needful corrections done<br>Line 157: -20°CNeedful corrections done<br>Line 160: missing a spacing - 6 ft.Needful corrections done<br>Line 160: SP-1200 and 1% phosphoric acid.Needful corrections done<br>Line 162: 150°CNeedful corrections done<br>Line 163: define gases air.Needful corrections done<br>Line 164: missing a spacing - CFAs mixture, 65 mM. Small letter for acetic acid.Corrected as suggested<br>Line 173: missing a spacing before "All".Corrected as suggested<br>Line 174: missing a spacing - their ability.Corrected as suggested<br>Line 175: missing a spacing - which confirms.<br>Line 176: missing a spacing before "All".Corrected as suggested<br>Line 182: rewrite the sentence.Sentence rewritten for clarity<br>Line 185: The endoglucanase activity of TM23 (Clostridium puniceum strain BL 70/20) was ...Corrected as suggested<br>Line 186: missing a spacing - and the highest. Delete "was showed by isolate TM23.Corrected as suggested<br>Line 186-197: only the scientific names are written in italic. The coding names should be written in a regular format.Agreed and complied<br>Line 191: The maximum activity was observed in TM18...Corrected as suggested<br>Line 198: missing a spacing - degradation potential.Corrected as suggested<br>Line 199: hours or h. Please standardise.Agreed and complied<br>Line 201: The coding names should be written in a regular format.Agreed and complied<br>Line 204; missing a spacing - 1500 bp. Explain why only 20 isolates were processed molecular identification.Corrected as suggested. All 24 isolates were processed for molecular identification, 20 was typographical error.<br>Line 205: missing a spacing - to nucleotide blast.Needful corrections done<br>Line 206-217: The coding names should be written in a regular format.Agreed and complied<br>Line 217: 24 sequences represent how many bacterial isolates?Sequences of 24 isolates were submitted to NCBI GenBank<br>Line 221: no italic for "hay". missing a spacing - straw by.Agreed and complied<br>Line 222: The coding names should be written in a regular format.Agreed and complied<br>Line 225 - 226: the highest, the lowest.Needful corrections done<br>Line 238 - 243: check all the spacing between words. A lot of grammatical errors. Rewrite the last sentence.Sentence rewritten for clarity<br>Line 250-251: spacing.Needful corrections done<br>Line 252-259: no italic for sp. or spa.Needful corrections done<br>Line 259-262: rewrite.Sentence rewritten for clarity<br>Line 263: from the gut of C. heimi.Corrected as suggested<br>Line 267: from the gut of .Corrected as suggested<br>Line 271-272: rewrite.Sentence rewritten<br>Line 272: to extract.Corrected as suggested<br>Line 295: the most......populations which include..Corrected as suggested<br>Line 296: delete "family".Corrected as suggested<br>Line 297: in the handgun of.Corrected as suggested<br>Line 298: a key role.Corrected as suggested<br>Line 299: in the fermentation processes.Corrected as suggested<br>Line 302: in the midgut of.Needful corrections done<br>Line 304: Firmicutes as the most.Corrected as suggested<br>Line 305: it was reported.Corrected as suggested<br>Line 307: small letter - revealed.Corrected as suggested<br>Line 308: had enhanced the.Corrected as suggested<br>Line 309: spacing between texts.Needful corrections done<br>Line 310: the highest.Needful corrections done<br>Line 319: from the gut.Corrected as suggested<br>Line 320: this is the first report.Needful corrections done<br>Line 321: the gut of C. heimiNeedful corrections done

Powered by Editorial Manager® and ProduXion Manager® from Aries Systems Corporation

	Regards<br>Artabandhu Sahoo
**Additional Information:**	
**Question**	**Response**
**Financial Disclosure**<br><br>Enter a financial disclosure statement that describes the sources of funding for the work included in this submission. Review the submission guidelines for detailed requirements. View published research articles from *PLOS ONE* for specific examples.<br><br>This statement is required for submission and **will appear in the published article** if the submission is accepted. Please make sure it is accurate.<br><br>**Funded studies**<br>Enter a statement with the following details:<br>• Initials of the authors who received each award<br>• Grant numbers awarded to each author<br>• The full name of each funder<br>• URL of each funder website<br>• Did the sponsors or funders play any role in the study design, data collection and analysis, decision to publish, or preparation of the manuscript?<br><br>Did you receive funding for this work?	The author(s) received no specific funding for this work.
**Competing Interests**<br><br>Use the instructions below to enter a competing interest statement for this submission. On behalf of all authors, disclose any competing interests that could be perceived to bias this work—acknowledging all financial support and any other relevant financial or non-financial competing interests.<br><br>This statement is required for submission and **will appear in the published article** if the submission is accepted. Please make	The authors have declared that no competing interests exist.

sure it is accurate and that any funding sources listed in your Funding Information later in the submission form are also declared in your Financial Disclosure statement.<br><br>View published research articles from *PLOS ONE* for specific examples.<br><br>**NO authors have competing interests**<br><br>Enter: *The authors have declared that no competing interests exist.*<br><br>**Authors with competing interests**<br><br>Enter competing interest details beginning with this statement:<br><br>*I have read the journal's policy and the authors of this manuscript have the following competing interests: [insert competing interests here]*<br><br>* typeset	
**Ethics Statement**<br><br>Enter an ethics statement for this submission. This statement is required if the study involved:<br><br>• Human participants<br>• Human specimens or tissue<br>• Vertebrate animals or cephalopods<br>• Vertebrate embryos or tissues<br>• Field research<br><br>Write "N/A" if the submission does not require an ethics statement.<br><br>General guidance is provided below. Consult the submission guidelines for detailed instructions. **Make sure that all information entered here is included in the Methods section of the manuscript.**	N/A

**Format for specific study types**<br><br>**Human Subject Research (involving human participants and/or tissue)**<br>• Give the name of the institutional review board or ethics committee that approved the study<br>• Include the approval number and/or a statement indicating approval of this research<br>• Indicate the form of consent obtained (written/oral) or the reason that consent was not obtained (e.g. the data were analyzed anonymously)<br><br>**Animal Research (involving vertebrate animals, embryos or tissues)**<br>• Provide the name of the Institutional Animal Care and Use Committee (IACUC) or other relevant ethics board that reviewed the study protocol, and indicate whether they approved this research or granted a formal waiver of ethical approval<br>• Include an approval number if one was obtained<br>• If the study involved *non-human primates*, add *additional details* about animal welfare and steps taken to ameliorate suffering<br>• If anesthesia, euthanasia, or any kind of animal sacrifice is part of the study, include briefly which substances and/or methods were applied<br><br>**Field Research**<br><br>Include the following details if this study involves the collection of plant, animal, or other materials from a natural setting:<br>• Field permit number<br>• Name of the institution or relevant body that granted permission	
**Data Availability**<br><br>Authors are required to make all data underlying the findings described fully available, without restriction, and from the time of publication. PLOS allows rare exceptions to address legal and ethical concerns. See the PLOS Data Policy and FAQ for detailed information.	Yes - all data are fully available without restriction

Powered by Editorial Manager® and ProduXion Manager® from Aries Systems Corporation

A Data Availability Statement describing where the data can be found is required at submission. Your answers to this question constitute the Data Availability Statement and **will be published in the article**, if accepted.<br><br>**Important:** Stating 'data available on request from the author' is not sufficient. If your data are only available upon request, select 'No' for the first question and explain your exceptional situation in the text box.<br><br>Do the authors confirm that all data underlying the findings described in their manuscript are fully available without restriction?	
**Describe where the data may be found in full sentences. If you are copying our sample text, replace any instances of XXX with the appropriate details.**<br><br>• If the data are **held or will be held in a public repository**, include URLs, accession numbers or DOIs. If this information will only be available after acceptance, indicate this by ticking the box below. For example: *All XXX files are available from the XXX database (accession number(s) XXX, XXX.*).<br>• If the data are all contained **within the manuscript and/or Supporting Information files**, enter the following: *All relevant data are within the manuscript and its Supporting Information files.*<br>• If neither of these applies but you are able to provide **details of access elsewhere**, with or without limitations, please do so. For example:<br><br>*Data cannot be shared publicly because of [XXX]. Data are available from the XXX Institutional Data Access / Ethics Committee (contact via XXX) for researchers who meet the criteria for access to confidential data.*<br><br>*The data underlying the results presented in the study are available from (include the name of the third party*	All relevant data are within the manuscript and its Supporting Information files.

and contact information or URL).<br>• This text is appropriate if the data are owned by a third party and authors do not have permission to share the data.<br><br>* typeset	
Additional data availability information:	Tick here if the URLs/accession numbers/DOIs will be available only after acceptance of the manuscript for publication so that we can ensure their inclusion before publication.

Powered by Editorial Manager® and ProduXion Manager® from Aries Systems Corporation

Manuscript

Click here to access/download;Manuscript;PLOS ONE final Main Manuscript -Clean.docx

Lower termite (*Coptotermes heimi*) gut fibrolytic bacterial consortium: Isolation, phylogenetic characterization, fibre degradation potential and *in vitro* digestibility

Pankaj Kumar Kumawat[1,3], Srobana Sarkar[1*], Satish Kumar[3,4], Artabandhu Sahoo[1,2*]

[1]ICAR- Central Sheep and Wool Research Institute, Avikanagar, Rajasthan

[2] ICAR- National Research Centre on Camel, Bikaner, Rajasthan

[3]Mewar University, Rajasthan

[*]**Corresponding authors:** Artabandhu Sahoo, Email address: sahooarta1@gmail.com; Srobana Sarkar, Email address: sarkarsrobana@gmail.com

Phone No.: 01437- 220162, Fax: 01437- 220163

Pankaj Kumar Kumawat: PhD scholar, Mewar University, Rajasthan and Senior Research Fellow, ICAR-Central Sheep and Wool Research Institute, Avikanagar, Rajasthan.

Srobana Sarkar: Scientist, ICAR-Central Sheep and Wool Research Institute, Avikanagar, Rajasthan.

A. Sahoo: Principal Scientist &Director, ICAR- National Research Centre on Camel, Bikaner, Rajasthan.

Satish Kumar: Principal, Mata Basanti Devi School of Biosciences & Biotechnology, Agra.

[4]**Present address**- Mata Basanti Devi School of Biosciences & Biotechnology, Agra

Abstract

Lower termites produce wide array of fibrolytic enzymes and serves as prospective microbial enzymes source for enhancing biodegradability of recalcitrant ligno-cellulosic fibrous feeds. The present study was aimed to isolate and characterize anaerobic fibrolytic bacteria from gut of termite *Coptotermes heimi* for screening promising isolates to improve fiber digestibility in ruminants. A total of 141 isolates were obtained from 97 termite gut samples, and 24 selected isolates (TM1 to TM24) isolates were characterized as obligatory anaerobes and catalase negative except, TM8, TM9, TM14 and TM22. Overall fibrolytic enzyme activity was highest in isolate TM23, TM6 and TM22. The highest FPase activity was observed in isolate TM5 (12.05 U/ml) while, it was lowest in TM19 (6.41 U/ml). The phylogenetic analysis depicted four major families i.e., *Clostridiales*, *Bacillales*, *Lactobacillales* and *Enterobacterales* under phyla *Firmicutes* and *Proteobacteria*. The *in vitro* dry matter digestibility of the substrate was increased by 9.4 to 36.0% with the inoculation of isolated bacterial strains. Among the screened isolates, TM6 exhibited highest ability to improve *in vitro* dry matter digestibility. The findings of the present study revealed that fibrolytic bacteria isolated from the termite gut can be used for commercial enzyme production or in rumen biotechnological application for enhancing fibrous feed utilization in ruminants.

Keywords Lower termites, *Coptotermes heimi,* gut, 16S rRNA, fibrolytic enzymes, digestibility

Introduction

Termites acts as “ecosystem engineers” because they help in efficient degradation of complex plant biomass into resourceful bioenergy in comparison to other invertebrates [1,2]. Their potential to degrade fibrous polysaccharide depends on the dual enzymatic machinery of termite and its gut microbial symbionts, which makes them an ideal source of microbial enzymes [3, 4]. Termites have the ability to degrade around 65 to 87% hemicelluloses and 74 to 99% cellulose [5]. They are intricately grouped into higher and lower termites [6], the higher termites harbour only prokaryotes and lacks protists [7,8,5], while the, lower termites contain both protists and bacteria in their gut [9]. The microbes present in termite gut work in consortium and help in breaking down complex carbohydrates [10,11]. However, the abundance of microbes inhabiting in the gut of termites not only depends on their hierarchy but also on their geographical location and available vegetation [12].

Utilization of agro-industrial by-products and poor-quality fibrous feeds in animal nutrition plays a pivotal role in sustainable agriculture. Effect of several treatments including physical, chemical and biological have been studied for enhancing utility of poor-quality feed resources in ruminants [13,14]. In ruminants, exogenous fibrolytic enzymes aid in increasing the digestibility of fiber[15]. Earlier studies reported that bacteria isolated from termite gut have the ability to disintegrate lignin barrier and digest carbohydrate polymers [13,16-18]. Additionally, because the environments in the rumen and termites' guts are similar in terms of anaerobiosis, reductive acetogenesis, methanogenesis, and production of volatile fatty acids, the bacteria isolated from termite guts are better suited as microbial feed additives to enhance the utilization of recalcitrant ligno-cellulosic fibrous crop residues. [16]. *Coptotermes heimi* are the most predominant lower termites the in semi-arid regions of India [19]. Till date, little information is available regarding origin and evolution of *Coptotermes heimi* and no

research has been done on their gut microbiota's abundance.. It is hypothesized that *Coptotermes heimi* harbour a diverse species of fiber degrading bacteria with promising fibrolytic enzyme potential. Therefore, the present study was undertaken to isolate and identify novel fibrolytic bacteria from the gut of *Coptotermes heimi* and to evaluate their capacity to break down plant fibre in terms of enzymatic activity and potential to improve digestibility of roughage-based diets.

Materials and methods

Specimen collection

The present study was conducted at and surrounding areas of ICAR-Central Sheep and Wool Research Institute campus, Rajasthan, India. The termite samples of *Coptotermes heimi* were collected from nests on *Ailanthus excelsa*, *Azadirachta indica*, *Albizia lebbeck*, *Cassia fistula*, *Mangifera indica* and *Eucalyptus camaldulensis*. A total of ninety-seven samples of wood eating termites were collected (in March 2019) and transported to laboratory and washed with water to remove dirt. Thereafter, the termite samples were washed with 70% ethanol followed by distilled water for surface sterilization. The termite's head was removed using sterilized needle and the entire gut was excised out [20]. The gut samples were macerated and processed for isolation of fibre degrading bacteria.

Isolation of fibre degrading bacteria

For isolation of fiber degrading bacteria, M10 medium was used with slight modification which comprised of 15 ml mineral solution-I (0.3% K_2HPO_4), 15 ml mineral solution –II (0.3% KH_2PO_4, 0.6% $(NH_4)_2SO_4$, 0.6% NaCl, 0.06% $MgSO_4.7H_2O$, 0.0795% $CaCl_2.2H_2O$), 0.05% tryptone, 0.05% yeast extract powder, 0.1% resazurine, 0.2% microcrystalline cellulose, 0.1 ml Pfenning trace elements, 0.31 ml volatile fatty acid mixture, 0.8% sodium bicarbonate ($NaHCO_3$), 20 ml clarified rumen liquor, 0.05% cystine hydrochloride (Cys-HCL). The collected samples were serially diluted in anaerobic diluents [21] up to 10^8 cells/ml. From each dilution, around 0.5 ml of inoculum was inoculated in M10 agar medium (1.5% agar) in Hungate tubes (5 mL) using disposable syringe. The agar tubes were rolled on ice bed for solidification and incubated at 39°C for 48 h. Presumptive colonies of different colour, size and shape were picked and inoculated in anaerobically prepared M10 broth medium for sub-culturing in an anaerobic chamber (Bioxia, India). The above process was repeated at least for 4 times till monoculture was obtained. The pure cultures isolated were stored in 80% anaerobic glycerol stock at -80°C for further use.

Morphological and biochemical characterization of isolates

The isolates were characterized morphologically by Gram staining [22] and biochemically by tests like anaerobiosis, catalase, gas production, motility, H_2S production, indole, methyl red and sugar fermentation [23]. The results obtained were compared with Bergey's Manual of Determinative Bacteria for identification [24].

Fibrolytic enzyme activity

The experiment was conducted to estimate the fibrolytic enzymatic activity, 96 h old bacterial culture, in three replicates, were treated with lysozyme and sonicated for 6 min with 30-sec pulse rate under ice-cool condition and centrifuged at 14,000×g [23]. The supernatant obtained was used as a crude enzyme extract for estimation of fibrolytic enzymes like carboxymethyl cellulase (CMCase), endoglucanase, avicelase (Exoglucanase), α-Amylase (1,4-Gluanohydrolase), xylanase (Endo-1,4-ß-xylanase) and β-glucosidase, filter paper degrading assay (FPase) [25, 26]. The endoglucanase, exoglucanase, FPase, and α-Amylase enzyme activities were expressed as μmol glucose released per min per ml (U/ml) and xylanase activity was expressed as μmole xylose released per min per mL (U/mL). The substrate used for estimation of endoglucanase, exoglucanase, α-Amylase, and xylanase were 1% carboxymethyl cellulose (Sigma-Aldrich, USA), 1% avicel (Sigma-Aldrich, USA), 1% starch (Sigma-Aldrich, USA), and 1% xylan (Sigma-Aldrich, USA),respectively while,the FPase activity was determined using 50 mg Whatman filter paper as the substrate. To calculate the activity of endoglucanase, exoglucanase, α-Amylase, and FPase enzyme, 0.1% glucose (Sigma-Aldrich, USA) was used as a standard whereas, for xylanase enzyme 0.1% xylose was used as a standard (Sigma-Aldrich, USA). The test reaction consisted of 0.05 ml enzyme extract, 0.05 ml substrate, and 0.1 ml phosphate buffer. The control reaction was identical to the test with the exception of the enzyme extract, which was denatured in the control reaction. The contents in the tubes were mixed properly and kept at 39°C for 60 min. Following incubation, 0.5 ml di-nitro salicylic acid reagent (Sigma-Aldrich, USA) was added to the tubes and kept in boiling water bath for 10 min. After 10 min, 0.1 mL of Rochelle salt was added to the tubes and allowed to cool to room temperature and the absorbance was measured at 575 nm against blank. For the determination of β-glucosidase enzyme activity, 0.9 ml of 0.1% PNPG (p-nitrophenol ß-D-glucopyranose) (Hi-media, India) was incubated for 10 minutes with 0.1 ml of enzyme extract as the substrate. The control tubes contained 0.1 ml denatured enzyme extract as the substrate. Following incubation, 1.0 ml of 2% sodium carbonate was added to the tubes to stop the reaction and absorbance was measured at 400 nm against blank. For the preparation of standard curve, 0.002% p-nitrophenol was used (Sigma-Aldrich) and the enzyme activity was expressed as μmole p-nitrophenol released per min per ml.

Filter Paper degradation potential

For assessing crystalline cellulose degrading potential of the isolates, M10 medium was prepared anaerobically and 25 × 5 mm strip of filter paper (Whatman No. 1) was added in the media and autoclaved. The isolates were inoculated in roll tubes containing filter paper media and incubated at 39°C for 10 d. Degradation of crystalline cellulose was determined as amount of filter paper degraded at an interval of 24 h up to 10 d till complete degradation. Degradation potential of test cultures was measured after comparison with un-inoculated control samples [23].

Molecular characterization and sequence homology analysis of 16S rRNA gene

Genomic DNA was extracted from the isolates by using DNeasy PowerLyzer PowerSoil Kit (Qiagen, Germany) as per manufacturer's instructions. The 16S rRNA gene was amplified from the isolated genomic DNA with 27F 5'-AGAGTTTGATCCTGGCTCAG-3' (forward) and 1495R 5' CTACGGCTACCTTGTTACG-3' (reverse) universal 16S rRNA gene primers. A polymerase chain reaction (PCR) was carried out in an Eppendorf thermal cycler (Germany), each reaction mixture (50 μL) consisted of 10X reaction buffer, 10 μM primers, 10 mM

dNTPs, 1.5 U Taq DNA polymerase (Thermo Scientific, Germany), and up to 100 ng DNA template. The annealing temperature was 52◦C and the PCR product size was 1.5 kb. The quality of amplified product was checked by agarose gel electrophoresis. The amplified products were gel purified using GeneJET Gel extraction kit (Cat#K0691, Thermo Scientific, Germany) as per manufacturer's protocol. The purified amplified product was sequenced as per sanger sequencing method by an outsourcing agency (Eurofins, Bengaluru). The nucleotide sequences obtained were trimmed using edit seq software (DNA STAR) and analysed for nearest valid taxon and species using BLAST algorithm [27]. All the aligned sequences were submitted to GenBank. The evolutionary history was inferred using the Neighbor-Joining method. The tree was drawn to scale with branch lengths (above the branches) in the same units as those of the evolutionary distances used to infer the phylogenetic tree. The evolutionary distances were computed using the Maximum Composite Likelihood method and were in the units of the number of base substitutions per site and the analysis involved 40 nucleotide sequences. All ambiguous positions were removed for each sequence pair (pairwise deletion option). There was a total of 1605 positions in the final dataset and evolutionary analysis was conducted in MEGA11.

***In vitro* true digestibility**

To evaluate the digestibility potential of the screened isolates an *in vitro* gas production test was conducted [28,29]. Around 500±10 mg of dried *Cenchrus ciliaris* and *Vigna mungo* straw (80:20) was used as a substrate. Both live culture and autoclaved cultures (control) were used for each isolate. Each treatment had 4 replications. Around 40 ml incubation medium was administered anaerobically in a 100 ml glass syringe along with 3.0 ml of culture. After 48 h incubation at 39ºC, the contents of the syringe were refluxed with 100 ml of neutral detergent solution (sodium lauryl sulphate - 30 g, disodium ethylene diamino tetra acetate - 18.61 g, sodium borate decahydrate - 6.81 g, disodium hydrogen phosphate (anhydrous) - 4.56 g, triethyleneglycol − 10 ml, distilled water - 990 ml per L) in a spoutless beaker, filtered through pre-weighed gooch crucibles and weighed after 24 h oven drying to determine *in vitro* dry matter digestibility [30].

Short chain fatty acid production

After conducting *in vitro* gas production test as mentioned above, the content of the syringe was strained with muslin cloth, collected in scintillation vials and preserved in -20°C by adding 0.25% m-phosphoric acid (Hi-media) in the ratio of 9:1. For estimation of short chain fatty acids (SCFAs), the preserved samples were centrifuged at 5000 × gfor 10 min and the clear supernatant obtained was injected into the gas chromatogram (GC). The GC (Dani Make, GC-1000, Italy) was equipped with a stainless-steel column of 6ft length and 1/8 inch diameter, packed with 10% SP-1200 and 1% phosphoric acid (Supelco). The temperature of the injector and detector was 220°C and 250°C, respectively while, the oven temperature was standardized at a gradient of 115°C to 150°C for separation of each molecule. Nitrogen gas was used as a carrier with a pressure of 0.8 bar while, the pressure of air and hydrogen was 1.0 and 0.8 bar, respectively. The standard used was prepared by mixing 65, 20, 3, 8, 2 and 2 mM of acetic acid, propionic acid, iso-butyric acid, butyric acid, iso-valeric acid and valeric acid, respectively.

Statistical analysis

The data obtained were analyzed using one-way analysis of variance (ANOVA).Tukey's b test was used for the post-hoc comparison of different treatment means and statistical differences were considered significant at $P<0.05$.

Results

Morphological and biochemical characterization

Out of 141 isolates, 24 (designated as TM1- TM24) were selected on the basis of formation of clear zones on Congo red plates for further microscopic and biochemical characterization (Table 1). All the isolates were tested for anaerobiosis and catalase reaction for assessing their ability to tolerate anoxic environment. Twenty of the 24 isolates showed positive anaerobiosis and negative catalase reaction, indicating that they are obligatory anaerobes while, the other 4 isolates were facultative anaerobes with positive catalase reactions.. Except for TM12, all the isolates produced hydrogen sulfide gas by the converting sulfur to sulfide.. None of the isolates produced indole from the reductive deamination of tryptophan, hence all the isolates were indole negative.. All the isolates were able to utilize mannose, sucrose, glucose, carboxy-methyl cellulose, lactose, arabinose, starch, maltose, fructose, xylose and xylan for growth except for ribose sugar (Table 2).

Fibrolytic enzyme activity

In this study, endoglucanase, exoglucanase, FPase, β-glucosidase, α-amylase, and xylanase enzyme assays were performed to evaluate the fibrolytic potential of the isolates (Table 3). The isolates showed varying degree of fibrolytic enzyme activity with different substrates like carboxymethyl cellulose, avicel, filter paper, p-nitrophenyl-β-glucoside, starch and xylan. The endoglucanase activity ranged from 4.83 to 7.24 U/ml and highest activity was shown by isolate TM23. The exoglucanase activity among the isolates ranged from 4.24 to 8.40 U/ml. highest exoglucanase activity was showed by TM6 and lowest by TM19. The FPase activity was highest in isolate TM5 (12.05 U/ml)and, lowest in TM19 (6.41 U/ml). Maximum β-glucosidase activity was observed in TM18 and lowest in TM1. The α-amylase activity in the isolates ranged from 2.04 to 12.42 U/mL, where, highest and lowest activity was observed in isolate TM22 and TM3, respectively. Xylanase activity ranged from 0.055 to 12.00 U/ml, maximum being in TM5and minimum in TM20.

Filter paper degradation potential

In the present study, filter paper degradation potential of the isolates was determined at regular intervals of 24 h up to 10 d following the inoculation of bacterial isolates in filter paper strip media (Table 4). Among the isolates, TM5, TM9 and TM13 degraded filter paper in 7 days compared to others and these isolates also possessed higher FPase activity compared to other isolates.

Molecular identification and evolutionary relationships of isolates

The PCR product of ~ 1500 bp size obtained after 16S rRNA gene amplification (Figure 1) was considered for all the isolates for molecular identification (Table 5). The isolates TM1 and TM7 were 99% similar to *Bacillus megaterium* IAM 13418. The isolates TM2 and TM4 had 98% similarity *Citrobacter freundii* JCM1657. The

isolates TM10 and TM13 were 99% and 98% similar to *Clostridium termitidis*, respectively. The isolates TM16 and TM 18 were 97% and 99% similar to *Lactococcus nasutitermitis* M19, while, TM17 and TM24 showed 90% and 99% similarity with *Clostridium sporogenes* JCM 1416. The isolates TM19 and TM20 were 98% similar to *Bacillus circulans* ATCC 4513. Comparison in similar line, isolate TM3 was 99% similar with *Citrobacter farmeri* CDC 2991, TM5 was 99% similar with *Bacillus flexus* Tl-1, TM8 was 99% similar to *Bacillus licheniformis* ATCC14580, TM9 was 98% similar to *Bacillus cereus* ATCC 14579, TM11 was 100% similar to *Clostridium saccharobutylicum* P262, TM12 was 97% similar to *Clostridium indolis* 7, TM14 was 98% similar to *Bacillus oleronius* DSM9356, TM15 was 96% similar to *Clostridium cellulovorans* 743B, TM21 was 98% similar to *Ruminiclostridium papyrosolvens* DSM 2782, TM22 was 96% similar to *Brevibacillus brevis* DSM 30 and TM23 was 96% similar to *Clostridium puniceum* BL 70. All the 16S rRNA gene sequences (TM1 - TM24) were submitted to NCBI GenBank and accession no. MT356127 to MT356150 were obtained.

In vitro dry matter digestibility

As depicted in Figure 5, inoculation of bacterial isolates from termite gut enhanced (P<0.05) the dry matter (DM) digestibility of substrate containing *Cenchrus ciliaris* hay and *Vigna mungo* straw by 9 to 36% in comparison to control. Inoculation of isolate TM6 resulted in maximum increase of DM digestibility than other bacterial isolates.

Short chain fatty acid production

Total short chain fatty acid production by the isolates ranged from 64.19 mM to 117.73 mM (Table 6). The concentration of acetate, propionate, iso-butyrate, butyrate, iso-valerate and valerate produced by the isolates ranged from 50.37 mM to 83.59 mM, 6.92 mM to 17.87 mM, 1.10 mM to 2.77 mM, 3.08 mM to 10.34 mM, 0.70 mM to 1.56 mM and 0.39 mM to 1.61 mM, respectively. Isolate TM6 produced highest (P<0.01) acteate (83.59 mM), propionate (17.87 mM), iso-bytyrate (2.77 mM), butyrate (10.34 mM), iso-valerate (1.56 mM) and valerate (1.61 mM) compared to other isolates. Lowest SCFAs (64.19 mM) was produced by isolate TM7. The isolates TM5 and TM11 produced the lowest amount of propionate and butyrate. The acetate to propionate ratio (C2:C3) was significantly higher in isolate TM5 and TM11 .

Discussion

The diverse symbiotic bacterial species found in termite guts contributes to the breakdown of lignocellulose, oligosaccharides, and aromatic compounds, suggesting that these bacteria are important for the digestion of fiber. [31,32]. Till date, several natural polymer-degrading microbes have been isolated and characterized from the termite gut for degradation of plant biomass to biofuel. However, the present study aimed identification of potentially useful anaerobic fibre degrading bacteria from the gut of wood feeding lower termites inhabiting in the semi-arid environments so that these isolates could be used to improve the usage of fibrous plant biomass in ruminants' feeding.

The isolates showed diverse colony characteristics in terms of size, shape and colour. Fourteen isolates were gram-positive rods, while, remaining 8 were gram negative rods and 2 were gram positive cocci. The presence of cellulolytic bacilli and cocci bacteria in different species of termites is not uncommon [33-36]. The, isolates were also biochemically characterized and illustrated using Bergey's manual of determinative bacteriology following morphological screening [37]. In consistent with the present results, several studies have reported similar biochemical and carbohydrate utilization characteristics from the gut of termites *Reticulitermes santonensis* [38], *Zootermopsis angusticollis* [39], *Anacanthotermes vagans* [40] and *Microcerotermes diversus* [13].

Termites degrade fibre either by their own enzymatic machinery or from enzymes of symbiotic microbes residing in their gut. The association between termites and microbial symbionts in gut makes it an efficient source of enzymes like cellulase, hemicellulase and auxillary enzymes [31]. Similar to the present findings, a prior study found that bacteria isolated from the gut of higher termite (*Nasutitermes* sp.) showed endoglucanase activity ranging from 0.5 to 6.8 U/ml [41]. Furthermore, *Bacillus* sp was also isolated from wood eating termites *Neotermes* spp and *Cryptotermes brevis* exhibiting endoglucanase activity of 0.29 and 1.78 U/ml, respectively [35,42]. Avicel was utilized as a substrate for exoglucanase activity since the cell walls of plants are made of microcrystalline cellulose that is interlaced with biopolymers [43]. In line of the present findings, an exoglucanase activity of 7.93 to 18.76 U/ml was reported in bacteria isolated from hindgut of wood eating termite *Amitermes evuncifer silvestri* [34]. A similar FPase activity of 16.18 U/ml was reported in bacteria isolated from the gut of termite *Odontotermes formosanus* [44,45]. However, in the present study bacteria isolated from the gut of *C. heimi* have higher exoglucanase activity than endoglucanase activity, which depicts that these isolates are better degraders of recalcitrant plant fiber that contains high crystalline cellulose. The β-glucosidase enzyme is found in the digestive system of many insects and lower termites, i.e.mostly present in the salivary gland of termites rather than their gut and helps in their energy metabolism [46]. However, bacteria isolated from the gut of higher termite *Macrotermes annandalei* revealed presence of β-glucosidase genes that may be involved in the host's fiber digestion [47]. Similarly, *Bacillus* spp. isolated from termite gut had β-glucosidase activity varying between 0.6 and 1.5 U/ml [48]. In lower termites, 81% of amylase activity was detected in salivary glands [49], however, microbial origin amylases were also found in the gut of termites produced by the symbiotic bacteria inhabiting in the gut [50]. Several studies have reported that higher termites have gut microbial population which produces amylase and it agrees with the present findings [46, 51-53]. Degradation of hemicelluloses requires enzyme endo-1, 4-xylanase and studies have reported community of xylanolytic bacteria in the gut of termites [54]. Similar to our findings, bacteria belonging to the *Bacillus* group with promising xylanolytic activity have also been isolated from wood eating termite *Cryptotermes brevis* [35].

Filter paper contains both amorphous and crystalline cellulose. Degradation of filter paper by any bacteria indicates its cellulolytic potential. Previous studies also revealed filter paper degradation potential of bacterial species isolated from termites, however, their filter paper degradation potential was lower as compared to the present findings [55,56]. The exoglucanase enzyme is mainly responsible for degradation of crystalline cellulose and it is directly related to filter paper degradation potential of the isolates. Thus, an increased filter paper degrading potential of the isolates in comparison to previous reports signify their importance with high

exoglucanase activity and could be promising inoculant for commercial enzyme production aimed at plant fiber degradation.

The evolutionary study revealed that 87% of the isolates belonged to the phyla *Firmicutes* and remaining to *Proteobacteria* (Figure 2). The majority of isolates from these two phyla belonged to the orders *Bacillales*, *Lactobacillales*, *Enterobacterales*, and *Clostridiales* . Isolates under the order *Clostridiales* belonged to the families *Hungaticlostridiaceae, Clostridiaceae* and *Lachnospiraceae*. Besides the order *Clostridiales,* some isolates also belonged to order *Bacillales, Lactobacillales* and *Enterobacterales* under the family *Bacillaceae, Streptococaceae, Enterococcaceae* and *Enterobacteriaceae*, respectively (Figure 3). In the present study, isolates were mostly from *Clostridiaceae* family followed by *Bacillaceae, Enterobacteriaceae, Streptococcaceae* and *Enterococcaceae*. Earlier reports also observed similar grouping in the phylum *Firmicutes* and order *Clostridiales* [57]. The 16S rRNA gene-based identification (Figure 4) also revealed that *Fimicutes* phyla and *Clostridia* clustar was the most abundant bacterial population including major representatives of *Peptococcaceae, Gracilibacteraceae, Clostridiaceae* and *Ruminococcaceae family*. In a previous study *Bacteroidetes*, *Fibrobacteres* and *Proteobacteria* were also reported to be present in the hindgut of wood-eating termites [58]. The *Clostridales* group contained members of the *Clostridiaceae* family, which is important for the breakdown of plant biomass and indicates an isolate's capacity for cellulolysis [58].. Majority of the bacterial populations in the midgut of termite *Nasutitermes corniger* belongs to the phylum *Firmicutes* and are members of the family *Lachnospiraceae* which are capable of breaking down cellulose and hemicelluloses [59]. The genetic library of termites fed xylan revealed *Firmicutes* as the most prevalent phylum, and this suggests that *Firmicutes*-related bacteria are able to degrade hemicelluloses [60]. Moreover, it was reported that a high abundance of *Enterobacteriales* was found in the foregut of termites (*Bullitermes* sp.) after 16S rRNA metagenomic investigation of the community structures of bacteria in the gut [58].

In the present study, inoculation of screened isolates enhanced nutrient digestibility of the substrate containing *Cenchrus ciliaris* and *Vigna mungo* straw. It is reported that microbial enzymes which break down structural fibers increases the amount of sugars available to rumen bacteria, which enhances nutrient digestibility [61]. Several studies have reported a symbiotic relationship between facultative and obligate anaerobes in the termite gut, where, facultative anaerobes scavenge oxygen that penetrates from gut exoskeleton which is essential for fibrolytic activity of obligatory anaerobes [62,63]. The isolate TM6 had highest endo and exoglucanase activity which resulted in enhanced DM digestibility. Likewise, a study reported improvement in nutrient digestibility of substrate containing wheat straw and date leaves by inoculation of symbiotic bacteria isolated from the termite gut [13]. However, no effect was observed on *in vitro* rumen fermentation and fibre degradation when lignocellulose-degrading bacteria isolated from termite gut was innoculated with sheep rumen fluid [63]. The plant fiber containing ligno-cellulosic and hemicellulosic complexes needs to be broken down for releasing the carbohydrate moiety as energy source and carbon-skeleton for the multiplication of rumen microbial population that aid in nutrient availability to ruminants for increased productivity [15]. Thus, promising bacterial isolates from the termite gut with enhanced fiber-degrading activity could be a potential source for *in vitro* and/or *in vivo* manipulation in maximizing fibrous-feed utilization for animal production.

Conclusions

Till date, this is the first study regarding the existence of isolates with varying fibrolytic enzyme activity in the gut of the wood-eating termite *Coptotermes heimi*. Phylogenetic analysis depicted four major families i.e., *Clostridiales, Bacillales*, *Lactobacillales* and *Enterobacterales* under phyla *Firmicutes* and *Proteobacteria* with the ability to produce cellulolytic and xylanolytic enzymes. Furthermore, inoculation of isolated bacterial strains enhanced digestibility of roughage-based diet up to 36% and maximum improvement was observed in case of TM6. Although, further studies are required to understand the interaction of these isolates with the rumen microbiota if they are used as direct fed microbial for improving degradation of poor quality roughages in ruminants. Hence, *in vivo* studies are required to validate the effect of these exogenous isolates in rumen environment for confirming their efficacy to convert low nutritive biomass as quality ruminant feed. Nonetheless, the identified isolates could serve as promising inoculants in commercial enzyme production aimed at in situ plant-fiber degradation.

Author's contributions: PKK performed isolation and characterisation of isolates, genomic and phylogenetic analysis, SS writing of original draft, data analysis and data interpretation, SK contributed to text preparation and revised the manuscript, AS helped in conceptualization, fund acquisition and supervised the study. All authors read and approved the final version of the manuscript.

Institutional Review Board Statements: Not Applicable

Informed Consent Statement: Not Applicable

Data Availability Statement: All relevant data are within the manuscript.

Acknowledgments: Authors are thankful to the Vice Chancellor, Mewar University, Chittorgarh and Director, ICAR-Central Sheep and Wool Research Institute for providing necessary research facilities required during this study.

Conflicts of Interest: The authors declare no competing interest.

References

1. Febrianto F, Pranata AZ, Septiana D, Arinana A, Gumilang A, Hidayat W, Jang J, Lee S, Hwang W and Kim N. Termite resistance of the less known tropical woods species grown in West Java Indonesia. J Korean Wood Sci Technol. 2015; 43:248-257.
2. Thapa S, Mishra J, Arora N, Mishra P, Li H O′ Hair J, Bhatti S and Zhou S. Microbial cellulolytic enzymes: diversity and biotechnology with reference to lignocellulosic biomass degradation. Rev Environ Sci Biotechnol. 2020;19:621-648.
3. Auer L, Lazuka A, Sillam-Dussès D, Miambi E, O'Donohue M and Hernandez-Raquet G. Uncovering the potential of termite gut microbiome for lignocellulose bioconversion in anaerobic batch bioreactors. Front Microbiol. 2017;8:2623.

4. Victorica MR, Soria MA, Batista-García RA, Ceja-Navarro JA, Vikram S, Ortiz M Ontañon O, Ghio S, Martínez-Ávila L, García OJQ and Etcheverry C. Neotropical termite microbiomes as sources of novel plant cell wall degrading enzymes. Sci Rep. 2020;10:1-14.
5. Brune A. Symbiotic digestion of lignocellulose in termite guts. Nat Rev Microbiol. 2014;12:168-180.
6. Inward D, Beccaloni G, Eggleton P. Death of an order: a comprehensive molecular phylogenetic study confirms that termites are eusocial cockroaches. Biol Lett. Jun. 2007;22:331-5.
7. Slaytor M. Cellulose digestion in termites and cockroaches: what role do symbionts play?. Com Biochem Physiol Part B: Com Biochem. 1992;103:775-784.
8. Tokuda G and Watanabe H. Hidden cellulases in termites: revision of an old hypothesis. Biol lett. 2007;3:336-339.
9. Ni J and Tokuda G. Lignocellulose-degrading enzymes from termites and their symbiotic Microbiota. Biotechnol Adv. 2013;31:838-850.
10. Husseneder C, Ho HY, Blackwell M. Comparison of the bacterial symbiont composition of the Formosan subterranean termite from its native and introduced range. The Open Microbiol J. 2010; 4:53.
11. Peterson BF, Scharf ME. Lower termite associations with microbes: synergy, protection, and interplay. Front microbiol. 2016;8:7:422.
12. Khan MA, Ahmad W, Paul B. Ecological impacts of termites. Termi Sustain Managem (201-216). Springer, Cham. 2018
13. Azizi-Shotorkhoft A, Mohammadabadi T, Motamedi H, Chaji M and Fazaeli H. Isolation and identification of termite gut symbiotic bacteria with lignocellulose-degrading potential and their effects on the nutritive value for ruminants of some by-products. Anim Feed Sci Technol. 2016;221:234-242.
14. Nasehi M, Torbatinejad NM, Zerehdaran S and Safaei AR. Effect of (Pleurotus florida) fungi on chemical composition and rumen degradability of wheat and barley straw. Iran J Appl Anim Sci. 2014;4(2):257-261.
15. Krause DO, Denman SE, Mackie RI, Morrison M, Rae AL, Attwood GT and McSweeney CS. Opportunities to improve fiber degradation in the rumen: microbiology ecology and genomics. FEMS microbiol rev. 2003;27(5):663-693.
16. Borji M. The survey possibility of straw polysaccharides and lignin degradation by isolated microbiota from termites (Doctoral dissertation PhD Thesis Tarbiat Modares University Tehran (Iran). 2003
17. Kato K, Kozaki S and Sakuranaga M. Degradation of lignin compounds by bacteria from termite guts. Biotechnol let. 1998;20(5):459-462.
18. Watanabe H and Tokuda G. Cellulolytic systems in insects Annu rev entomo.2010;55.
19. Nagaraju DK, Kalleshwaraswamy CM, Iyyanar D, Singh M, Jain RK, Kasturi N, Ranjith M, Mahadevaswamy HM and Asokan R. First interception of two wood feeding potential invasive Coptotermes termite species in India. Int J Tropi Insect Sci. 2020;1-10.
20. El-Sebay Y. A modified El-Sebay trap for subterranean termites fourth Arab. In Congress of Plant Protection Cairo Egypt. 1991;245-247.
21. Sarkar S, Kumawat PK, Bhatt RS, Sahoo A. Isolation and characterization of tanninolytic bacteria from sheep rumen contents: Assessment of tannin degrading, fibrolytic and feed digestibility potential. Small Rumin. Res. 2024; 19:107272.
22. Coico R. Gram staining Curr protoc immunol. 1997;23(1) A-3O

23. Agarwal N, Agarwal I, Kamra DN, Chaudhary LC. Diurnal variations in the activities of hydrolytic enzymes in different fractions of rumen contents of Murrah buffalo. J App Anim Res. 1;18(1):73-80.
24. Dye DW, Lelliott RA, Buchanan R and Gibbons N (1974) Genus II Xanthomonas Dowson 1939 187 Bergey's Manual of Determinative Bacteriology 89.
25. Sahu NP, Kamra DN and Paul SS. Effect of cellulose degrading bacteria isolated from wild and domestic ruminants on in vitro dry matter digestibility of feed and enzyme production. Asian austral J Anim Sci. 2004;17(2):199-202.
26. Gilna VV, Khaleel KM. Cellulase enzyme activity of Aspergillus fumigatus from mangrove soil on lignocellulosic substrate. Rec Resear Sci Technol. 2011;13;3(1).
27. Altschul SF, Madden TL, Schäffer AA, Zhang J, Zhang Z, Miller W, Lipman DJ. Gapped BLAST and PSI-BLAST: a new generation of protein database search programs. Nucleic Acids Res. 1997;25:3389–3402.
28. Tilley JMA and Terry DR. A two-stage technique for the in vitro digestion of forage crops. Grass Forage Sci. 1963;18(2):104-111.
29. Goering HK and Van Soest PJ. Forage fiber analyses: apparatus reagents procedures and some applications (No 379) Agricultural Research Service US Department of Agriculture. 1970.
30. Van Soest PJ, Robertson JB. A laboratory manual for Animal Science 612 Cornell University USA. 1985.
31. Zhou J, Duan J, Gao M, Wang Y, Wang X and Zhao K. Diversity roles and biotechnological applications of symbiotic microorganisms in the gut of termite. Curr microbial. 2019;76(6):755-761.
32. Afzal M, Farman M, Rasib KZ and Qureshi NA. Biocidal action of silver oak (Grevillea robusta) leaf extract on the termite Heterotermes indicola Wasmann (*Blattodea: Rhinotermitidae*). Int Biodeterior Biodegradation. 2019;139:1-10.
33. Butera G, Ferraro C, Alonzo G, Colazza S and Quatrini P. The gut microbiota of the wood-feeding termite Reticulitermes lucifugus. (*Isoptera; Rhinotermitidae*) Annal microbial. 2016;66(1) 253-260.
34. Femi-Ola TO and Oye bamiji BA. Molecular characterization and cellulolytic activities of bacterial isolates from the hindgut of wood-feeding termites *amitermes evuncifersilvestri*. Adv Microbiol. 2019;1-10.
35. Tsegaye B, Balomajumder C and Roy P. Isolation and characterization of novel lignolytic cellulolytic and hemicellulolytic bacteria from wood-feeding termite Cryptotermes brevis Int Microbiol. 2019;22(1):29-39.
36. Nelson K, Muge E and Wamalwa B. Cellulolytic Bacillus species isolated from the gut of the desert locust Schistocerca gregaria Scientific African pe00665. 2021.
37. Holt J, Krieg N, Sneath P, Staley J and Williams S. Bergey's manual of systematic bacteriology 9rd edition The Williams and Wilkins Co Baltimore. 1994.
38. Kuhnigk T and König H. Degradation of dimeric lignin model compounds by aerobic bacteria isolated from the hindgut of xylophagous termites. J Basic Microbiol. 1997;37(3):205-211.
39. Wenzel M, Schönig I, Berchtold M, Kämpfer P and König H. Aerobic and facultatively anaerobic cellulolytic bacteria from the gut of the termite *Zootermopsis angusticollis*. J app microbial. 2002;92(1):32-40.

40. Jalali M. Isolation and Identification of Gut Symbiont Bacteria in the Termite Anacanthotermes Vagans Hagen (Isopetra: Hodotermitidae) and Effects of Two Biopesticide Compound on the Termite MSc Thesis Shahid Chamran University of Ahvaz Iran. 2014.

41. Tokuda G, Mikaelyan A, Fukui C, Matsuura Y, Watanabe H, Fujishima M and Brune A. Fiber-associated spirochetes are major agents of hemicellulose degradation in the hindgut of wood-feeding higher termites PNAS 115(51) E11996-E12004. 2018.

42. Chantarasiri A. Aquatic Bacillus cereus JD0404 isolated from the muddy sediments of mangrove swamps in Thailand and characterization of its cellulolytic activity. Egypt J Aquat Res. 2015;41(3):257-264.

43. Johnsen HR and Krause K. Cellulase activity screening using pure carboxymethylcellulose: application to soluble cellulolytic samples and to plant tissue prints. Int J Mol Sci. 2014;15(1):830-838.

44. Gupta P, Samant K and Sahu A. Isolation of cellulose-degrading bacteria and determination of their cellulolytic potential. Int j microbial. 2012.

45. Liu B, Lin JD, Song SL and Wu DL. The Microbes Cultured and Their Function in the Gut of Termites. Bio Dis Sci. 2012.

46. Purwadari T, Ketaren PP, Sinurat AP, and Sutikno I. Identification and evaluation of fiber hydrolytic enzymes in the extract of termites (Glyptotermes montanus) for poultry feed application. Indo Journ Agri Sci. 2003;4,40-47.

47. Zhang M, Liu N, Qian C, Wang Q, Long Y, Huang Y, Zhou Z and Yan X. Phylogenetic and functional analysis of gut microbiota of a fungus-growing higher termite: Bacteroidetes from higher termites are a rich source of β-glucosidase genes. Micro ecol. 2014;68(2):416-425.

48. Katiyar P, Srivastava SK and Tyagi VK. Measurement of cellulolytic potential of cellulase producing bacteria. Annu Res Rev Biol. 2018;1-9.

49. Veivers PC, O'Brien RW and Slaytor M. Role of bacteria in maintaining the redox potential in the hindgut of termites and preventing entry of foreign bacteria, J Insect Physiol. 1982;28(11):947-951.

50. Mattéotti C, Bauwens J, Brasseur C, Tarayre C, Thonart P, Destain J, Francis F, Haubruge E De Pauw E, Portetelle D and Vandenbol M. Identification and characterization of a new xylanase from Gram-positive bacteria isolated from termite gut (*Reticulitermes santonensis*). Protein Expr and Pur. 2012;83(2):117-127.

51. Mulyani PD, Hamid RM, Janatunaim RZ, Purwestri YA. Amylolytic ability of bacteria isolated from termite (Coptotermes sp.) gut. Indo Journ Biotechnol. 2018;23(1):14-20.

52. Sakolvaree J and Deevong P. Isolation and Characterization of Cellulase Producing Bacteria from the Gut of a Higher Termite *Termes propinquus*. 2016.

53. Tarayre C, Brognaux A, Bauwens J, Brasseur C, Mattéotti C, Millet C, Destain J, Vandenbol M, Portetelle D, De Pauw E and Eric H. Isolation of amylolytic xylanolytic and cellulolytic microorganisms extracted from the gut of the termite Reticulitermes santonensis by means of a micro-aerobic atmosphere. World J Microbiol Biotechnol. 2014;30(5):1655-1660.

54. Breznak JA and Brune A. Role of microorganisms in the digestion of lignocellulose by termites. Annu rev entomol. 1994;39:453-487.

55. Ramin M, Alimon AR, Sijam K and Abdullah N. Filter paper degradation by bacteria isolated from local termite gut Res J Microbiol. 2008;3(8):565-568.

56. Shinde VS, Shinde RM, Agrawal T and Kotasthane AS. Extracellular cellulase activity of termite gut bacteria isolated from different geographical locations of chhattisgarh (CG). India Plant Archiv. 2017;17(1):601-607.
57. Yang H, Schmitt-Wagner D, Stingl U and Brune A. Niche heterogeneity determines bacterial community structure in the termite gut (*Reticulitermes santonensis*). Environ Microbiol. 2005;7(7):916-932.
58. Chew YM, Lye S, Salleh MM and Yahya A. 16S rRNA metagenomic analysis of the symbiotic community structures of bacteria in foregut midgut and hindgut of the wood-feeding termite. Bulbitermes sp Symbiosis. 2018;76(2):187-197.
59. Köhler T, Dietrich C, Scheffrahn RH and Brune A. High-resolution analysis of gut environment and bacterial microbiota reveals functional compartmentation of the gut in wood-feeding higher termites (*Nasutitermes spp*). AEM. 2012;78(13):4691-4701.
60. Miyata R, Noda N, Tamaki H, Kinjyo K, Aoyagi H, Uchiyama H and Tanaka H. Influence of feed components on symbiotic bacterial community structure in the gut of the wood-feeding higher termite *Nasutitermes takasagoensis*. Biosci biotechnol biochem. 2007;71(5):1244-1251.
61. Okano K, Ohkoshi N, Nishiyama A, Usagawa T and Kitagawa M. Improving the nutritive value of madake bamboo Phyllostachys bambusoides for ruminants by culturing with the white-rot fungus *Ceriporiopsis subvermispora*. Anim Feed Sci Technol. 2009;152(3-4):278-285.
62. Azizi A, Sharifi A and Fazaeli H. Effect of one produced slow-release urea component on gas production fermentation nutrient disappearance and activity of microbial enzymes using rumen liquor of sheep. Anim Sci J. 2019;32(122):279-290.
63. Adams L, Boopathy R. Isolation and characterization of enteric bacteria from the hindgut of Formosan termite. Bioresour Technol. 2005;96:1592–8.

Table 1 Morphological and biochemical characteristics of different isolates

Isolates	Gram Stain	Shape	Gas Production Test	Anaerobiosis Test	Catalase Test	Motility Test	H_2S Production Test	Indole Production Test	Methyl Red Test
**TM1**	+	Rod	-	+	-	Motile	+	-	-
**TM2**	-	Rod	+	+	-	Motile	+	-	+
**TM3**	-	Rod	+	+	-	Motile	+	-	+
**TM4**	-	Rod	+	+	-	Motile	+	-	+
**TM5**	-	Rod	-	+	-	Motile	+	-	-
**TM6**	+	Rod	-	+	-	Non motile	+	-	+
**TM7**	+	Rod	-	+	-	Motile	+	-	-
**TM8**	+	Rod	+	-	+	Non motile	+	-	+
**TM9**	+	Rod	-	-	+	Motile	+	-	+
**TM10**	+	Rod	-	+	-	Motile	+	-	+
**TM11**	+	Rod	+	+	-	Non motile	+	-	-
**TM12**	+	Rod	-	+	-	Motile	-	-	+
**TM13**	+	Rod	-	+	-	Motile	+	-	+
**TM14**	-	Rod	+	-	+	Non motile	+	-	+
**TM15**	-	Rod	+	+	-	Non motile	+	-	+
**TM16**	+	Cocci	+	+	-	Non motile	+	-	+
**TM17**	+	Rod	-	+	-	Motile	+	-	+
**TM18**	+	Cocci	+	+	-	Non motile	+	-	+
**TM19**	+	Rod	-	+	-	Non motile	+	-	+
**TM20**	+	Rod	-	+	-	Non motile	+	-	+
**TM21**	-	Rod	+	+	-	Motile	+	-	+
**TM22**	-	Rod	-	-	+	Non motile	+	-	+
**TM23**	+	Rod	+	+	-	Non motile	+	-	+
**TM24**	+	Rod	+	+	-	Motile	+	-	-

(-) Non-utilizers, (+) Utilizers

Table 2 Utilization of various carbohydrates by different isolates

Isolates	1	2	3	4	5	6	7	8	9	10	11	12	13
**TM1**	+	+	+	+	-	-	+	+	+	+	+	+	+
**TM2**	+	+	+	+	+	+	+	+	+	+	+	+	+
**TM3**	+	+	+	+	+	+	+	+	+	+	+	+	-
**TM4**	+	+	+	+	+	+	+	+	+	+	+	+	-
**TM5**	+	+	+	+	+	+	+	+	+	+	+	+	-
**TM6**	+	-	+	+	+	+	+	+	+	+	+	+	-
**TM7**	+	+	+	+	-	-	+	+	+	+	+	+	-
**TM8**	+	+	+	+	+	+	+	+	+	+	+	+	-
**TM9**	+	+	+	+	+	+	+	+	+	+	+	+	-
**TM10**	-	+	+	+	+	-	+	+	+	+	+	+	+
**TM11**	+	+	+	+	+	+	+	+	+	+	+	+	+
**TM12**	+	-	+	+	+	+	+	+	+	+	+	+	+
**TM13**	-	+	+	+	+	+	+	-	+	+	+	+	+
**TM14**	+	+	+	+	+	+	+	-	+	+	+	+	+
**TM15**	-	+	+	+	-	-	+	-	+	+	+	+	+
**TM16**	+	+	+	+	+	+	+	+	+	+	+	+	-
**TM17**	+	+	+	+	+	+	+	+	+	+	+	+	+
**TM18**	-	+	-	+	+	+	+	-	+	+	+	+	-
**TM19**	+	+	+	+	+	+	+	+	+	+	+	+	+
**TM20**	-	+	+	+	+	-	+	-	+	+	+	+	-
**TM21**	-	+	+	+	+	+	+	-	+	+	+	+	+
**TM22**	+	+	+	+	+	+	+	+	+	+	+	+	-
**TM23**	+	+	+	+	+	+	+	+	+	+	+	+	+
**TM24**	+	+	-	+	+	+	+	-	+	+	+	+	+

(-) Non-utilizers, (+) Utilizers

1. Galactose, 2. Mannose, 3. Sucrose, 4. Glucose, 5. Xylose, 6. Xylan, 7. Carboxy-methyl cellulose, 8. Fructose, 9. Lactose, 10. Arabinose, 11. Starch, 12. Maltose, 13. Ribose

Table 3 Fibrolytic enzyme activities of bacterial isolates

**Isolates**	**1**	**2**	**3**	**4**	**5**	**6**
**TM1**	5.21^{abcd}	7.08^{efghi}	8.58^{bcdef}	0.009^{a}	5.17^{cdef}	1.72^{abcd}
**TM2**	5.00^{ab}	6.53^{defghi}	8.03^{abcde}	0.071^{d}	3.35^{abc}	8.44^{kl}
**TM3**	5.69^{abcdef}	5.87^{bcdef}	8.93^{cdef}	0.015^{ab}	2.04^{a}	3.11^{cdefg}
**TM4**	5.51^{abcdef}	6.16 ^{cdef}	8.45^{bcdef}	0.026^{abc}	4.16a^{bcd}	2.88^{bcdef}
**TM5**	4.83^{a}	5.57^{bcde}	12.05^{g}	0.041^{abcd}	5.32c^{defg}	12.00^{m}
**TM6**	7.60^{h}	8.40^{j}	8.67^{bcdef}	0.059^{cd}	7.34f^{ghi}	5.55^{hij}
**TM7**	5.97^{bcdefg}	7.08^{efghi}	8.58^{bcdef}	0.009^{a}	5.17^{cdef}	1.72^{abcd}
**TM8**	6.31^{efg}	7.64^{ghij}	8.35^{bcdef}	0.072^{d}	5.68^{cdefgh}	3.44^{defg}
**TM9**	6.83^{gh}	7.49^{fghij}	9.64^{ef}	0.031^{abcd}	4.36^{abcd}	1.11^{abc}
**TM10**	6.36^{fg}	8.14^{ij}	8.93^{cdef}	0.055^{bcd}	6.03^{defgh}	0.83^{ab}
**TM11**	5.69^{abcdef}	6.22^{cdefg}	8.41^{bcdef}	0.040^{abcd}	2.19^{ab}	2.88^{bcdef}
**TM12**	5.90^{bcdefg}	7.69^{hij}	7.81^{abcd}	0.371^{fg}	10.73^{jk}	5.11^{ghij}
**TM13**	5.30^{abcde}	5.37^{abcde}	9.42^{def}	0.396^{gh}	5.02^{cdef}	2.11^{abcde}
**TM14**	5.92^{cdefg}	6.38^{cdefgh}	8.80^{bcdef}	0.384^{fgh}	9.67^{ij}	10.16^{lm}
**TM15**	5.37^{bcdef}	6.11^{cdef}	7.12^{ab}	0.407^{gh}	8.10^{hi}	0.66^{a}
**TM16**	5.25^{abcd}	5.12^{abc}	8.76^{bcdef}	0.501^{k}	2.34^{ab}	5.83^{ij}
**TM17**	6.21^{defg}	5.53^{bcde}	7.67^{abc}	0.454^{ij}	11.23^{jk}	3.66^{defgh}
**TM18**	5.63^{abcdef}	5.55^{bcde}	7.44^{abc}	0.541^{l}	3.35^{abc}	5.00f^{ghij}
**TM19**	5.65^{abcdef}	4.24^{a}	6.41^{a}	0.465^{jk}	3.76a^{bcd}	3.92^{efghi}
**TM20**	5.17^{abc}	4.79^{ab}	7.52^{abc}	0.423^{hi}	7.85^{ghi}	0.55^{a}
**TM21**	5.32^{abcde}	5.20^{abcd}	7.24^{abc}	0.349^{f}	4.69^{bcde}	7.1^{jk}
**TM22**	6.28^{efg}	6.38^{cdefgh}	9.92^{f}	0.124^{e}	12.42^{k}	8.27^{kl}
**TM23**	7.24^{h}	6.18^{cdef}	7.95^{abcde}	0.051^{abcd}	6.36^{defgh}	6.77^{jk}
**TM24**	5.37^{abcdef}	6.64^{defgh}	8.40^{bcdef}	0.073^{d}	3.08^{abc}	10.22^{lm}
**SEM**	0.08398	0.12864	0.13664	0.0222	0.33731	0.37590
**pValue**	0.000	0.000	0.000	0.000	0.000	0.000

1. Endoglucanase Activity (µmol glucose/h/ml or units/ml), 2. Exoglucanase Activity (µmol glucose/h/ml or units/ml), 3. FPase Activity (µmol glucose/h/ml or units/ml), 4. β-Glucosidase Activity (µmol p-Nitrophenol/min/ml or units/ml), 5. α-Amylase Activity (µmol glucose/h/ml or units/ml), 6. Xylanase Activity (µmol xylose/h/ml or units/ml), $^{a-m}$ means bearing superscript differs significantly in a column ($P < 0.05$).

Table 4 Filter paper degradation potential of different isolates

Isolates	24h	48h	72h	96h	5D	6D	7D	8D
**TM1**	+	++	++	+++	++++	+++++	++++++	CD
**TM2**	+	+	++	+++	++++	+++++	++++++	CD
**TM3**	+	++	++	+++	++++	+++++	++++++	CD
**TM4**	+	++	++	+++	++++	+++++	++++++	CD
**TM5**	++	+++	++++	+++++	+++++	++++++	CD	
**TM6**	+	++	+++	++++	+++++	+++++	++++++	CD
**TM7**	+	++	++	+++	++++	+++++	++++++	CD
**TM8**	+	+	++	+++	++++	+++++	++++++	CD
**TM9**	+	++	+++	++++	+++++	++++++	CD	
**TM10**	+	++	+++	++++	+++++	+++++	++++++	CD
**TM11**	+	++	++	+++	++++	+++++	++++++	CD
**TM12**	+	+	++	+++	++++	+++++	++++++	CD
**TM13**	+	++	+++	++++	+++++	++++++	CD	
**TM14**	+	+	++	+++	++++	+++++	++++++	CD
**TM15**	+	+	++	+++	++++	+++++	++++++	CD
**TM16**	+	+	++	+++	++++	+++++	++++++	CD
**TM17**	+	++	++	+++	++++	+++++	++++++	CD
**TM18**	+	++	++	+++	++++	+++++	++++++	CD
**TM19**	+	+	++	+++	++++	+++++	++++++	CD
**TM20**	+	+	++	+++	++++	+++++	++++++	CD
**TM21**	+	+	+++	+++	++++	+++++	++++++	CD
**TM22**	+	++	++	+++	++++	+++++	++++++	CD
**TM23**	+	+	++	+++	++++	+++++	++++++	CD
**TM24**	+	++	++	+++	++++	+++++	++++++	CD

*CD= Complete Degradation, h=Hours, D= Days

Table 5 16S rDNA sequencing based identification

Isolates	Nearest Valid Taxon	% similarity	Sequence base pairs	Phylum	Family	Gene Bank Accession
TM1	*Bacillus megaterium strain IAM 13418*	99	1473	Firmicutes	Bacillaceae	MT356127
TM2	*Citrobacter freundii strain JCM 1657*	98	1417	Proteobacteria	Enterobacteriaceae	MT356128
TM3	*Citrobacter farmeri strain CDC 2991-81*	99	1472	Proteobacteria	Enterobacteriaceae	MT356129
TM4	*Citrobacter freundii strain JCM 1657*	98	1427	Proteobacteria	Enterobacteriaceae	MT356130
TM5	*Bacillus flexus strain IFO15715*	99	1530	Firmicutes	Bacillaceae	MT356131
TM6	*Pilibacter termitis strain TI-1*	98	1413	Firmicutes	Enterococcaceae	MT356132
TM7	*Bacillus megaterium strain IAM*	99	1482	Firmicutes	Bacillaceae	MT356133
TM8	*Bacillus licheniformis strainATCC14580*	99	1485	Firmicutes	Bacillaceae	MT356134
TM9	*Bacillus cereus strain ATCC 14579*	98	1524	Firmicutes	Bacillaceae	MT356135
TM10	*Clostridium termitidis*	99	1446	Firmicutes	Hungateiclostridiaceae	MT356136
TM11	*Clostridium saccharobutylicum strain P262*	100	1353	Firmicutes	Clostridiaceae	MT356137
TM12	*Clostridium indolis strain 7*	97	1414	Firmicutes	Lachnospiraceae	MT356138
TM13	*Clostridium termitidis*	98	1446	Firmicutes	Hungateiclostridiaceae	MT356139
TM14	*Bacillus oleronius strain DSM 9356*	98	1477	Firmicutes	Bacillaceae	MT356140
TM15	*Clostridium cellulovorans 743B*	96	1447	Firmicutes	Clostridiaceae	MT356141
TM16	*Lactococcus nasutitermitis strain M19*	97	1446	Firmicutes	Streptococaceae	MT356142
TM17	*Clostridium sporogenes strain JCM 1416*	90	1535	Firmicutes	Clostridiaceae	MT356143
TM18	*Lactococcus nasutitermitis strain M19*	99	1440	Firmicutes	Streptococaceae	MT356144
TM19	*Bacillus circulans strain ATCC 4513*	98	1482	Firmicutes	Bacillaceae	MT356145
TM20	*Bacillus circulans strain ATCC 4513*	98	1482	Firmicutes	Bacillaceae	MT356146
TM21	*Ruminiclostridium papyrosolvens DSM 2782*	98	1382	Firmicutes	Hungateiclostridiaceae	MT356147
TM22	*Brevibacillus brevis strain DSM 30*	96	1447	Firmicutes	Paenibacillaceae	MT356148
TM23	*Clostridium puniceum strain BL 70/20*	96	1481	Firmicutes	Clostridiaceae	MT356149
TM24	*Clostridium sporogenes strain JCM 1416*	99	1489	Firmicutes	Clostridiaceae	MT356150

Table 6 Fermentation attributes total short chain fatty acid production

Isolates	Acetate(C2) mM	Propionate (C3) mM	Iso-Butyrate (C4i) mM	Butyrate (C4) mM	Iso-Valerate (C5i) mM	Valerate (C5) mM	Total SCFAs mM	BcFA	NGR	C3:(C2+C4)	C2:C3
**TM1**	56.74^{i}	14.26^{f}	1.71^{bcd}	7.34^{g}	0.96^{def}	1.18^{g}	82.19^{hij}	3.85^{cde}	4.16^{ab}	0.22^{defg}	3.98^{abc}
**TM2**	70.72^{k}	14.09^{ef}	2.83^{f}	6.93^{defg}	1.15^{fg}	1.33^{g}	97.05^{k}	5.32^{fg}	4.64^{bc}	0.18^{b}	5.03^{cd}
**TM3**	60.88^{j}	14.10^{ef}	1.96^{cde}	7.30^{fg}	0.92^{def}	0.98^{f}	86.15^{j}	3.87^{cde}	4.42^{abc}	0.21^{cd}	4.32^{abc}
**TM4**	56.96^{i}	13.96^{ef}	1.86^{bcd}	7.16^{fg}	1.01^{efg}	1.27^{g}	82.23^{hij}	4.14^{e}	4.18^{ab}	0.22^{def}	4.09^{abc}
**TM5**	61.35^{j}	6.93^{a}	2.82^{f}	3.08^{ab}	0.96^{def}	0.39^{b}	75.54^{efg}	4.17^{e}	6.55^{e}	0.11^{a}	8.97^{f}
**TM6**	83.59^{l}	17.87^{g}	2.77^{f}	10.34^{h}	1.56^{h}	1.61^{h}	117.73^{l}	5.93^{g}	4.63^{bc}	0.19^{bc}	4.68^{bcd}
**TM7**	44.82^{b}	10.61^{b}	1.37^{bcd}	5.92^{def}	0.73^{bcd}	0.74^{cde}	64.19^{b}	2.84^{bc}	4.43^{abc}	0.21^{cd}	4.23^{abc}
**TM8**	51.47^{efg}	13.43^{def}	1.54^{bcd}	7.15^{fg}	0.93^{def}	0.96^{f}	75.50^{efg}	3.44^{bcde}	4.11^{ab}	0.23^{defh}	3.84^{ab}
**TM9**	49.26^{de}	13.43^{def}	1.42^{bcd}	7.03^{defg}	0.80^{bcde}	0.97^{f}	72.91^{cdef}	3.19^{bcde}	4.01^{ab}	0.24^{efghi}	3.67^{ab}
**TM10**	44.82^{b}	10.61^{b}	1.37^{bcd}	5.92^{def}	0.73^{bcd}	0.74^{cde}	64.19^{b}	2.84^{bc}	4.43^{abc}	0.21^{cd}	4.23^{abc}
**TM11**	55.17^{hi}	6.92^{a}	2.60^{e}	3.67^{bc}	1.23^{g}	0.56^{c}	70.15^{bcde}	4.39^{ef}	5.96^{de}	0.12^{a}	8.05^{e}
**TM12**	63.17^{j}	11.29^{bc}	2.05^{de}	4.46^{c}	1.13^{fg}	0.90^{ef}	83.00^{ij}	4.08^{de}	4.97^{c}	0.17^{b}	5.61^{d}
**TM13**	51.44^{efg}	13.14^{cdef}	1.53^{bcd}	6.74^{defg}	0.82^{bcde}	0.97^{f}	74.65^{defg}	3.32^{bcde}	4.15^{ab}	0.23^{defg}	3.92^{ab}
**TM14**	44.26^{b}	12.10^{bcde}	1.37^{bcd}	6.14^{defg}	0.71^{bcd}	0.79^{def}	65.39^{b}	2.88^{bcd}	3.98^{ab}	0.24^{fghi}	3.67^{ab}
**TM15**	50.37^{ef}	14.85^{f}	1.44^{bcd}	7.41^{g}	0.90^{cdef}	0.98^{f}	75.96^{efgh}	3.32^{bcde}	3.78^{a}	0.26^{i}	3.40^{a}
**TM16**	55.47^{hi}	14.10^{ef}	1.54^{bcd}	7.15^{fg}	0.93^{def}	0.96^{f}	80.16^{ghij}	3.44^{bcde}	4.20^{abc}	0.23^{defg}	3.96^{abc}
**TM17**	45.67^{bc}	13.23^{cdef}	1.30^{abcd}	6.55^{defg}	0.71^{bcd}	0.75^{cde}	68.20^{bcd}	2.76^{bc}	3.86^{ab}	0.25^{hi}	3.46^{a}
**TM18**	48.24^{cde}	11.22^{b}	1.17^{abc}	5.74^{de}	0.65^{bc}	0.62^{cd}	67.63^{bc}	2.44^{b}	4.55^{abc}	0.21^{cd}	4.30^{abc}
**TM19**	49.25^{de}	13.89^{ef}	1.20^{abc}	6.65^{defg}	0.71^{bcd}	0.78^{def}	72.48^{cdef}	2.70^{bc}	3.93^{ab}	0.25^{ghi}	3.55^{a}
**TM20**	49.96^{def}	13.34^{def}	1.10^{ab}	6.91^{defg}	0.73^{bcd}	0.81^{def}	72.85^{cdef}	2.64^{bc}	4.16^{ab}	0.23^{deghi}	3.75^{ab}
**TM21**	50.66^{efg}	14.02^{ef}	1.18^{abc}	6.91^{defg}	0.78^{bcde}	0.76^{cde}	74.30^{defg}	2.71^{bc}	4.02^{ab}	0.24^{fghi}	3.62^{ab}
**TM22**	46.86^{bcd}	11.75^{bcd}	1.20^{abc}	5.68^{d}	0.63^{b}	0.65^{cd}	66.77^{bc}	2.48^{b}	4.27^{abc}	0.22^{defg}	3.99^{abc}
**TM23**	53.80^{ghi}	14.54^{f}	1.21^{abc}	7.09^{efg}	0.75^{bcde}	0.79^{def}	78.19^{fghi}	2.76^{bc}	4.09^{ab}	0.24^{efghi}	3.70^{ab}
**TM24**	53.12^{fgh}	13.22^{cdef}	1.29^{abcd}	6.93^{defg}	0.70^{bcd}	0.81^{def}	76.07^{efgh}	2.79b^{c}	4.36^{abc}	0.22^{def}	4.02^{abc}
**Control**	24.63^{a}	5.66^{a}	0.58^{a}	2.04^{a}	0.15^{a}	0.19^{a}	33.26^{a}	0.92^{a}	4.50^{abc}	0.21^{cde}	4.35^{abc}
**SEM**	1.21	0.318	0.071	0.05	0.03	0.03	1.64	0.12	0.07	0.03	0.155

Branch − chain fatty acids (BcFA) = valerate + iso − valerate + iso − butyrate (C5 + C5i + C4i).NGR Non - glucogenic: Glucogenicratio- = [(Acetate + 2 × Butyrate + BcFA ÷ Propionate + BcFA-] or [C2 + 2C4 + C5 + C5i + C4i- ÷ C3 + C5 + C5i + C4i-].a,b,c Means bearing different superscript differ significantly ($p < .05$). SCFA, short-chain fatty acids; and SEM, standard error of mean.

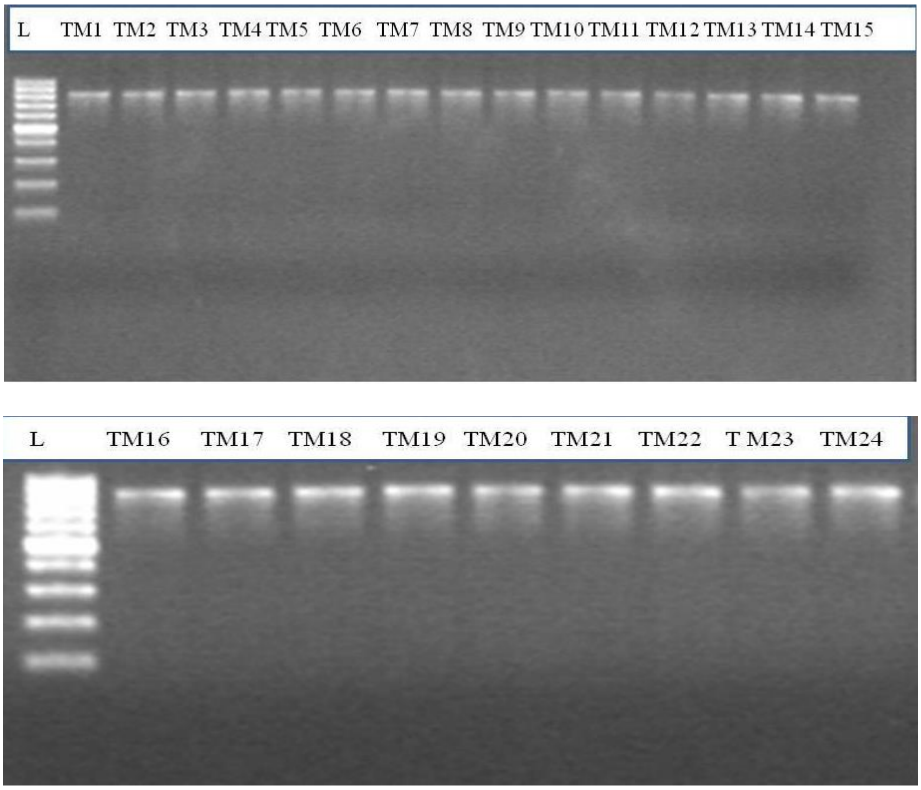

Figure 1.16s rDNA amplification of isolates (TM1-TM24) of size ~1500 bp and L is 1Kb Ladder

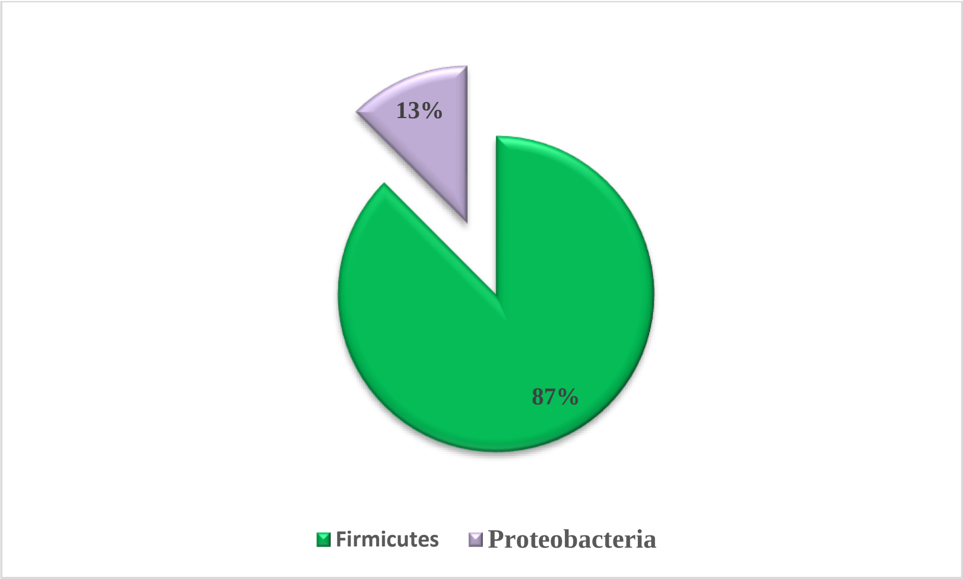

Figure 2. Main phyla abundance of fibrolytic bacteria isolated from gut of wood eating lower termite (*Coptotermes heimi*)

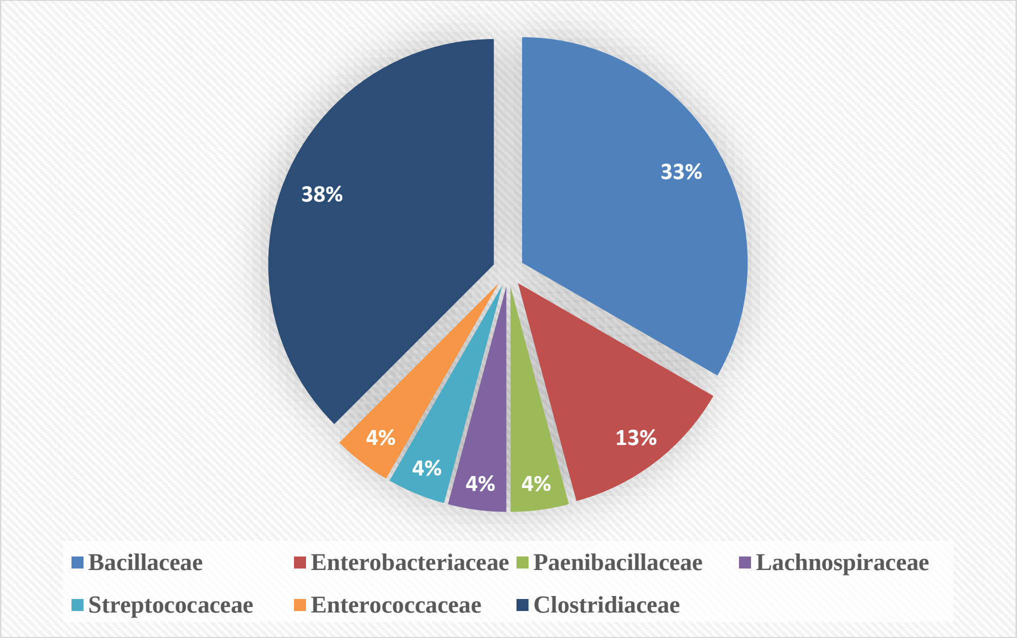

Figure3. Distribution of fibrolytic bacteria identified in the gut of wood eating lower termite (*Coptotermes heimi*) at family level

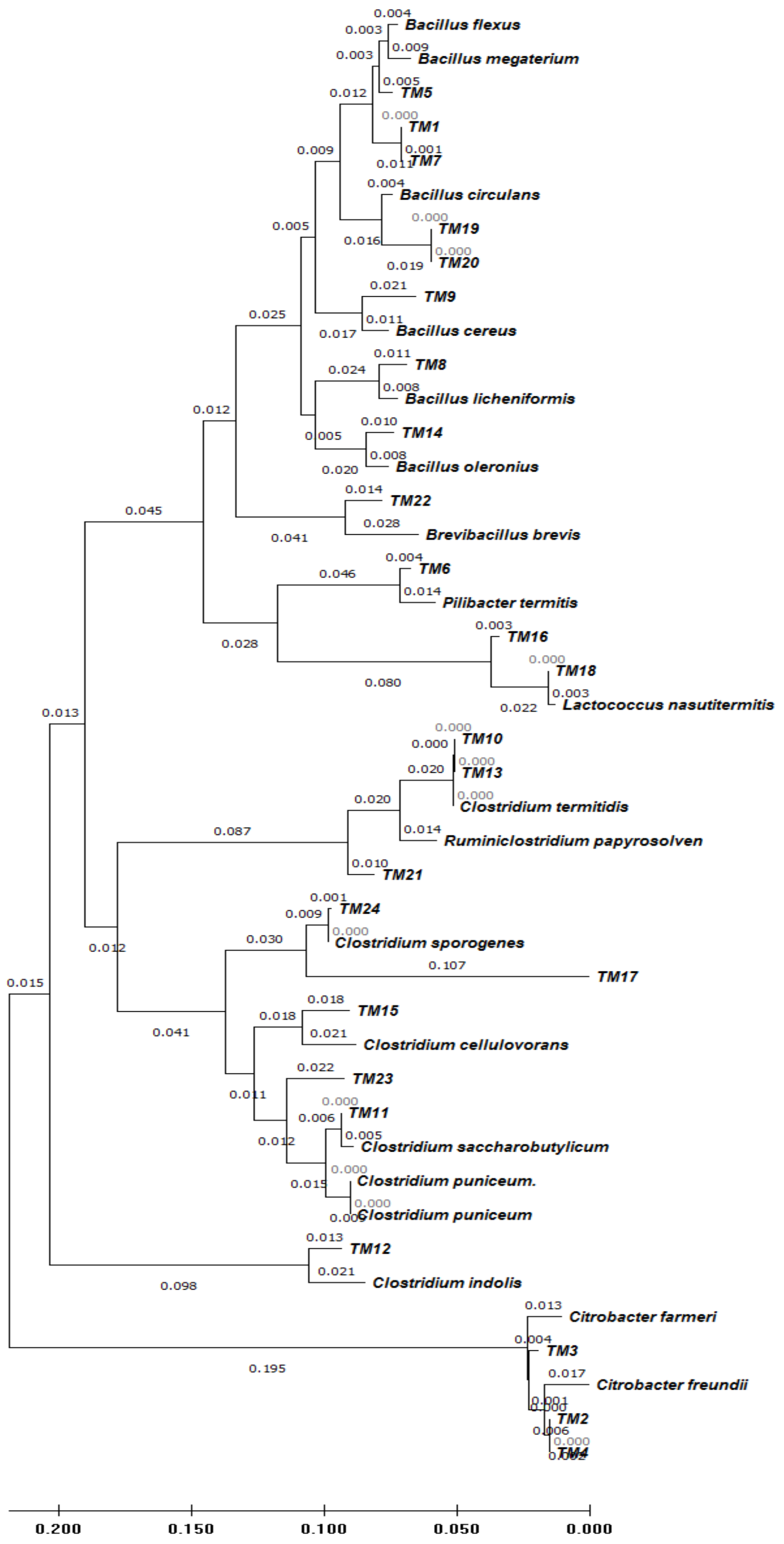

Figure 4 Phylogentic tree based on the 16S rRNA partial sequences of the isolates. Scale bar=1 inferred nucleotide substitution per 50 nucleotides.

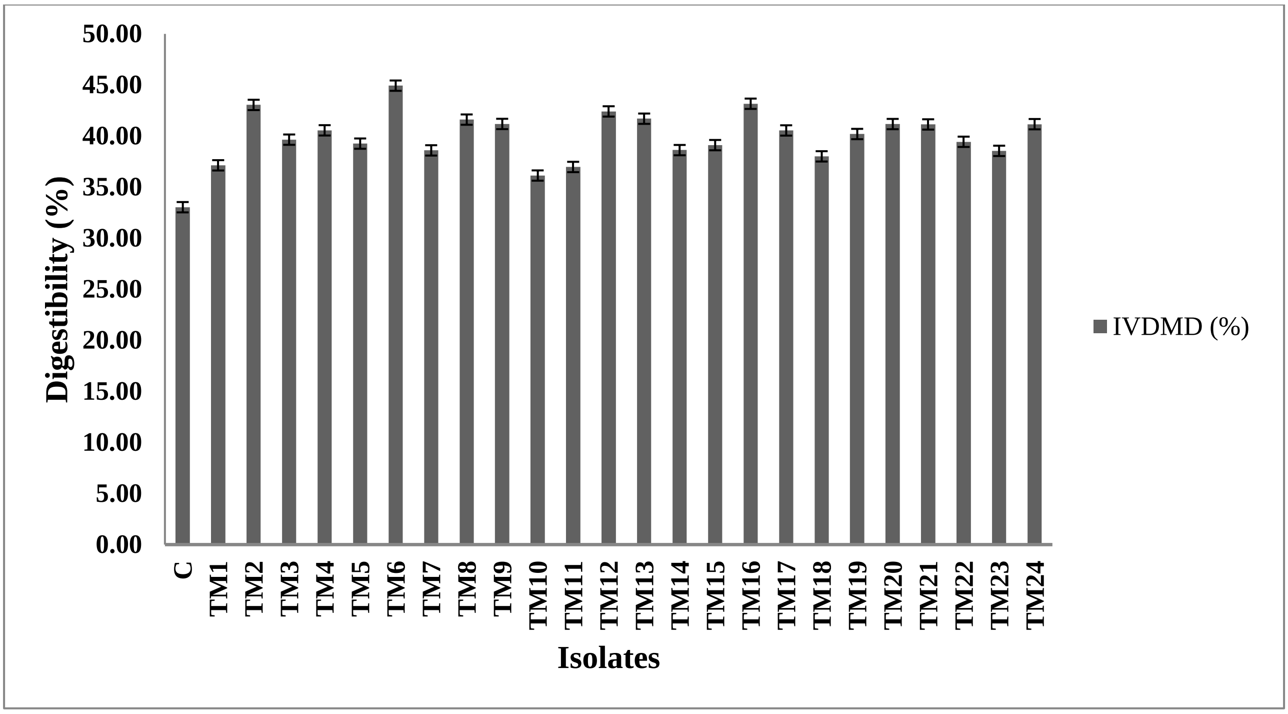

Figure5 Effect of inclusion of bacterial isolates on *in vitro* dry matter (IVDMD) digestibility at 48h post incubation.

Click here to access/download
Supporting Information
S1_raw_images.docx

Lower termite (*Coptotermes heimi*) gut fibrolytic bacterial consortium: Isolation, phylogenetic characterization, fibre degradation potential~~, phylogenetic dynamics~~ and *in vitro* digestibility

Pankaj Kumar Kumawat[1,3], Srobana Sarkar[1*], Satish Kumar[3,4], Artabandhu Sahoo[1,2*]

[1]ICAR- Central Sheep and Wool Research Institute, Avikanagar, Rajasthan

[2] ICAR- National Research Centre on Camel, Bikaner, Rajasthan

[3]Mewar University, Rajasthan

[*]**Corresponding authors:** Artabandhu Sahoo, Email address: sahooarta1@gmail.com; Srobana Sarkar, Email address: sarkarsrobana@gmail.com

Phone No.: 01437- 220162, Fax: 01437- 220163

Pankaj Kumar Kumawat: PhD scholar, Mewar University, Rajasthan and Senior Research Fellow, ICAR-Central Sheep and Wool Research Institute, Avikanagar, Rajasthan.

Srobana Sarkar: Scientist, ICAR-Central Sheep and Wool Research Institute, Avikanagar, Rajasthan.

A. Sahoo: Principal Scientist &Director, ICAR- National Research Centre on Camel, Bikaner, Rajasthan.

Satish Kumar: Principal, Mata Basanti Devi School of Biosciences & Biotechnology, Agra.

[4]**Present address**- Mata Basanti Devi School of Biosciences & Biotechnology, Agra

Abstract

Lower termites produce wide array of fibrolytic enzymes and serves as prospective microbial enzymes source for enhancing biodegradability of recalcitrant ligno-cellulosic~~poor quality~~ fibrous feeds. The present study was aimed to isolate and characterize anaerobic fibrolytic bacteria from gut of termite *Coptotermes heimi* ~~for~~ for screening promising isolates to improve fiber digestibility in ruminants. A total of 141 isolates were obtained from 97 termite gut samples, and 24 selected isolates (TM1 to TM24) isolates were characterized as obligatory anaerobes and catalase negative except, TM8, TM9, TM14 and TM22. Overall fibrolytic enzyme activity was highest in isolate TM23, TM6 and TM22~~ than other isolates~~. The highest FPase activity was observed in isolate TM5 (12.05 U/ml) while, ~~the~~ it was lowest in TM19 (6.41 U/ml). The ~~Phylogenetic~~ phylogenetic analysis depicted four major families i.e., *Clostridiales*, *Bacillales*, *Lactobacillales* and *Enterobacterales* under phyla *Firmicutes* and *Proteobacteria*. The ~~*In*~~ *in vitro* dry matter digestibility of the substrate was increased by 9.4 to 36.0% with the inoculation of isolated bacterial strains. Among the screened isolates, TM6 ~~showed the highest~~exhibited highest ability to improve *in vitro* dry matter digestibility. The findings of the present study revealed that fibrolytic bacteria isolated from the termite gut can be used for commercial enzyme production or in rumen biotechnological application for enhancing fibrous feed utilization in ruminants.~~as a biological treatment for enhancing the utilization of fibrous feed in ruminants.~~

Keywords Lower termites, *Coptotermes heimi*, gut, 16S rRNA, fibrolytic enzymes, digestibility

Introduction

Termites acts as "ecosystem engineers" because they help in efficient degradation of complex plant biomass into resourceful bioenergy in comparison to other invertebrates [1,2]. Their potential to degrade fibrous polysaccharide depends on the dual enzymatic machinery of termite and its gut microbial symbionts, which makes ~~the termites~~them -an ideal source of microbial enzymes [3, 4]. Termites have the ability to degrade around 65 to 87% hemicelluloses and 74 to 99% cellulose ~~from lignocellulosics~~ [5]. ~~Termites~~ They are intricately grouped into higher and lower termites [6],. the ~~Higher~~ higher termites harbour~~s~~ only prokaryotes and lacks protists [7,8,5], while the, lower termites contain~~s~~ both protists and bacteria in their gut [9]. The microbes ~~present in~~present in termite gut work~~s~~ in consortium and help~~s~~ in ~~break~~breaking down ~~of~~ complex carbohydrates [10,11]. However, the abundance of microbes inhabiting in the gut of termites not only depends on their hierarchy but also on their geographical location and available vegetation [12].

Utilization of agro-industrial by-products and poor-quality fibrous feeds in animal nutrition plays a pivotal role in sustainable agriculture. Effect of several treatments including physical, chemical and biological have been studied for enhancing utility of poor-quality feed resources in ruminants [13,14]. ~~Adding exogenous fibrolytic enzymes helps~~ In ruminants, exogenous fibrolytic enzymes aid in increasing the digestibility of fiber~~ to improve fibre digestibility in ruminants~~ [15]. Earlier studies reported that bacteria isolated from termite gut have ~~potential~~ the ability to disintegrate lignin barrier and digest carbohydrate polymers [13,16-18]. Additionally, because the environments in the rumen and termites' guts are similar in terms of anaerobiosis, reductive

acetogenesis, methanogenesis, and production of volatile fatty acids, the bacteria isolated from termite guts are better suited as microbial feed additives to enhance the utilization of recalcitrant ligno-cellulosic fibrous crop residues. ~~Furthermore, resemblance between rumen and termite gut environment such as anaerobiosis, reductive acetogenesis methanogenesis and volatile fatty acid production, makes bacteria isolated from termite gut more suitable microbial feed additives to improve utilization of poor quality fibers~~ [16].

Coptotermes heimi are the most predominant lower termites the in semi-arid regions of India [19]. Till date, little information is available regarding origin and evolution of *Coptotermes heimi* and~~,~~ no research has been done on their gut microbiota's abundance.~~however, no study has been conducted on the abundance of its gut microbiota~~. It is hypothesized that *Coptotermes heimi* ~~harbours~~ harbour a diverse species of fiber degrading bacteria with promising fibrolytic enzyme potential~~and these indigenous isolates would function assecondary fibrolytic enzyme source~~. Therefore, the present study was undertaken to isolate and identify novel fibrolytic bacteria from the gut of *Coptotermes heimi* ~~microbiota never studied before~~ and ~~-~~to evaluate their capacity to break down plant fibre in terms of enzymatic activity~~to assess their fibre degrading potential in terms of enzymatic activity~~ and potential to improve digestibility of roughage~~-~~based diets~~ for improving lignocellulose biodegradability in ruminants~~.

Materials and methods

Specimen collection

The ~~Present~~ present study was conducted ~~in~~ at and surrounding areas ~~the semi-arid region~~ of ICAR-Central Sheep and Wool Research Institute campus, Rajasthan, India~~ and surrounding areas~~. ~~Termite s~~The termite samples of *Coptotermes heimi* were collected from nests on *Ailanthus excelsa*, *Azadirachta indica*, *Albizia lebbeck*, *Cassia fistula*, *Mangifera indica* and *Eucalyptus camaldulensis*~~ trees~~. A total of ninety-seven ~~Ninety seven~~ samples of wood eating termites were collected ~~on~~ (in March 2019) ~~.~~ and ~~After collection, samples were~~ transported to laboratory and washed with water to remove dirt. Thereafter, the termite~~'s~~ samples were washed with 70% ethanol followed by ~~autoclaved~~ distilled water for surface sterilization. The termite's ~~Heads~~ head ~~were~~ was removed using ~~syringe~~ sterilized needle and the entire gut was excised out ~~from all termites~~ [20]. The ~~Gut~~ gut samples were macerated and ~~further~~ processed for isolation of fibre degrading bacteria.

Isolation of fibre degrading bacteria

For isolation of fiber degrading bacteria, M10 medium was used with slight modification which ~~consisted~~ comprised of 15 ml mineral solution-I (0.3% K_2HPO_4), 15 ml mineral solution –II (0.3% KH_2PO_4, 0.6% $(NH_4)_2SO_4$, 0.6% NaCl, 0.06% ~~$MgSo_4$~~$MgSO_4$.7H_2O, 0.0795% $CaCl_2$.2H_2O), 0.05% tryptone, 0.05% yeast extract powder, 0.1% resazurine, 0.2% microcrystalline cellulose, 0.1 ml Pfenning trace elements, 0.31 ml volatile fatty acid mixture, 0.8% sodium bicarbonate ($NaHCO_3$), 20 ml clarified rumen liquor, 0.05% cystine hydrochloride (Cys-HCL). The ~~Collected~~ collected samples were serially diluted in anaerobic ~~diluent~~diluents [21] up to 10^8 cells/~~mL~~ml. From each dilution, ~~about~~ around 0.5 ~~mL~~ ml of inoculum was inoculated in M10 agar medium (1.5% agar) in Hungate tubes (5 mL) using disposable syringe. The agar tubes were rolled on ice bed for solidification and incubated at 39°C for 48 h~~ at 39°C~~. Presumptive colonies of different colour, size and

Formatted: Not Superscript/ Subscript

shape were picked and inoculated in anaerobically prepared M10 broth medium for sub-culturing in an anaerobic chamber (Bioxia, India). The above process ~~were~~ was repeated at least for 4 times till monoculture was obtained~~observation~~. The ~~-~~pure cultures isolated were stored in 80% anaerobic glycerol stock at -80°C for further use.

Morphological and biochemical characterization of isolates

The ~~Isolates~~ isolates were characterized morphologically ~~characterized~~ by Gram staining [~~21~~22] ~~followed by~~and biochemically by tests ~~such as~~like anaerobiosis, catalase, gas production, motility, H_2S production, indole, methyl red and sugar fermentation ~~attributes~~ [~~22~~23]. The results obtained were compared with Bergey's Manual of Determinative Bacteria for identification [~~23~~24].

Fibrolytic enzyme activity

The experiment was conducted to estimate the fibrolytic enzymatic activity~~For determining the activity of fibrolytic enzymes~~, 96 h old bacterial culture, in three replicates, were treated with lysozyme and sonicated for 6 min~~utes~~ with ~~a~~ 30-sec~~ond~~ pulse rate under ice-cool condition and centrifuged at 14,000×g ~~G~~ [~~22~~23]. The supernatant ~~collected~~ obtained was used as a crude enzyme extract for estimation of fibrolytic enzymes like carboxymethyl cellulase (CMCase), endoglucanase, avicelase (Exoglucanase), α-Amylase (1,4-Gluanohydrolase), xylanase (Endo-1,4-ß-xylanase) and β-glucosidase, filter paper degrading assay (FPase) ~~was determined~~ [~~24~~25, ~~25~~26]. The endoglucanase, exoglucanase, FPase, and α-Amylase enzyme ~~activity~~ activities ~~is~~ were expressed as µmol glucose released per min~~ute~~ per ~~mL~~ ml (U/~~mL~~ml) and ~~the~~ xylanase ~~enzyme~~ activity ~~is~~ was expressed as µmole xylose released per min~~ute~~ per mL (U/mL).

~~For endoglucanase, exoglucanase, α-Amylase, and xylanase enzyme activity estimation, a common procedure is used with the different substrates [24].~~ The substrate used for estimation of endoglucanase, exoglucanase, α-Amylase, and xylanase were 1~~-~~% ~~of~~ carboxymethyl cellulose (Sigma-Aldrich, USA), 1% avicel (Sigma-Aldrich, USA), 1% starch (Sigma-Aldrich, USA), and 1% xylan (Sigma-Aldrich, USA), ~~are used as a substrate for endoglucanase, exoglucanase, α-Amylase, and xylanase enzymes~~ respectively while,~~. The~~ the FPase activity was ~~estimated~~ determined using 50 mg Whatman filter paper as the substrate.

To calculate the activity of endoglucanase, exoglucanase, α-Amylase, and FPase enzyme, 0.1% glucose (Sigma-Aldrich, USA) was used as a standard ~~and~~ whereas, for xylanase enzyme~~,~~ 0.1% xylose was used as a standard (Sigma-Aldrich, USA)~~ used as standard~~. ~~For t~~The test ~~, the~~ reaction ~~involved~~ consisted of 0.05 ~~mL~~ ml ~~of~~ enzyme extract, 0.05 ml ~~of the~~ substrate, and 0.1 ~~mL~~ ml ~~of~~ phosphate buffer. The control reaction was identical to the test with the exception of the enzyme extract, which was denatured in the control reaction. The contents in the tubes were mixed ~~well~~ properly ~~in tubes~~ and kept ~~the tubes~~ at 39~~-~~°C for 60 min~~utes~~. Following incubation, 0.5 ml di-nitro salicylic acid reagent (Sigma-Aldrich, USA) was added to the tubes and kept in boiling water bath for 10 min. After 10 min, 0.1 mL of Rochelle salt was added to the tubes and allowed to cool to room temperature and the absorbance was measured at 575 nm against blank. ~~The control sample was prepared by denaturing crude enzyme extract in a boiling water bath and mixed with a substrate, phosphate buffer, and 0.5 mL Di-nitro salicylic acid (DNS) reagent (Sigma-Aldrich, USA). Following incubation, 0.5 mL DNS reagent~~

~~was added to the test. Kept all tubes (control and test) for 10 minutes in a boiling water bath. After boiling, 0.1 mL Rochelle salt was added to the test, and control tubes and cool them at room temperature. The absorbance read against blank at 575 nm.~~

~~For the determination of β-glucosidase enzyme activity, the test, 0.1 mL of enzyme extract incubated for 10 minutes with 0.9 mL of 0.1% PNPG (p-nitrophenol ß-D-glucopyranose) (Hi-media, India) as a substrate. For control, denatured the enzyme by keeping it in a boiling water bath for 3 minutes, mixing 0.1 mL of a denatured enzyme, 0.9 mL of 0.1% PNPG solution, and 1.0 mL of 2% sodium carbonate. After incubation, 1.0 ml 2% sodium carbonate was added to the test to terminate the reaction. The standard curve was prepared with 0.002% p-nitrophenol (Sigma-Aldrich). Absorbance read at 400 nm against reagent blank. Enzyme activity expressed as µmole p-nitrophenol released per minute per mL.~~For the determination of β-glucosidase enzyme activity, 0.9 ml of 0.1% PNPG (p-nitrophenol ß-D-glucopyranose) (Hi-media, India) was incubated for 10 minutes with 0.1 ml of enzyme extract as the substrate. The control tubes contained 0.1 ml denatured enzyme extract as the substrate. Following incubation, 1.0 ml of 2% sodium carbonate was added to the tubes to stop the reaction and absorbance was measured at 400 nm against blank. For the preparation of standard curve, 0.002% p-nitrophenol was used (Sigma-Aldrich) and the enzyme activity was expressed as µmole p-nitrophenol released per min per ml.

Filter Paper ~~Degradation~~ degradation ~~Potential~~potential

For assessing crystalline cellulose degrading potential of the isolates, M10 medium was prepared anaerobically and 25 ×~~x~~ 5 mm strip of filter paper (Whatman No. 1) was added in the media ~~followed by autoclaving~~and autoclaved. The ~~test cultures~~isolates were inoculated in roll tubes containing filter paper media and incubated at 39°C for 10 d. Degradation of crystalline cellulose was determined as amount of filter paper degraded at an interval of 24 h up to 10 d till complete degradation. Degradation potential of test cultures ~~were~~ was measured after comparison with un-inoculated control samples [~~22~~23].

Molecular characterization and sequence homology analysis of ~~16srDNA~~ 16S rRNA gene

Genomic DNA was extracted from the isolates ~~with fibre degradation potential~~ by using DNeasy PowerLyzer PowerSoil Kit~~DNeasy Power lyser Power soil Kit~~ (Qiagen, Germany) as per manufacturer's instructions. The ~~16s~~ 16S ~~rDNA~~ rRNA gene was amplified from the isolated genomic DNA with 27F 5'-AGAGTTTGATCCTGGCTCAG-3' (forward) and 1495R 5' CTACGGCTACCTTGTTACG-3' (reverse) universal 16S ~~rDNA~~ rRNA gene primers. A polymerase chain reaction (PCR) was carried out in an Eppendorf thermal cycler (Germany), each reaction mixture (50 µL) consisted of 10X reaction buffer, 10 µM primers, 10 mM dNTPs, 1.5 U Taq DNA polymerase (Thermo Scientific, Germany), and up to 100 ng DNA template. The annealing temperature was 52◦C and the PCR product size was 1.5 kb. The quality of amplified product was checked by agarose gel electrophoresis. The amplified products were gel purified using GeneJET Gel extraction kit (Cat#K0691, Thermo Scientific, Germany) as per manufacturer's protocol. The purified amplified product was sequenced as per sanger sequencing method by an outsourcing agency (Eurofins, Bengaluru). The nucleotide sequences obtained were trimmed using edit seq software (DNA STAR) and analysed for nearest valid taxon and species using BLAST algorithm [27]. All the aligned sequences were submitted to GenBank.

The evolutionary history was inferred using the Neighbor-Joining method. The tree was drawn to scale with branch lengths (above the branches) in the same units as those of the evolutionary distances used to infer the phylogenetic tree. The evolutionary distances were computed using the Maximum Composite Likelihood method and were in the units of the number of base substitutions per site and the analysis involved 40 nucleotide sequences. All ambiguous positions were removed for each sequence pair (pairwise deletion option). There was a total of 1605 positions in the final dataset and evolutionary analysis was conducted in MEGA11.Polymerase chain reaction (PCR) was performed in an Eppendorf thermal cycler (Germany). Each reaction mixture (50μl) consisted of 10X reaction buffer, 10mM of each primer, 10mM of dNTPs, 1.5U Taq DNA polymerase (Thermo Scientific, Germany) and up to 100ng DNA template. Conditions of PCR cycling were 1 cycle at 94ºC (5 min), 35 cycles at 94ºC (30 sec), 52ºC (30 sec), and 72ºC (45 sec); and a final cycle at 72 ºC for 10 min. The amplified PCR products were checked by gel electrophoresis in 1.2% (w/v) agarose gel containing 0.5 μg/mL ethidium bromide. The 1.5Kb amplified PCR products were gel purified with GeneJET Gel extraction kit (Cat#K0691, Thermo Scientific, Germany) as per the manufacturer's instructions. Purified reactions were sequenced for 16s rDNA region from Eurofins Genomics, India, Pvt. Ltd sequencing services. The sequences obtained were verified in Editseq software and both strands were aligned using BLAST algorithm [26]. Aligned 16s rDNA sequences were further analyzed in BLAST tool on the basis of homologous characteristics with other sequences and phylogenetic analysis was conducted by 1,000 bootstrap and neighbour joining using MEGA 10.0. The aligned sequences were submitted to NCBI GenBank using BankIt submission tool for accession number.

In vitro true digestibility (IVTD)

Screened To evaluate the digestibility potential of the screened isolates an *in vitro* gas production test was conducted isolates were processed in three replicate to determine *in vitro* dry matter (DM) digestibility of feed [2728,2829]. Around 500±10 mg of dried *Cenchrus ciliaris* and *Vigna mungo* straw (80:20) was used as a substrate. Both live culture and autoclaved cultures (control) were used for each isolate. Each treatment had 4 replications. Around 40 ml incubation medium was administered anaerobically in a 100 ml glass syringe along with 3.0 ml of culture. After 48 h incubation at 39ºC, the contents of the syringe were refluxed with 100 ml of neutral detergent solution (sodium lauryl sulphate - 30 g, disodium ethylene diamino tetra acetate - 18.61 g, sodium borate decahydrate - 6.81 g, disodium hydrogen phosphate (anhydrous) - 4.56 g, triethyleneglycol − 10 ml, distilled water - 990 ml per L) in a spoutless beaker, filtered through pre-weighed gooch crucibles and weighed after 24 h oven drying to determine *in vitro* dry matter digestibility [30].One litre Mc Dougall's buffer along with reducing agent was heated on low flame in a 2 L capacity round bottom flask. Contents of the flask were cooled and continuously bubbled with carbon dioxide. Thereafter, 37.5 ml of buffer was added to each serum bottles containing 0.5 g of substrate (*Cenchrus ciliaris* and *Vigna mungo* straw ; 80:20). Bacterial inoculums (2.5 ml culture with OD600 equal to 0.6) along with 10 ml rumen liquor were transferred to each bottles following strict anaerobic conditions. In case of control, 2.5 ml sterile medium was used instead of bacterial culture along with 10 ml rumen. All bottles were properly sealed with rubber stoppers and incubated for 48 h to determine *in vitro* digestibility. The residue was refluxed with 100mL of neutral detergent solution (NDS) for 1h and filtered through sintered glass crucible and weighed after oven drying for 24h to estimate dry matter digestibility [29].

Formatted: Font: Italic

Formatted: Font: Italic

Formatted: Font: Italic

Formatted: Font: Italic

Formatted: Font: Italic

Short chain fatty acid production ~~(SCFAs)~~

After conducting *in vitro* ~~fermentation~~gas production test as mentioned above, the content of the syringe was ~~samples~~ strained with muslin cloth, ~~and~~ collected in scintillation vials and ~~. The samples~~ preserved in ~~-~~-20 °C by adding 0.25% m-phosphoric acid (Hi-media) in the ratio of 9:1 ~~ratio~~. For estimation of short chain fatty acids (SCFAs), ~~estimation~~ the preserved samples were centrifuged at 5000 × ~~grpm~~ for 10 min~~utes~~ and the clear supernatant ~~collected separately and kept on ice~~obtained was injected into the gas chromatogram (GC). The ~~SCFAs estimated in Gas chromatography~~GC (Dani Make, GC-1000, Italy) was equipped with a stainless~~-~~ steel column of 6ft length and 1/8 inch diameter, packed with 10% SP-1200 and~~,~~ 1% phosphoric acid (Supelco). The ~~set~~ temperature ~~for~~ of the injector and detector was 220°C and 250°C, respectively while, the ~~. The~~ oven temperature was ~~set and~~ standardized ~~with~~ at a gradient ~~from~~ of 115°C to 150 °C for separation of each ~~fatty acid~~ molecule. ~~The n~~Nitrogen gas was used as a carrier ~~gas~~ with a pressure of 0.8 ~~-~~bar ~~bar pressure. The other~~while, the pressure of ~~gases~~ air and hydrogen ~~pressure were~~ was 1.0 and 0.8 bar, respectively. The ~~Standard~~ standard used ~~SCFAs~~ was ~~mixture~~ prepared by mixing 65~~mM~~, 20 ~~mM~~, 3 ~~mM~~, 8 ~~mM~~, 2 ~~mM~~ and 2 mM of ~~Acetic~~ acetic acid, propionic acid, iso-butyric acid, butyric acid, iso-valeric acid and valeric acid, respectively.

Formatted: Font: Italic

Statistical analysis

The ~~Data~~ data obtained were analyzed using one-way analysis of variance (ANOVA). ~~and~~ Tukey's b test was used for the post-hoc comparison of different treatment means and statistical differences were considered significant at P<0.05.

Results

Morphological and ~~Biochemical~~ biochemical ~~Characterization~~characterization

Out of 141 isolates, 24 (designated as TM1- TM24) were selected ~~presumptively~~ on the basis of formation of clear zones ~~formed~~ on Congo red plates ~~and designated as TM1-24~~ for further microscopic and biochemical characterization (Table 1). All the isolates were tested for anaerobiosis and catalase reaction ~~to~~ for assessing ~~study~~ their ability to tolerate anoxic environment. Twenty of the 24 isolates showed positive anaerobiosis and negative catalase reaction, indicating that they are obligatory anaerobes while, the other 4 isolates were facultative anaerobes with positive catalase reactions.~~Twenty isolates showed positive anaerobiosis and negative catalase reaction which confirms their obligate anaerobic characteristics, while, remaining isolates were facultative anaerobes and catalase positive~~. Except for TM12, all the isolates produced hydrogen sulfide gas by the converting sulfur to sulfide.~~All isolates produced hydrogen sulphide gas from reduction of sulphur compound to sulphide except TM12~~. None of the isolates produced indole from the reductive deamination of tryptophan, hence all the isolates were indole negative.~~In case of indole test, all isolates tested negative as they failed to produce indole by reductive deamination of tryptophan~~. All the isolates ~~grew with maximum intensity in the presence of~~were able to utilize mannose, sucrose, glucose, carboxy-methyl cellulose, lactose, arabinose, starch, maltose, fructose, xylose and xylan for growth except for ribose sugar (Table 2).

Fibrolytic ~~Enzyme~~ enzyme ~~Activity~~activity

In this study, endoglucanase, exoglucanase, FPase, β-glucosidase, α-amylase, and xylanase enzyme assays were performed to evaluate the fibrolytic potential of the isolates ~~In the present study, enzyme assay performed to assess the fibrolytic potential were endoglucanase, exoglucanase, FPase, β-Glucosidase, α-amylase and xylanase~~ (Table 3). ~~All t~~The isolates showed varying degree of fibrolytic enzyme activity with different substrates like carboxymethyl cellulose, avicel, filter paper, p-nitrophenyl-β-glucoside, starch and xylan. The endoglucanase activity ~~was~~ ranged from 4.83 to 7.24 ~~units per~~U/ ml and highest activity was shown~~ed~~ by isolate TM23 ~~(*Clostridium puniceum strain BL 70/20*)~~. ~~Range of~~The exoglucanase activity among the isolates ranged~~was~~ from 4.24 to 8.40 U/~~mL~~ml. ~~Highest~~highest exoglucanase activity was showed by TM6 ~~(*Pilibacter termitis strain TI-1*)~~ and lowest by TM19 ~~(*Bacillus circulans strain ATCC 4513*)~~. ~~In case of~~The FPase activity was~~,~~ highest ~~activity of 12.05 U/mLwas observed~~ in isolate TM5 (12.05 U/ml) ~~(*Bacillus flexus strain IFO15715*), while~~and, lowest ~~activity of 6.41 U/mL~~ in ~~isolate~~ TM19 (6.41 U/ml) ~~(*Bacillus circulans strain ATCC 4513*)~~. ~~Diverse range of β-glucosidase activity was also observed among the isolates, maximum~~ Maximum β-glucosidase activity was observed in TM18 ~~(*Lactococcus nasutitermitis strain M19*)~~ and lowest in ~~TM1~~ TM1~~(*Bacillus megaterium strain IAM 13418*)~~. ~~Isolates showed~~The α-amylase activity in the isolates ranged~~ing~~ from 2.04 to 12.42 U/mL, where, highest and lowest activity was ~~found~~ observed in isolate TM22 ~~(*Brevibacillus brevis strain DSM 30*)~~ and TM3 ~~(*Citrobacter farmeri strain CDC 2991-8*)~~, respectively. ~~In the present study, b~~Xylanase activity ~~acteria isolated from *Coptotermes heimi*showed xylanase activity~~ ranged~~ing~~ from 0.055 to 12.00 U/ml~~.~~, ~~Maximum~~ maximum being~~activity was observed~~ in TM5 ~~(*Bacillus flexus strain IFO15715*), whereas,~~ and minimum ~~activity~~ in TM20 ~~(*Bacillus circulans strain ATCC 4513*)~~.

Filter paper degradation potential

In the present study, filter paper degradation potential of the isolates was determined at regular intervals ~~of~~ 24 h~~ours~~ up to 10 d ~~after~~ following the inoculation of bacterial isolates in filter paper strip media (Table 4). Among the isolates, TM5 ~~(*Bacillus flexus strain IFO15715*)~~, TM9 ~~(*Bacillus cereus strain ATCC 14579*)~~and TM13 ~~(*Clostridium termitidis*)~~ degraded filter paper in 7 days compared to others and these isolates also ~~had~~ possessed ~~good~~ higher FPase activity ~~than others~~compared to other isolates.

Molecular identification and evolutionary relationships of isolates

~~Amplified~~ The PCR product of ~ 1500 bp size ~~of~1500bp by~~ obtained after 16S ~~rDNA~~ rRNA ~~PCR analysis~~gene amplification (Figure 1) was considered for all the~~20~~ isolates for molecular identification (Table 5). ~~The forward and reverse sequence data were aligned and subjected tonucleotide blast to compare the similarity with database sequences. The i~~The isolates TM1 and TM7 were 99% similar to *Bacillus megaterium* IAM 13418. The ~~Isolates~~ isolates TM2 and TM4 had 98% similarity *Citrobacter freundii* JCM1657. The ~~Isolates~~ isolates TM10 and TM13 were 99% and 98% similar to *Clostridium termitidis*, respectively. The ~~Isolates~~ isolates TM16 and TM 18 were 97% and 99% similar to *Lactococcus nasutitermitis* M19, while, ~~isolates~~ TM17 and TM24 showed 90% and 99% similarity with *Clostridium sporogenes* JCM 1416. The ~~Isolates~~ isolates TM19 and TM20 were 98~~ ~~% similar to *Bacillus circulans* ATCC 4513. ~~Similarity of~~Comparison in similar line, isolate TM3 was 99% similar with *Citrobacter farmeri* CDC 2991, TM5 was 99% similar with *Bacillus flexus* Tl-1, TM8 was 99% similar to *Bacillus licheniformis* ATCC14580, TM9 was 98% similar to *Bacillus cereus* ATCC 14579, TM11 was 100% similar to *Clostridium saccharobutylicum* P262, TM12 was 97% similar to *Clostridium indolis* 7, TM14 was

Formatted: Font: Not Italic, Font color: Auto
Formatted: Font: Not Italic, Font color: Auto
Formatted: Font: Not Italic, Font color: Auto
Formatted: Font: Not Italic, Font color: Auto
Formatted: Font: Not Italic, Font color: Auto
Formatted: Font: Not Italic, Font color: Auto
Formatted: Font: Not Italic, Font color: Auto
Formatted: Font: Not Italic, Font color: Auto
Formatted: Font: Not Italic, Font color: Auto
Formatted: Font: Not Italic, Font color: Auto
Formatted: Font: Not Italic, Font color: Auto

98% similar to *Bacillus oleronius* DSM9356, TM15 was 96% similar to *Clostridium cellulovorans* 743B, TM21 was 98% similar to *Ruminiclostridium papyrosolvens* DSM 2782, TM22 was 96% similar to *Brevibacillus brevis* DSM 30 and TM23 was 96% similar to *Clostridium puniceum* BL 70. All the 16S rRNA gene sequences (TM1 - TM24) were submitted to NCBI GenBank and accession no. A total of 24 sequences were submitted to NCBI gene bank and gene bank accession no. MT356127 to MT356150 was receivedwere obtained and details have been provided in Appendix 1.

Formatted: Font: Not Italic, Font color: Auto

Formatted: Font: Not Italic, Font color: Auto

Formatted: Font: Not Italic, Font color: Auto

Formatted: Font: Not Italic, Font color: Auto

Formatted: Font: Not Italic, Font color: Auto

In vitro dry matter digestibility

Formatted: Font: Italic, Font color: Auto

As depicted in Figure 5, inoculation of bacterial isolated isolates from termite gut enhanced (P<0.05) the dry matter (DM) digestibility of substrate containing *Cenchrus ciliaris* hay and *Vigna mungo* straw by 9 to 36% in comparison with theto control. Inoculation of isolate TM6 (*Pilibacter termitis strain TI-1*) resulted in maximum improvement increase of DM digestibility than control and inoculations withthan other bacterial isolates.

Formatted: Font: Not Italic, Font color: Auto

Short chain fatty acid productions (SCFAs)

Total short chain fatty acids produced production by these isolates ranged from were 64.19 mM to 117.73 mM (Table 6). The concentration of Acetateacetate, propionate, iso-butyrate, butyrate, iso-valerate and valerate were produced by these isolates ranging ranged from 50.37 mM to 83.59 mM, 6.92 mM to 17.87 mM, 1.10 mM to 2.77 mM, 3.08 mM to 10.34 mM, 0.70 mM to 1.56 mM and 0.39 mM to 1.61 mM, respectively. The iIsolate TM6 *Pilibacter termitis* produced highest (P<0.01) acteate (83.59 mM), propionate (17.87 mM), iso-bytyrate (2.77 mM), butyrate (10.34 mM), iso-valerate (1.56 mM) and valerate (1.61 mM) that contribute to significantly higher SCFAs (117.73 mM) (p<0.01) than othercompared to other isolates. The lLowest SCFAs (64.19 mM) was produced by isolate TM7 *Bacillus megaterium*. The isolates TM5 and TM11 produced the Lowest lowest amount of propionate and butyrate produced by isolates TM5 *Bacillus flexus* (6.65mM and 3.08 mM) and TM11 *Clostridium saccharobutylicum* (6.92 mM and 3.67 mM). Among the SCFAs highest (5.93) branch chain fatty acid produced by isolate T6 *Pilibacter termitis* while lowest (2.44) produced by isolate TM18 *Lactococcus nasutitermitis*. The Acetate acetate to propionate ratio (C2:C3) was significantly higher in isolate TM5 *Bacillus flexus* (8.97) and TM11 *Clostridium saccharobutylicum* (8.05) than other isolates.

Discussion

The diverse symbiotic bacterial species found in termite guts contributes to the breakdown of lignocellulose, oligosaccharides, and aromatic compounds, suggesting that these bacteria are important for the digestion of fiber. Termite gut has a diversesymbiotic bacterial population and they participate in degradation of lignocellulose, oligosaccharides and aromatic compounds, which implies these bacteria plays a key role in fibre digestion [3031,3132]. Till date, several natural polymer-degrading microbes have been isolated and characterized from the termite gut for degradation of plant biomass to biofuel. However, the present study aimed identification of promising potentially useful anaerobic fibre degrading bacteria from the gut of wood feeding lower gut termites inhabiting under in the semi-arid environmentsconditions so that these isolates could be manipulated used to improve the usage of fibrous plant biomass in ruminants' feeding.for improving utilisation of fibrous ruminant feeds.

The Isolates isolates showed diverse colony characteristics in terms of size, shape and colour. Fourteen isolates were gram-positive rods, while, remaining 8 were gram negative rods and 2 were gram positive cocci. Similarly, the previous study reported The presence of cellulolytic bacilli and cocci bacteria from in different species of termites is not uncommon having cellulolytic property [3233-3536]. The, isolates were also biochemically characterized and illustrated using Bergey's manual of determinative bacteriology following morphological screeningAfter morphological screening, isolates were subjected to biochemical characterization and illustrated on the basis of Bergey's manual of determinative bacteriology [3637]. In consistent with the present results of the present study, several studies have reported similar biochemical and carbohydrate characterization utilization characteristics from the gut of termites *Reticulitermes santonensis* [3738], *Zootermopsis angusticollis* [3839], *Anacanthotermes vagans* [3940] and *Microcerotermes diversus* [13].

Termites degrade fibre either by their own enzymatic machinery or from enzymes of symbiotic microbes residing in their gut. The association between termites and microbial symbionts in gut makes it an efficient source of enzymes like cellulase, hemicellulase and auxillary enzymes [3031]. Similar to the present findings, a prior study found that bacteria isolated from the gut of higher termite (*Nasutitermes* sp.) showed endoglucanase activity ranging from 0.5 to 6.8 U/mlSimilar to the present findings, previous studyobserved endoglucanase activity ranging from 0.5 to 6.8 U/mL from bacteria isolated from gut of wood feeding higher termite (*Nasutitermes spp.*) [4041]. Furthermore, researcher isolated *Bacillus* sp. was also isolated from wood eating termites *Neotermes* spp and *Cryptotermes brevis* with exhibiting endoglucanase activity of 0.29 and 1.78 U/mLl , respectively from wood feeding termites *Neotermes spp* and *Cryptotermes brevis*, respectively [3435,4142]. Avicel was utilized as a substrate for exoglucanase activity since the cell walls of plants are made of microcrystalline cellulose that is interlaced with biopolymersFor exoglucanase activity, avicel was used as a substrate because plant cell wall consists of biopolymer interwoven microcrystalline cellulose [4243]. In line of the present findings, the an exoglucanase activity of 7.93 to 18.76 U/ml was reported of 7.93 to 18.76 U/mL in bacteria isolated from hindgut of wood eating feeding termite *Amitermes evuncifer Silvestri silvestri* [3334]. A similar FPase activity of 0.196 U/mL and 16.18 U/mL ml was reported in bacterial isolated from the gut of insect and termite *Odontotermes formosanus* respectively [4344,4445]. However, In in the present study, bacteria isolated from the gut of *Coptotermes C. heimi* have higher exoglucanase activity than endoglucanase activity, which depicts that these isolates are better degraders of recalcitrant plantnatural fiber that contains high crystalline cellulose. The β-glucosidase activity enzyme is found in the digestive system of many insects and lower termites, i.e.previous study reported that this enzyme is mostly present in the salivary gland of termites rather than their gut and participates helps in their energy metabolism of termites [4546]. However, the bacteria isolated from the gut of higher termite *Macrotermes annandalei* and revealed presence of β-glucosidase genes that may be involvedβ-glucosidase genes which participate potentially in the host's fiber digestion [4647]. Similarly, *Bacillus* spp. isolated from termite gut had β-glucosidase activity varying between 0.6 and 1.5 U/ml Likewise, *Bacillus* spp.isolated from termite gut with β-glucosidase activity ranging from 0.6 to 1.5 U/mL [4748]. In lower termites, 81% of amylase activity was detected in salivary glands [4849], however, microbial origin amylases were also found in the gut of termites gut produced by the symbiotic bacteria inhabiting in the gut [4950]. Several studies have reported that higher termites have gut microbial population which produces

Formatted: Font: Italic

Formatted: Font: Not Italic

Formatted: Font: Not Italic

Formatted: Font: Italic

Formatted: Font: Not Italic

amylase and it agrees ~~in line~~ with the present findings [~~45~~46, ~~50~~51-~~52~~53]. ~~The role of amylases in termite gut is to extracting organic matter from wood and soil, which is favoured by gut environment.~~ Degradation of hemicelluloses requires enzyme endo-1, 4-xylanase and studies have ~~also~~ reported community of xylanolytic bacteria ~~with xylanolytic potential~~ in the gut of termites ~~gut~~ [~~53~~54]. Similar to ~~the present~~our findings, ~~the~~ bacteria belonging to the *Bacillus* group with promising xylanolytic activity have also been isolated ~~that belonging to *Bacillus* group with promising xylanase activity~~ from wood ~~feeding~~ eating termite ~~(~~*Cryptotermes brevis*~~)~~ ~~and these are potential bio degraders of cellulose and hemicellulose~~ [~~34~~35].

Filter paper contains both amorphous and crystalline cellulose. Degradation of filter paper by any bacteria indicates its cellulolytic potential. Previous studies also revealed ~~the~~ filter paper degradation potential ~~from bacterial~~of bacterial species isolated from termites, however, their filter paper degradation potential was lower as compared to the present findings [~~54~~55,~~55~~56]. The exoglucanase enzyme is mainly responsible for degradation of crystalline cellulose and it is directly related to filter paper degradation potential of the isolates. ~~In the present study~~Thus, an increased filter paper degrading potential of the isolates in comparison to previous reports signify their importance with ~~is justified by~~ high~~er~~ exoglucanase activity ~~of the isolates~~ and could be promising inoculant for commercial enzyme production aimed at plant fiber degradation.

The evolutionary study revealed that 87% of the isolates belonged to the phyla *Firmicutes* and remaining to *Proteobacteria* (Figure 2). ~~Between~~ The majority of isolates from these two phyla belonged to the orders *Bacillales*, *Lactobacillales*, *Enterobacterales*, and *Clostridiales* ~~these two phyla, isolates mostly lied within the order *Clostridiales, Bacillales, Lactobacillales and Enterobacterales*~~. ~~The i~~Isolates under the order *Clostridiales* ~~were identified as member of~~belonged to the ~~family~~ families *Hungaticlostridiaceae*, *Clostridiaceae* and *Lachnospiraceae*. ~~Other~~ Besides the order *Clostridiales*, some isolates ~~under the~~also belonged to ~~–~~order *Bacillales, Lactobacillales* and *Enterobacterales* ~~were identified as members of~~under the family *Bacillaceae, Streptococaceae*, *Enterococcaceae* and *Enterobacteriaceae*, respectively (Figure 3). In the present study, isolates were mostly from *Clostridiaceae* family followed by *Bacillaceae*, *Enterobacteriaceae*, *Streptococcaceae* and *Enterococcaceae*. ~~In consistent with the present findings~~Earlier reports also~~,~~ ~~study reported that,~~observed similar grouping in the phylum *Firmicutes* and order *Clostridiales* ~~majority of bacteria in gut of wood eating lower termites mostly belonged to phylum *Firmicutes* and order *Clostridiales*~~ [~~56~~57]. The ~~16s~~ 16S ~~rDNA~~ rRNA gene-based identification ~~metagenomic study~~ (Figure 4) also revealed that *Fimicutes* phyla and *Clostridia* clustar was the most abundant ~~among all~~ bacterial population ~~and included~~including major representatives of *Peptococcaceae*, *Gracilibacteraceae*, *Clostridiaceae* and *Ruminococcaceae family*. ~~Moreover, p~~In a previous study *Bacteroidetes*, *Fibrobacteres* and *Proteobacteria* were also reported to be present in the hindgut of wood-eating termites [58]~~reported that *Bacteroidetes, Fiberobacters* and *Proteobacteria* were also found in hindgut of wood eating termites~~. The *Clostridales* group contained members of the *Clostridiaceae* family, which is important for the breakdown of plant biomass and indicates an isolate's capacity for cellulolysis ~~The *Clostridales* group included *Clostridiaceae* family which plays key role in plant biomass degradation, signifying cellulolytic ability among isolates~~ [~~57~~58]. ~~The members of *Clostridia* group are involved in fermentation processes within termite gut and also showed potential to convert lignocellulosic biomass to fermented products like alcohol, acetone, acetate, lactate, hydrogen and carbon dioxide [40,57].~~ ~~*Firmicutes* phyla particularly *Lachnospiraceae* members are predominant among the bacterial community in~~

Formatted: Font: Italic, Font color: Auto

Formatted: Font: Italic, Font color: Auto

Formatted: Font: Italic, Font color: Auto

Formatted: Font: Italic, Font color: Auto

Formatted: Font: Italic, Font color: Auto

Formatted: Font: Italic, Font color: Auto

Formatted: Font: Italic, Font color: Auto

Formatted: Font: Italic, Font color: Auto

Formatted: Font: Italic, Font color: Auto

Formatted: Font: Italic, Font color: Auto

mid gut of termite *Nasutitermes corniger*. *Lachnospiraceae* includes diverse species, which can degrade cellulose and hemicellulose Majority of the bacterial populations in the midgut of termite *Nasutitermes corniger* belongs to the phylum *Firmicutes* and are members of the family *Lachnospiraceae* which are capable of breaking down cellulose and hemicelluloses [5859]. *Firmicutes* as most abundant phylum in genomic library of xylan fed termites and suggested that bacteria belonging to *Firmicutes* have the potential to degrade hemicellulose The genetic library of termites fed xylan revealed *Firmicutes* as the most prevalent phylum, and this suggests that *Firmicutes*-related bacteria are able to degrade hemicelluloses [5960]. Moreover, it was reported that a high abundance of *Enterobacteriales* was found in the foregut of termites (*Bullitermes* sp.) after 16S rRNA metagenomic investigation of the community structures of bacteria in the gut Furthermore,it wasreported that the 16S rRNA metagenomic analysis of the community structures of bacteria in gut of termite *Bulbitermes sp*. revealed *Proteobacteria* group in foregut with high abundance of Enterobacteriales [5758].

In this the present study, inoculation of screened isolates enhanced nutrient digestibility of the substrate containing *Cenchrus ciliaris* and *Vigna mungo* straw. It is reported that microbial enzymes which break down structural fibers increases the amount of sugars available to rumen bacteria, which enhances nutrient digestibility Loosening of structural fibresby microbial enzymesmakes more available sugars for rumen microbes and thereby improves digestibility of nutrients [6061]. Several studies have reported a symbiotic relationship between facultative and obligate anaerobes in the termite gut, where, facultative anaerobes scavenge oxygen that penetrates from gut exoskeleton which is essential for fibrolytic activity of obligatory anaerobes [62,63]. In the present study, iThe isolate TM6 had highest endo and exoglucanase activity, which resulted in, enhanced DM digestibility. Likewise, a study reported improvement ind nutrient digestibility of substrate containing wheat straw and date leaves by inoculation of symbiotic bacteria isolated from the termite gut [13]. However, no effect was observed on *in vitro* rumen fermentation and fibre degradation when lignocellulose-degrading bacteria isolated from termite gut was transferred toinnoculated with sheep rumen fluid of sheep [6263]. Several studies have reported that there exists a symbiotic relationship between facultative and obligate anaerobes in the termite gut, where, facultative anaerobes scavenge oxygen that penetrates from gut exoskeleton which is essential for fibrolytic activity of obligatory anaerobes [61,62]. The plant fiber containing ligno-cellulosic and hemicellulosic complexes needs to be broken down for releasing the carbohydrate moiety as energy source and carbon-skeleton for the multiplication of rumen microbial population that aid in nutrient availability to ruminants for increased productivity [15]. Thus, promising bacterial isolates from the termite gut with enhanced fiber-degrading activity could be a potential source for *in vitro* and/or *in vivo* manipulation in maximizing fibrous-feed utilization for animal production.

Conclusions

Till date, this is the first study regarding the existence of isolates with varying fibrolytic enzyme activity in the gut of the wood-eating termite *Coptotermes heimi*anaerobic fibrolytic isolates from gut of wood eating termite *Coptotermes heimi* has not been reported before and, to our knowledge, this the first report on the presence of isolates with diverse fibrolytic enzyme activity in *Coptotermesheimi*gut. Phylogenetic analysis depicted four major families i.e., *Clostridiales*, *Bacillales*, *Lactobacillales* and *Enterobacterales* under phyla *Firmicutes* and

Formatted: Font: Italic, Font color: Auto
Formatted: Font: Italic, Font color: Auto
Formatted: Font: Italic, Font color: Auto
Formatted: Font: Italic, Font color: Auto
Formatted: Font: Italic, Font color: Auto
Formatted: Font: Italic, Font color: Auto
Formatted: Font: Italic, Font color: Auto
Formatted: English (United States)
Formatted: Font: Italic, Font color: Auto
Formatted: Font: Italic, Font color: Auto
Formatted: Font: Italic, Font color: Auto
Formatted: Font: Italic, Font color: Auto
Formatted: English (United States)
Formatted: Font: Italic, Font color: Auto

Proteobacteria with the ability to produce cellulolytic and xylanolytic enzymes~~cellulolytic and xylanolytic enzyme producing potential~~. Furthermore, inoculation of isolated bacterial strains enhanced digestibility of roughage-based diet up to 36% and maximum improvement was observed in case of TM6. ~~However~~Although, further studies are required ~~on~~ to understand the interaction of these isolates with ~~other gut~~the rumen microbiota ~~to understand the ecology of these isolates for obtaining a superior fibrolytic isolate from termite gut as~~ if they are used ~~bio- treatment~~as direct fed microbial for improving degradation of poor quality roughages in ruminants. ~~In addition~~Hence, *in vivo* studies are ~~also~~ required to validate the effect of these exogenous isolates in rumen environment for confirming their efficacy to convert low nutritive biomass as quality ruminant feed. Nonetheless, the identified isolates could serve as promising inoculants in commercial enzyme production aimed at in situ plant-fiber degradation.

Author's contributions: PKK performed isolation and characterisation of isolates, genomic and phylogenetic analysis, SS writing of original draft, data analysis and data interpretation, SK contributed to text preparation and revised the manuscript, AS helped in conceptualization, fund acquisition and supervised the study. All authors read and approved the final version of the manuscript.

Institutional Review Board Statements: Not Applicable

Informed Consent Statement: Not Applicable

Data Availability Statement:

All relevant data are within the manuscript.~~The data are available from the corresponding author upon request.~~

Acknowledgments: Authors are thankful to the Vice Chancellor, Mewar University, Chittorgarh and Director, ICAR-Central Sheep and Wool Research Institute for providing necessary research facilities required during this study.

Conflicts of Interest: The authors declare no competing interest.

References

1. Febrianto F, Pranata AZ, Septiana D, Arinana A, Gumilang A, Hidayat W, Jang J, Lee S, Hwang W and Kim N. Termite resistance of the less known tropical woods species grown in West Java Indonesia. J Korean Wood Sci Technol. 2015; 43:248-257.
2. Thapa S, Mishra J, Arora N, Mishra P, Li H O′ Hair J, Bhatti S and Zhou S. Microbial cellulolytic enzymes: diversity and biotechnology with reference to lignocellulosic biomass degradation. Rev Environ Sci Biotechnol. 2020;19:621-648.
3. Auer L, Lazuka A, Sillam-Dussès D, Miambi E, O'Donohue M and Hernandez-Raquet G. Uncovering the potential of termite gut microbiome for lignocellulose bioconversion in anaerobic batch bioreactors. Front ~~microbial~~Microbiol. 2017;8:2623.

4. Victorica MR, Soria MA, Batista-García RA, Ceja-Navarro JA, Vikram S, Ortiz M Ontañon O, Ghio S, Martínez-Ávila L, García OJQ and Etcheverry C. Neotropical termite microbiomes as sources of novel plant cell wall degrading enzymes. Sci ~~rep~~Rep. 2020;10:1-14.
5. Brune A. Symbiotic digestion of lignocellulose in termite guts. Nat Rev Microbiol. 2014;12:168-180.
6. Inward D, Beccaloni G, Eggleton P. Death of an order: a comprehensive molecular phylogenetic study confirms that termites are eusocial cockroaches. Biol ~~lett~~Lett. Jun. 2007;22:331-5.
7. Slaytor M. Cellulose digestion in termites and cockroaches: what role do symbionts play?. Com Biochem Physiol Part B: Com Biochem. 1992;103:775-784.
8. Tokuda G and Watanabe H. Hidden cellulases in termites: revision of an old hypothesis. Biol lett. 2007;3:336-339.
9. Ni J and Tokuda G. Lignocellulose-degrading enzymes from termites and their symbiotic~~.~~ Microbiota. Biotechnol ~~adv~~Adv. 2013;31:838-850.
10. Husseneder C, Ho HY, Blackwell M. Comparison of the bacterial symbiont composition of the Formosan subterranean termite from its native and introduced range. The ~~open~~ Open ~~microbiol~~ Microbiol ~~jour~~J. 2010; 4:53.
11. Peterson BF, Scharf ME. Lower termite associations with microbes: synergy, protection, and interplay. Front microbiol. 2016;8:7:422.
12. Khan MA, Ahmad W, Paul B. Ecological impacts of termites. Termi Sustain Managem (201-216). Springer, Cham. 2018
13. Azizi-Shotorkhoft A, Mohammadabadi T, Motamedi H, Chaji M and Fazaeli H. Isolation and identification of termite gut symbiotic bacteria with lignocellulose-degrading potential and their effects on the nutritive value for ruminants of some by-products. Anim Feed Sci Technol. 2016;221:234-242.
14. Nasehi M, Torbatinejad NM, Zerehdaran S and Safaei AR. Effect of (Pleurotus florida) fungi on chemical composition and rumen degradability of wheat and barley straw. Iran J Appl Anim Sci. 2014;4(2):257-261.
15. Krause DO, Denman SE, Mackie RI, Morrison M, Rae AL, Attwood GT and McSweeney CS. Opportunities to improve fiber degradation in the rumen: microbiology ecology and genomics. FEMS microbiol rev. 2003;27(5):663-693.
16. Borji M. The survey possibility of straw polysaccharides and lignin degradation by isolated microbiota from termites (Doctoral dissertation PhD Thesis Tarbiat Modares University Tehran (Iran). 2003
17. Kato K, Kozaki S and Sakuranaga M. Degradation of lignin compounds by bacteria from termite guts. Biotechnol let. 1998;20(5):459-462.
18. Watanabe H and Tokuda G. Cellulolytic systems in insects Annu rev entomo.2010;55.
19. Nagaraju DK, Kalleshwaraswamy CM, Iyyanar D, Singh M, Jain RK, Kasturi N, Ranjith M, Mahadevaswamy HM and Asokan R. First interception of two wood feeding potential invasive Coptotermes termite species in India. Int J Tropi Insect Sci. 2020;1-10.
20. El-Sebay Y. A modified El-Sebay trap for subterranean termites fourth Arab. In Congress of Plant Protection Cairo Egypt. 1991;245-247.
21. Sarkar S, Kumawat PK, Bhatt RS, Sahoo A. Isolation and characterization of tanninolytic bacteria from sheep rumen contents: Assessment of tannin degrading, fibrolytic and feed digestibility potential. Small Rumin. Res. 2024; 19:107272.

~~21.~~22. Coico R. Gram staining Curr protoc immunol. 1997;23(1) A-3O

~~22.~~23. Agarwal N, Agarwal I, Kamra DN, Chaudhary LC. Diurnal variations in the activities of hydrolytic enzymes in different fractions of rumen contents of Murrah buffalo. J App Anim Res. 1;18(1):73-80.

~~23.~~24. Dye DW, Lelliott RA, Buchanan R and Gibbons N (1974) Genus II Xanthomonas Dowson 1939 187 Bergey's Manual of Determinative Bacteriology 89.

~~24.~~25. Sahu NP, Kamra DN and Paul SS. Effect of cellulose degrading bacteria isolated from wild and domestic ruminants on in vitro dry matter digestibility of feed and enzyme production. Asian austral J Anim Sci. 2004;17(2):199-202.

~~25.~~26. Gilna VV, Khaleel KM. Cellulase enzyme activity of Aspergillus fumigatus from mangrove soil on lignocellulosic substrate. Rec Resear Sci Technol. 2011;13;3(1).

~~26.~~27. Altschul SF, Madden TL, Schäffer AA, Zhang J, Zhang Z, Miller W, Lipman DJ. Gapped BLAST and PSI-BLAST: a new generation of protein database search programs. Nucleic Acids Res. 1997;25:3389–3402.

~~27.~~28. Tilley JMA and Terry DR. A two-stage technique for the in vitro digestion of forage crops. Grass Forage Sci. 1963;18(2):104-111.

~~28.~~29. Goering HK and Van Soest PJ. Forage fiber analyses: apparatus reagents procedures and some applications (No 379) Agricultural Research Service US Department of Agriculture. 1970.

~~29.~~30. Van Soest PJ, Robertson JB. A laboratory manual for Animal Science 612 Cornell University USA. 1985.

~~30.~~31. Zhou J, Duan J, Gao M, Wang Y, Wang X and Zhao K. Diversity roles and biotechnological applications of symbiotic microorganisms in the gut of termite. Curr microbial. 2019;76(6):755-761.

~~31.~~32. Afzal M, Farman M, Rasib KZ and Qureshi NA. Biocidal action of silver oak (Grevillea robusta) leaf extract on the termite Heterotermes indicola Wasmann (*Blattodea: Rhinotermitidae*). Int Biodeterior Biodegradation. 2019;139:1-10.

~~32.~~33. Butera G, Ferraro C, Alonzo G, Colazza S and Quatrini P. The gut microbiota of the wood-feeding termite Reticulitermes lucifugus. (*Isoptera; Rhinotermitidae*) Annal microbial. 2016;66(1) 253-260.

~~33.~~34. Femi-Ola TO and Oye bamiji BA. Molecular characterization and cellulolytic activities of bacterial isolates from the hindgut of wood-feeding termites *amitermes evuncifersilvestri*. Adv Microbiol. 2019;1-10.

~~34.~~35. Tsegaye B, Balomajumder C and Roy P. Isolation and characterization of novel lignolytic cellulolytic and hemicellulolytic bacteria from wood-feeding termite Cryptotermes brevis Int Microbiol. 2019;22(1):29-39.

~~35.~~36. Nelson K, Muge E and Wamalwa B. Cellulolytic Bacillus species isolated from the gut of the desert locust Schistocerca gregaria Scientific African pe00665. 2021.

~~36.~~37. Holt J, Krieg N, Sneath P, Staley J and Williams S. Bergey's manual of systematic bacteriology 9rd edition The Williams and Wilkins Co Baltimore. 1994.

~~37.~~38. Kuhnigk T and König H. Degradation of dimeric lignin model compounds by aerobic bacteria isolated from the hindgut of xylophagous termites. J Basic Microbiol. 1997;37(3):205-211.

~~38.~~39. Wenzel M, Schönig I, Berchtold M, Kämpfer P and König H. Aerobic and facultatively anaerobic cellulolytic bacteria from the gut of the termite *Zootermopsis angusticollis*. J app microbial. 2002;92(1):32-40.

~~39.~~40. Jalali M. Isolation and Identification of Gut Symbiont Bacteria in the Termite Anacanthotermes Vagans Hagen (Isopetra: Hodotermitidae) and Effects of Two Biopesticide Compound on the Termite MSc Thesis Shahid Chamran University of Ahvaz Iran. 2014.

~~40.~~41. Tokuda G, Mikaelyan A, Fukui C, Matsuura Y, Watanabe H, Fujishima M and Brune A. Fiber-associated spirochetes are major agents of hemicellulose degradation in the hindgut of wood-feeding higher termites PNAS 115(51) E11996-E12004. 2018.

~~41.~~42. Chantarasiri A. Aquatic Bacillus cereus JD0404 isolated from the muddy sediments of mangrove swamps in Thailand and characterization of its cellulolytic activity. Egypt J Aquat Res. 2015;41(3):257-264.

~~42.~~43. Johnsen HR and Krause K. Cellulase activity screening using pure carboxymethylcellulose: application to soluble cellulolytic samples and to plant tissue prints. Int J Mol Sci. 2014;15(1):830-838.

~~43.~~44. Gupta P, Samant K and Sahu A. Isolation of cellulose-degrading bacteria and determination of their cellulolytic potential. Int j microbial. 2012.

~~44.~~45. Liu B, Lin JD, Song SL and Wu DL. The Microbes Cultured and Their Function in the Gut of Termites. Bio Dis Sci. 2012.

~~45.~~46. Purwadari T, Ketaren PP, Sinurat AP, and Sutikno I. Identification and evaluation of fiber hydrolytic enzymes in the extract of termites (Glyptotermes montanus) for poultry feed application. Indo Journ Agri Sci. 2003;4,40-47.

~~46.~~47. Zhang M, Liu N, Qian C, Wang Q, Long Y, Huang Y, Zhou Z and Yan X. Phylogenetic and functional analysis of gut microbiota of a fungus-growing higher termite: Bacteroidetes from higher termites are a rich source of β-glucosidase genes. Micro ecol. 2014;68(2):416-425.

~~47.~~48. Katiyar P, Srivastava SK and Tyagi VK. Measurement of cellulolytic potential of cellulase producing bacteria. Annu Res Rev Biol. 2018;1-9.

~~48.~~49. Veivers PC, O'Brien RW and Slaytor M. Role of bacteria in maintaining the redox potential in the hindgut of termites and preventing entry of foreign bacteria, J Insect Physiol. 1982;28(11):947-951.

~~49.~~50. Mattéotti C, Bauwens J, Brasseur C, Tarayre C, Thonart P, Destain J, Francis F, Haubruge E De Pauw E, Portetelle D and Vandenbol M. Identification and characterization of a new xylanase from Gram-positive bacteria isolated from termite gut (*Reticulitermes santonensis*). Protein Expr and Pur. 2012;83(2):117-127.

~~50.~~51. Mulyani PD, Hamid RM, Janatunaim RZ, Purwestri YA. Amylolytic ability of bacteria isolated from termite (Coptotermes sp.) gut. Indo Journ Biotechnol. 2018;23(1):14-20.

~~51.~~52. Sakolvaree J and Deevong P. Isolation and Characterization of Cellulase Producing Bacteria from the Gut of a Higher Termite *Termes propinquus*. 2016.

~~52.~~53. Tarayre C, Brognaux A, Bauwens J, Brasseur C, Mattéotti C, Millet C, Destain J, Vandenbol M, Portetelle D, De Pauw E and Eric H. Isolation of amylolytic xylanolytic and cellulolytic microorganisms extracted from the gut of the termite Reticulitermes santonensis by means of a micro-aerobic atmosphere. World J Microbiol Biotechnol. 2014;30(5):1655-1660.

~~53.~~54. Breznak JA and Brune A. Role of microorganisms in the digestion of lignocellulose by termites. Annu rev entomol. 1994;39:453-487.

~~54.~~55. Ramin M, Alimon AR, Sijam K and Abdullah N. Filter paper degradation by bacteria isolated from local termite gut Res J Microbiol. 2008;3(8):565-568.

~~55.~~56. Shinde VS, Shinde RM, Agrawal T and Kotasthane AS. Extracellular cellulase activity of termite gut bacteria isolated from different geographical locations of chhattisgarh (CG). India Plant Archiv. 2017;17(1):601-607.

~~56.~~57. Yang H, Schmitt-Wagner D, Stingl U and Brune A. Niche heterogeneity determines bacterial community structure in the termite gut (*Reticulitermes santonensis*). Environ Microbiol. 2005;7(7):916-932.

~~57.~~58. Chew YM, Lye S, Salleh MM and Yahya A. 16S rRNA metagenomic analysis of the symbiotic community structures of bacteria in foregut midgut and hindgut of the wood-feeding termite. Bulbitermes sp Symbiosis. 2018;76(2):187-197.

~~58.~~59. Köhler T, Dietrich C, Scheffrahn RH and Brune A. High-resolution analysis of gut environment and bacterial microbiota reveals functional compartmentation of the gut in wood-feeding higher termites (*Nasutitermes spp*). AEM. 2012;78(13):4691-4701.

~~59.~~60. Miyata R, Noda N, Tamaki H, Kinjyo K, Aoyagi H, Uchiyama H and Tanaka H. Influence of feed components on symbiotic bacterial community structure in the gut of the wood-feeding higher termite *Nasutitermes takasagoensis*. Biosci biotechnol biochem. 2007;71(5):1244-1251.

~~60.~~61. Okano K, Ohkoshi N, Nishiyama A, Usagawa T and Kitagawa M. Improving the nutritive value of madake bamboo Phyllostachys bambusoides for ruminants by culturing with the white-rot fungus *Ceriporiopsis subvermispora*. Anim Feed Sci Technol. 2009;152(3-4):278-285.

~~61.~~62. Azizi A, Sharifi A and Fazaeli H. Effect of one produced slow-release urea component on gas production fermentation nutrient disappearance and activity of microbial enzymes using rumen liquor of sheep. Anim Sci J. 2019;32(122):279-290.

~~62.~~63. Adams L, Boopathy R. Isolation and characterization of enteric bacteria from the hindgut of Formosan termite. Bioresour Technol. 2005;96:1592–8.

Table 1 Morphological and biochemical characteristics of different isolates

Isolates	Gram Stain	Shape	Gas Production Test	Anaerobiosis Test	Catalase Test	Motility Test	H_2S Production Test	Indole Production Test	Methyl Red Test
**TM1**	+	Rod	-	+	-	Motile	+	-	-
**TM2**	-	Rod	+	+	-	Motile	+	-	+
**TM3**	-	Rod	+	+	-	Motile	+	-	+
**TM4**	-	Rod	+	+	-	Motile	+	-	+
**TM5**	-	Rod	-	+	-	Motile	+	-	-
**TM6**	+	Rod	-	+	-	Non motile	+	-	+
**TM7**	+	Rod	-	+	-	Motile	+	-	-
**TM8**	+	Rod	+	-	+	Non motile	+	-	+
**TM9**	+	Rod	-	-	+	Motile	+	-	+
**TM10**	+	Rod	-	+	-	Motile	+	-	+
**TM11**	+	Rod	+	+	-	Non motile	+	-	-
**TM12**	+	Rod	-	+	-	Motile	-	-	+
**TM13**	+	Rod	-	+	-	Motile	+	-	+
**TM14**	-	Rod	+	-	+	Non motile	+	-	+
**TM15**	-	Rod	+	+	-	Non motile	+	-	+
**TM16**	+	Cocci	+	+	-	Non motile	+	-	+
**TM17**	+	Rod	-	+	-	Motile	+	-	+
**TM18**	+	Cocci	+	+	-	Non motile	+	-	+
**TM19**	+	Rod	-	+	-	Non motile	+	-	+
**TM20**	+	Rod	-	+	-	Non motile	+	-	+
**TM21**	-	Rod	+	+	-	Motile	+	-	+
**TM22**	-	Rod	-	-	+	Non motile	+	-	+
**TM23**	+	Rod	+	+	-	Non motile	+	-	+
**TM24**	+	Rod	+	+	-	Motile	+	-	-

(-) Non-utilizers, (+) Utilizers

Table 2 Utilization of various carbohydrates by different isolates

Isolates	1	2	3	4	5	6	7	8	9	10	11	12	13
**TM1**	+	+	+	+	-	-	+	+	+	+	+	+	+
**TM2**	+	+	+	+	+	+	+	+	+	+	+	+	+
**TM3**	+	+	+	+	+	+	+	+	+	+	+	+	-
**TM4**	+	+	+	+	+	+	+	+	+	+	+	+	-
**TM5**	+	+	+	+	+	+	+	+	+	+	+	+	-
**TM6**	+	-	+	+	+	+	+	+	+	+	+	+	-
**TM7**	+	+	+	+	-	-	+	+	+	+	+	+	-
**TM8**	+	+	+	+	+	+	+	+	+	+	+	+	-
**TM9**	+	+	+	+	+	+	+	+	+	+	+	+	-
**TM10**	-	+	+	+	+	-	+	+	+	+	+	+	+
**TM11**	+	+	+	+	+	+	+	+	+	+	+	+	+
**TM12**	+	-	+	+	+	+	+	+	+	+	+	+	+
**TM13**	-	+	+	+	+	+	+	-	+	+	+	+	+
**TM14**	+	+	+	+	+	+	+	-	+	+	+	+	+
**TM15**	-	+	+	+	-	-	+	-	+	+	+	+	+
**TM16**	+	+	+	+	+	+	+	+	+	+	+	+	-
**TM17**	+	+	+	+	+	+	+	+	+	+	+	+	+
**TM18**	-	+	-	+	+	+	+	-	+	+	+	+	-
**TM19**	+	+	+	+	+	+	+	+	+	+	+	+	+
**TM20**	-	+	+	+	+	-	+	-	+	+	+	+	-
**TM21**	-	+	+	+	+	+	+	-	+	+	+	+	+
**TM22**	+	+	+	+	+	+	+	+	+	+	+	+	-
**TM23**	+	+	+	+	+	+	+	+	+	+	+	+	+
**TM24**	+	+	-	+	+	+	+	-	+	+	+	+	+

(-) Non-utilizers, (+) Utilizers

1. Galactose, 2. Mannose, 3. Sucrose, 4. Glucose, 5. Xylose, 6. Xylan, 7. Carboxy-methyl cellulose, 8. Fructose, 9. Lactose, 10. Arabinose, 11. Starch, 12. Maltose, 13. Ribose

Table 3 Fibrolytic enzyme activities of bacterial isolates

**Isolates**	**1**	**2**	**3**	**4**	**5**	**6**
**TM1**	5.21^{abcd}	7.08^{efghi}	8.58^{bcdef}	0.009^{a}	5.17^{cdef}	1.72^{abcd}
**TM2**	5.00^{ab}	6.53^{defghi}	8.03^{abcde}	0.071^{d}	3.35^{abc}	8.44^{kl}
**TM3**	5.69^{abcdef}	5.87^{bcdef}	8.93^{cdef}	0.015^{ab}	2.04^{a}	3.11^{cdefg}
**TM4**	5.51^{abcdef}	6.16 ^{cdef}	8.45^{bcdef}	0.026^{abc}	4.16a^{bcd}	2.88^{bcdef}
**TM5**	4.83^{a}	5.57^{bcde}	12.05^{g}	0.041^{abcd}	5.32c^{defg}	12.00^{m}
**TM6**	7.60^{h}	8.40^{j}	8.67^{bcdef}	0.059^{cd}	7.34f^{ghi}	5.55^{hij}
**TM7**	5.97^{bcdefg}	7.08^{efghi}	8.58^{bcdef}	0.009^{a}	5.17^{cdef}	1.72^{abcd}
**TM8**	6.31^{efg}	7.64^{ghij}	8.35^{bcdef}	0.072^{d}	5.68^{cdefgh}	3.44^{defg}
**TM9**	6.83^{gh}	7.49^{fghij}	9.64^{ef}	0.031^{abcd}	4.36^{abcd}	1.11^{abc}
**TM10**	6.36^{fg}	8.14^{ij}	8.93^{cdef}	0.055^{bcd}	6.03^{defgh}	0.83^{ab}
**TM11**	5.69^{abcdef}	6.22^{cdefg}	8.41^{bcdef}	0.040^{abcd}	2.19^{ab}	2.88^{bcdef}
**TM12**	5.90^{bcdefg}	7.69^{hij}	7.81^{abcd}	0.371^{fg}	10.73^{jk}	5.11^{ghij}
**TM13**	5.30^{abcde}	5.37^{abcde}	9.42^{def}	0.396^{gh}	5.02^{cdef}	2.11^{abcde}
**TM14**	5.92^{cdefg}	6.38^{cdefgh}	8.80^{bcdef}	0.384^{fgh}	9.67^{ij}	10.16^{lm}
**TM15**	5.37^{bcdef}	6.11^{cdef}	7.12^{ab}	0.407^{gh}	8.10^{hi}	0.66^{a}
**TM16**	5.25^{abcd}	5.12^{abc}	8.76^{bcdef}	0.501^{k}	2.34^{ab}	5.83^{ij}
**TM17**	6.21^{defg}	5.53^{bcde}	7.67^{abc}	0.454^{ij}	11.23^{jk}	3.66^{defgh}
**TM18**	5.63^{abcdef}	5.55^{bcde}	7.44^{abc}	0.541^{l}	3.35^{abc}	5.00f^{ghij}
**TM19**	5.65^{abcdef}	4.24^{a}	6.41^{a}	0.465^{jk}	3.76a^{bcd}	3.92^{efghi}
**TM20**	5.17^{abc}	4.79^{ab}	7.52^{abc}	0.423^{hi}	7.85^{ghi}	0.55^{a}
**TM21**	5.32^{abcde}	5.20^{abcd}	7.24^{abc}	0.349^{f}	4.69^{bcde}	7.1^{jk}
**TM22**	6.28^{efg}	6.38^{cdefgh}	9.92^{f}	0.124^{e}	12.42^{k}	8.27^{kl}
**TM23**	7.24^{h}	6.18^{cdef}	7.95^{abcde}	0.051^{abcd}	6.36^{defgh}	6.77^{jk}
**TM24**	5.37^{abcdef}	6.64^{defgh}	8.40^{bcdef}	0.073^{d}	3.08^{abc}	10.22^{lm}
**SEM**	0.08398	0.12864	0.13664	0.0222	0.33731	0.37590
**pValue**	0.000	0.000	0.000	0.000	0.000	0.000

1. Endoglucanase Activity (μmol glucose/h/ml or units/ml), 2. Exoglucanase Activity (μmol glucose/h/ml or units/ml), 3. FPase Activity (μmol glucose/h/ml or units/ml), 4. β-Glucosidase Activity (μmol p-Nitrophenol/min/ml or units/ml), 5. α-Amylase Activity (μmol glucose/h/ml or units/ml), 6. Xylanase Activity (μmol xylose/h/ml or units/ml), $^{a-m}$ means bearing superscript differs significantly in a column ($P < 0.05$).

Table 4 Filter paper degradation potential of different isolates

~~SN~~Isolates	24h	48h	72h	96h	5D	6D	7D	8D
**TM1**	+	++	++	+++	++++	+++++	++++++	CD
**TM2**	+	+	++	+++	++++	+++++	++++++	CD
**TM3**	+	++	++	+++	++++	+++++	++++++	CD
**TM4**	+	++	++	+++	++++	+++++	++++++	CD
**TM5**	++	+++	++++	+++++	+++++	++++++	CD	
**TM6**	+	++	+++	++++	+++++	+++++	++++++	CD
**TM7**	+	++	++	+++	++++	+++++	++++++	CD
**TM8**	+	+	++	+++	++++	+++++	++++++	CD
**TM9**	+	++	+++	++++	+++++	++++++	CD	
**TM10**	+	++	+++	++++	+++++	+++++	++++++	CD
**TM11**	+	++	++	+++	++++	+++++	++++++	CD
**TM12**	+	+	++	+++	++++	+++++	++++++	CD
**TM13**	+	++	+++	++++	+++++	++++++	CD	
**TM14**	+	+	++	+++	++++	+++++	++++++	CD
**TM15**	+	+	++	+++	++++	+++++	++++++	CD
**TM16**	+	+	++	+++	++++	+++++	++++++	CD
**TM17**	+	++	++	+++	++++	+++++	++++++	CD
**TM18**	+	++	++	+++	++++	+++++	++++++	CD
**TM19**	+	+	++	+++	++++	+++++	++++++	CD
**TM20**	+	+	++	+++	++++	+++++	++++++	CD
**TM21**	+	+	+++	+++	++++	+++++	++++++	CD
**TM22**	+	++	++	+++	++++	+++++	++++++	CD
**TM23**	+	+	++	+++	++++	+++++	++++++	CD
**TM24**	+	++	++	+++	++++	+++++	++++++	CD

*CD= Complete Degradation, h=Hours, D= Days

Table 5 16S rDNA sequencing based identification

Isolates	Nearest Valid Taxon	% similarity	Sequence base pairs	Phylum	Family	Gene Bank Accession
TM1	*Bacillus megaterium strain IAM 13418*	99	1473	Firmicutes	Bacillaceae	MT356127
TM2	*Citrobacter freundii strain JCM 1657*	98	1417	Proteobacteria	Enterobacteriaceae	MT356128
TM3	*Citrobacter farmeri strain CDC 2991-81*	99	1472	Proteobacteria	Enterobacteriaceae	MT356129
TM4	*Citrobacter freundii strain JCM 1657*	98	1427	Proteobacteria	Enterobacteriaceae	MT356130
TM5	*Bacillus flexus strain IFO15715*	99	1530	Firmicutes	Bacillaceae	MT356131
TM6	*Pilibacter termitis strain TI-1*	98	1413	Firmicutes	Enterococcaceae	MT356132
TM7	*Bacillus megaterium strain IAM*	99	1482	Firmicutes	Bacillaceae	MT356133
TM8	*Bacillus licheniformis strainATCC14580*	99	1485	Firmicutes	Bacillaceae	MT356134
TM9	*Bacillus cereus strain ATCC 14579*	98	1524	Firmicutes	Bacillaceae	MT356135
TM10	*Clostridium termitidis*	99	1446	Firmicutes	Hungateiclostridiaceae	MT356136
TM11	*Clostridium saccharobutylicum strain P262*	100	1353	Firmicutes	Clostridiaceae	MT356137
TM12	*Clostridium indolis strain 7*	97	1414	Firmicutes	Lachnospiraceae	MT356138
TM13	*Clostridium termitidis*	98	1446	Firmicutes	Hungateiclostridiaceae	MT356139
TM14	*Bacillus oleronius strain DSM 9356*	98	1477	Firmicutes	Bacillaceae	MT356140
TM15	*Clostridium cellulovorans 743B*	96	1447	Firmicutes	Clostridiaceae	MT356141
TM16	*Lactococcus nasutitermitis strain M19*	97	1446	Firmicutes	Streptococaceae	MT356142
TM17	*Clostridium sporogenes strain JCM 1416*	90	1535	Firmicutes	Clostridiaceae	MT356143
TM18	*Lactococcus nasutitermitis strain M19*	99	1440	Firmicutes	Streptococaceae	MT356144
TM19	*Bacillus circulans strain ATCC 4513*	98	1482	Firmicutes	Bacillaceae	MT356145
TM20	*Bacillus circulans strain ATCC 4513*	98	1482	Firmicutes	Bacillaceae	MT356146
TM21	*Ruminiclostridium papyrosolvens DSM 2782*	98	1382	Firmicutes	Hungateiclostridiaceae	MT356147
TM22	*Brevibacillus brevis strain DSM 30*	96	1447	Firmicutes	Paenibacillaceae	MT356148
TM23	*Clostridium puniceum strain BL 70/20*	96	1481	Firmicutes	Clostridiaceae	MT356149
TM24	*Clostridium sporogenes strain JCM 1416*	99	1489	Firmicutes	Clostridiaceae	MT356150

Table 6 Fermentation attributes total short chain fatty acid production

~~S.no.~~Isolates	Acetate(C2) mM	Propionate (C3) mM	Iso-Butyrate (C4i) mM	Butyrate (C4) mM	Iso-Valerate (C5i) mM	Valerate (C5) mM	Total SCFAs mM	BcFA	NGR	C3:(C2+C4)	C2:C3
**TM1**	56.74^{i}	14.26^{f}	1.71^{bcd}	7.34^{g}	0.96^{def}	1.18^{g}	82.19^{hij}	3.85^{cde}	4.16^{ab}	0.22^{defg}	3.98^{abc}
**TM2**	70.72^{k}	14.09^{ef}	2.83^{f}	6.93^{defg}	1.15^{fg}	1.33^{g}	97.05^{k}	5.32^{fg}	4.64^{bc}	0.18^{b}	5.03^{cd}
**TM3**	60.88^{j}	14.10^{ef}	1.96^{cde}	7.30^{fg}	0.92^{def}	0.98^{f}	86.15^{j}	3.87^{cde}	4.42^{abc}	0.21^{cd}	4.32^{abc}
**TM4**	56.96^{i}	13.96^{ef}	1.86^{bcd}	7.16^{fg}	1.01^{efg}	1.27^{g}	82.23^{hij}	4.14^{e}	4.18^{ab}	0.22^{def}	4.09^{abc}
**TM5**	61.35^{j}	6.93^{a}	2.82^{f}	3.08^{ab}	0.96^{def}	0.39^{b}	75.54^{efg}	4.17^{e}	6.55^{e}	0.11^{a}	8.97^{f}
**TM6**	83.59^{l}	17.87^{g}	2.77^{f}	10.34^{h}	1.56^{h}	1.61^{h}	117.73^{l}	5.93^{g}	4.63^{bc}	0.19^{bc}	4.68^{bcd}
**TM7**	44.82^{b}	10.61^{b}	1.37^{bcd}	5.92^{def}	0.73^{bcd}	0.74^{cde}	64.19^{b}	2.84^{bc}	4.43^{abc}	0.21^{cd}	4.23^{abc}
**TM8**	51.47^{efg}	13.43^{def}	1.54^{bcd}	7.15^{fg}	0.93^{def}	0.96^{f}	75.50^{efg}	3.44^{bcde}	4.11^{ab}	0.23^{defh}	3.84^{ab}
**TM9**	49.26^{de}	13.43^{def}	1.42^{bcd}	7.03^{defg}	0.80^{bcde}	0.97^{f}	72.91^{cdef}	3.19^{bcde}	4.01^{ab}	0.24^{efghi}	3.67^{ab}
**TM10**	44.82^{b}	10.61^{b}	1.37^{bcd}	5.92^{def}	0.73^{bcd}	0.74^{cde}	64.19^{b}	2.84^{bc}	4.43^{abc}	0.21^{cd}	4.23^{abc}
**TM11**	55.17^{hi}	6.92^{a}	2.60^{e}	3.67^{bc}	1.23^{g}	0.56^{c}	70.15^{bcde}	4.39^{ef}	5.96^{de}	0.12^{a}	8.05^{e}
**TM12**	63.17^{j}	11.29^{bc}	2.05^{de}	4.46^{c}	1.13^{fg}	0.90^{ef}	83.00^{ij}	4.08^{de}	4.97^{c}	0.17^{b}	5.61^{d}
**TM13**	51.44^{efg}	13.14^{cdef}	1.53^{bcd}	6.74^{defg}	0.82^{bcde}	0.97^{f}	74.65^{defg}	3.32^{bcde}	4.15^{ab}	0.23^{defg}	3.92^{ab}
**TM14**	44.26^{b}	12.10^{bcde}	1.37^{bcd}	6.14^{defg}	0.71^{bcd}	0.79^{def}	65.39^{b}	2.88^{bcd}	3.98^{ab}	0.24^{fghi}	3.67^{ab}
**TM15**	50.37^{ef}	14.85^{f}	1.44^{bcd}	7.41^{g}	0.90^{cdef}	0.98^{f}	75.96^{efgh}	3.32^{bcde}	3.78^{a}	0.26^{i}	3.40^{a}
**TM16**	55.47^{hi}	14.10^{ef}	1.54^{bcd}	7.15^{fg}	0.93^{def}	0.96^{f}	80.16^{ghij}	3.44^{bcde}	4.20^{abc}	0.23^{defg}	3.96^{abc}
**TM17**	45.67^{bc}	13.23^{cdef}	1.30^{abcd}	6.55^{defg}	0.71^{bcd}	0.75^{cde}	68.20^{bcd}	2.76^{bc}	3.86^{ab}	0.25^{hi}	3.46^{a}
**TM18**	48.24^{cde}	11.22^{b}	1.17^{abc}	5.74^{de}	0.65^{bc}	0.62^{cd}	67.63^{bc}	2.44^{b}	4.55^{abc}	0.21^{cd}	4.30^{abc}
**TM19**	49.25^{de}	13.89^{ef}	1.20^{abc}	6.65^{defg}	0.71^{bcd}	0.78^{def}	72.48^{cdef}	2.70^{bc}	3.93^{ab}	0.25^{ghi}	3.55^{a}
**TM20**	49.96^{def}	13.34^{def}	1.10^{ab}	6.91^{defg}	0.73^{bcd}	0.81^{def}	72.85^{cdef}	2.64^{bc}	4.16^{ab}	0.23^{deghi}	3.75^{ab}
**TM21**	50.66^{efg}	14.02^{ef}	1.18^{abc}	6.91^{defg}	0.78^{bcde}	0.76^{cde}	74.30^{defg}	2.71^{bc}	4.02^{ab}	0.24^{fghi}	3.62^{ab}
**TM22**	46.86^{bcd}	11.75^{bcd}	1.20^{abc}	5.68^{d}	0.63^{b}	0.65^{cd}	66.77^{bc}	2.48^{b}	4.27^{abc}	0.22^{defg}	3.99^{abc}
**TM23**	53.80^{ghi}	14.54^{f}	1.21^{abc}	7.09^{efg}	0.75^{bcde}	0.79^{def}	78.19^{fghi}	2.76^{bc}	4.09^{ab}	0.24^{efghi}	3.70^{ab}
**TM24**	53.12^{fgh}	13.22^{cdef}	1.29^{abcd}	6.93^{defg}	0.70^{bcd}	0.81^{def}	76.07^{efgh}	2.79b^{c}	4.36^{abc}	0.22^{def}	4.02^{abc}
**Control**	24.63^{a}	5.66^{a}	0.58^{a}	2.04^{a}	0.15^{a}	0.19^{a}	33.26^{a}	0.92^{a}	4.50^{abc}	0.21^{cde}	4.35^{abc}
**SEM**	1.21	0.318	0.071	0.05	0.03	0.03	1.64	0.12	0.07	0.03	0.155

Branch − chain fatty acids (BcFA) = valerate + iso − valerate + iso − butyrate (C5 + C5i + C4i).NGR Non - glucogenic: Glucogenicratio- = [(Acetate + 2 × Butyrate + BcFA ÷ Propionate + BcFA-] or [C2 + 2C4 + C5 + C5i + C4i- ÷ C3 + C5 + C5i + C4i-].a,b,c Means bearing different superscript differ significantly (p < .05). SCFA, short-chain fatty acids; and SEM, standard error of mean.

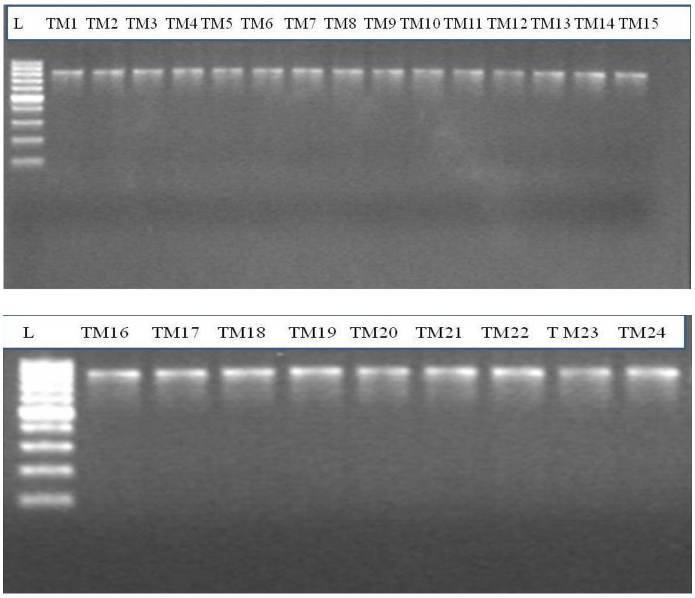

Figure 1.16s rDNA amplification of isolates (TM1-TM24) of size ~1500 bp and L is 1Kb Ladder

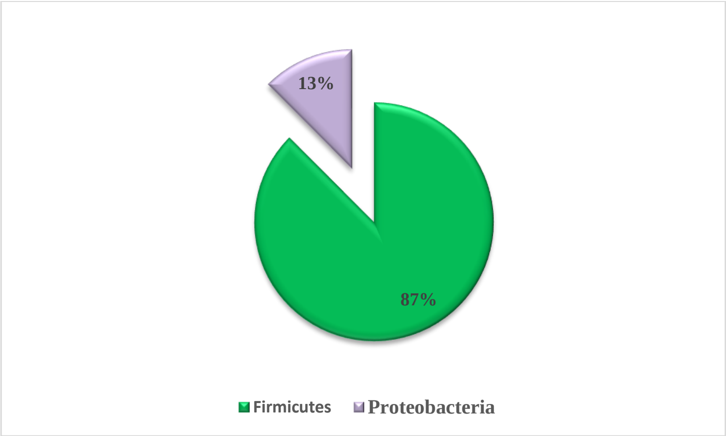

Figure 2. Main phyla abundance of fibrolytic bacteria isolated from gut of wood eating lower termite (*Coptotermes heimi*)

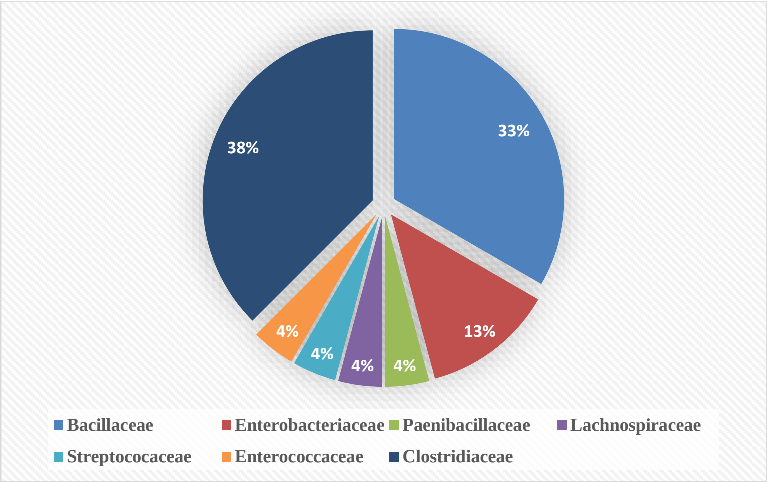

Figure3. Distribution of fibrolytic bacteria identified in the gut of wood eating lower termite (*Coptotermes heimi*) at family level

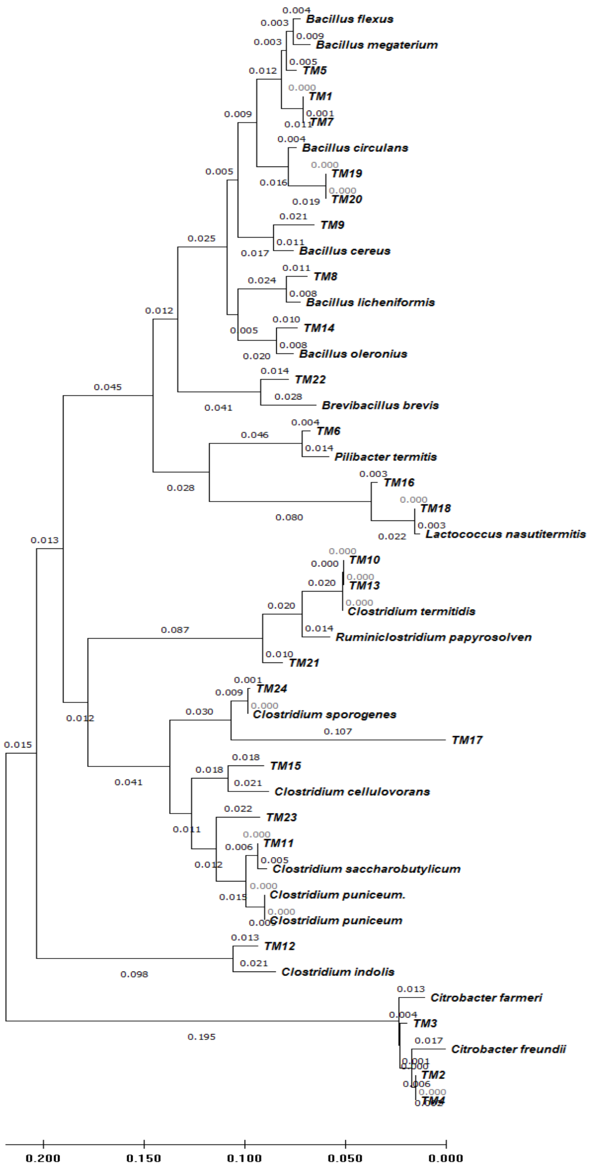

Figure 4 Phylogentic tree based on the 16S rRNA partial sequences of the isolates. Scale bar=1 inferred nucleotide substitution per 50 nucleotides.

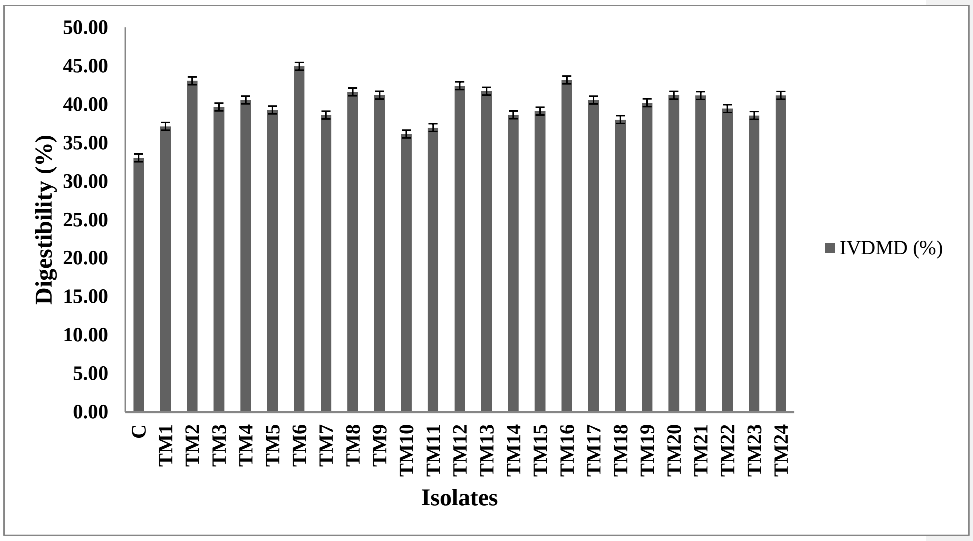

Figure5 Effect of inclusion of bacterial isolates on *in vitro* dry matter (IVDMD) digestibility at 48h post incubation.

RESPONSE TO COMMENTS

Journal Name: PLOS One

Manuscript No: PONE-D-24-05915

Manuscript title: "Lower termite (*Coptotermes heimi*) gut fibrolytic bacterial consortium: Isolation, fiber degradation potential, phylogenetic dynamics and *in vitro* digestibility"

Dear Editor,

Thank you for your useful suggestions on the structure of our manuscript. The suggestions were helpful in improving our manuscript and we have addressed them in the revised version.

Here we are also providing the detailed responses to the comments

**Comments**	**Response to comments**
Line 14: Please provide the full name of the author	Agreed and complied
Line 15: spacing before "Principal".	Corrected
Line 19: change to "The present study was aimed to..."	Sentence modified as suggested
LIne 20: missing a spacing - of anaerobic; from gut; termite Coptotermes; inhabiting under; semi arid.	Needful corrections done
Line 21: missing a spacing before "A total".	Needful corrections done
Line 22: missing a spacing before "isolates".	Needful corrections done
Line 23: activity.	Corrected as suggested
Line 24: The highest........the lowest...	Sentence modified as suggested
Line 25: missing a spacing before "phylogenetic".	Needful corrections done
Line 26: missing a spacing before "In vitro"; between "substrate was".	Needful corrections done
Line 27: change to "TM6 revealed the highest".	Needful corrections done
Line 28: missing a spacing - the gut of C. heimi.	Needful corrections done
Line 30: missing a spacing before "utilization".	Needful corrections done
Line 33: to improve.	Needful corrections done
Line 33: poor quality of fibres.	Needful corrections done
Line 34: missing a spacing before Coptotermes, gut.	Needful corrections done
Line 37: into.	Corrected as suggested
Line 40: higher and lower? incomplete sentence.	Sentence completed
Line 41:harbour.	Corrected as suggested
Line 41: no comma before and after "while".	Needful corrections done
Line 41: contain..	Corrected as suggested
Line 43: work, help.	Corrected as suggested
Line 43: breaking down complex	Corrected as suggested
Line 48: poor-quality. Delete "adding".	Corrected as suggested
Line 50: have the ability.	Corrected as suggested
Line 51: missing a spacing before "Furthermore".	Corrected as suggested
Line 52: missing a comma between acetogenesis and methanogenesis. rewrite the last sentence.	Sentence rewritten as suggested

Line 54: typo error - heimi.	
Line 55: C. heimi.	Needful corrections done
Line 56: C. heimi.	Needful corrections done
Line 56: harbour a diverse.	Corrected as suggested
Line 58: from the gut.	Corrected as suggested
Line 59: of C. heimi.	Corrected as suggested
Line 59: delete microbiota never studied before	Corrected as suggested
Line 60: roughage-based	Corrected as suggested
Line 65: C. heimi.	Corrected as suggested
Line 66: what type of nest? What are the criteria in the site/tree selection?	The Coptotermes is part of family Rhinotermitidae are construct their nests within the wood they consume. These termites carve out intricate networks of tunnels within the wood, devoid of any visible external entrances, except for temporary ones formed during swarming periods. The tunnels within the nests are divided by partitions composed of their waste material and are often covered or lined with a plaster-like substance also made from their waste.<br>The criteria used to identifying termite infested trees are as followed<br>1. Visible Damage<br>2. Frass or Termite Droppings<br>3. Mud Tubes<br>4. Wood Density and Moisture Content
Line 67: delete "trees". Replace "on March" with "in March". How did you collect the samples?	Corrected as suggested
Line 80: 48 h.	Corrected as suggested
Line 82: was repeated.....	Corrected as suggested
Line 93: The experiment was conducted to estimate the enzymatic activity instead of the enzyme itself. Please rephrase the sentence.	Corrected as suggested
Line 96: activities are expressed.	Corrected as suggested
Line 104-105: rewrite.	Sentence rewritten as suggested
Line 107: 39°C.	Corrected as suggested
Line 110: rewrite.	Sentence rewritten as suggested
Line 111-112: rewrite.	Sentence rewritten as suggested
Line 113-116: rewrite.	Sentence rewritten as suggested
Line 123: 10 d.	Corrected as suggested
Line 125: was measured.	Corrected as suggested
Line 128: Get the correct name of the kit.	Needful corrections done
Line 133: missing a spacing between a numeric and a unit.	Corrected as suggested
Line 134: How many different types of conditions?	The conditions are mentioned in text and rewritten for clarity.
Line 135: 72ºC	Needful corrections done
Line 136: 1.5 Kb	Needful corrections done
Line 145: isolates were processed.....replicates...	Text rewritten for clarity.
Line 147: How many types of content?	Text rewritten for clarity.

Line 150: each bottle........condition.	Needful corrections done
Line 152: 100 mL.	Needful corrections done
Line 154: 24 h.	Needful corrections done
Line 156: italic - in vitro. the samples were strained.....	Needful corrections done
Line 157: -20°C	Needful corrections done
Line 160: missing a spacing - 6 ft.	Needful corrections done
Line 160: SP-1200 and 1% phosphoric acid.	Needful corrections done
Line 162: 150°C	Needful corrections done
Line 163: define gases air.	Needful corrections done
Line 164: missing a spacing - CFAs mixture, 65 mM. Small letter for acetic acid.	Corrected as suggested
Line 173: missing a spacing before "All".	Corrected as suggested
Line 174: missing a spacing - their ability.	Corrected as suggested
Line 175: missing a spacing - which confirms.<br>Line 176: missing a spacing before "All".	Corrected as suggested
Line 182: rewrite the sentence.	Sentence rewritten for clarity
Line 185: The endoglucanase activity of TM23 (Clostridium puniceum strain BL 70/20) was ...	Corrected as suggested
Line 186: missing a spacing - and the highest. Delete "was showed by isolate TM23.	Corrected as suggested
Line 186-197: only the scientific names are written in italic. The coding names should be written in a regular format.	Agreed and complied
Line 191: The maximum activity was observed in TM18...	Corrected as suggested
Line 198: missing a spacing - degradation potential.	Corrected as suggested
Line 199: hours or h. Please standardise.	Agreed and complied
Line 201: The coding names should be written in a regular format.	Agreed and complied
Line 204; missing a spacing - 1500 bp. Explain why only 20 isolates were processed molecular identification.	Corrected as suggested. All 24 isolates were processed for molecular identification, 20 was typographical error.
Line 205: missing a spacing - to nucleotide blast.	Needful corrections done
Line 206-217: The coding names should be written in a regular format.	Agreed and complied
Line 217: 24 sequences represent how many bacterial isolates?	Sequences of 24 isolates were submitted to NCBI GenBank
Line 221: no italic for "hay". missing a spacing - straw by.	Agreed and complied
Line 222: The coding names should be written in a regular format.	Agreed and complied
Line 225 - 226: the highest, the lowest.	Needful corrections done
Line 238 - 243: check all the spacing between words. A lot of grammatical errors. Rewrite the last sentence.	Sentence rewritten for clarity
Line 250-251: spacing.	Needful corrections done
Line 252-259: no italic for sp. or spa.	Needful corrections done

Line 259-262: rewrite.	Sentence rewritten for clarity
Line 263: from the gut of C. heimi.	Corrected as suggested
Line 267: from the gut of .	Corrected as suggested
Line 271-272: rewrite.	Sentence rewritten
Line 272: to extract.	Corrected as suggested
Line 295: the most......populations which include..	Corrected as suggested
Line 296: delete "family".	Corrected as suggested
Line 297: in the handgun of.	Corrected as suggested
Line 298: a key role.	Corrected as suggested
Line 299: in the fermentation processes.	Corrected as suggested
Line 302: in the midgut of.	Needful corrections done
Line 304: Firmicutes as the most.	Corrected as suggested
Line 305: it was reported.	Corrected as suggested
Line 307: small letter - revealed.	Corrected as suggested
Line 308: had enhanced the.	Corrected as suggested
Line 309: spacing between texts.	Needful corrections done
Line 310: the highest.	Needful corrections done
Line 319: from the gut.	Corrected as suggested
Line 320: this is the first report.	Needful corrections done
Line 321: the gut of C. heimi	Needful corrections done

Regards

Artabandhu Sahoo
