# Supplementary material for: Lower termite (Coptotermes heimi) gut fibrolytic bacterial consortium: Isolation, phylogenetic characterization, fibre degradation potential and in vitro digestibility
Source: PLoS One. 2025 Mar 10;20(3):e0318090. doi: 10.1371/journal.pone.0318090 (PMC11892857; doi:10.1371/journal.pone.0318090)
Supplement: Attachment — Submitted filename: Comments.pdf [file pone.0318090.s006.pdf]

This manuscript described isolation and characterization of anaerobic fibrolytic bacteria from the gut of *Coptotermes heimi*, screening 24 promising isolates for their potential to improve fiber digestibility in ruminants. Among the 141 isolates obtained, TM23, TM6, and TM22 were found to be the engaging in fibrolytic enzyme activities. The study identified four major bacterial families and demonstrated that the inoculation of these bacteria improved in vitro dry matter digestibility by up to 36.0%. The previous comments have been addressed and manuscript has presented adequate experiments to support the findings and conclusions of conducted study. I would recommend publishing this article in PLOS ONE after a minor revision addressing some additional points.

1) Could author explain in further detail the rationale behind using particularly *Coptotermes heimi* for this study.

2) Line 157-158, authors should provide the concentration of samples used for analysis with GC.

3) In Table 3, based on trends and value of fibrolytic enzyme activites observed, could author comment on finding some interesting correlation between activity and isolates.

4) In Table 4, could author comment on providing some quantitative/numerical data for filter paper degradation potential. Also, please provide remarks on significance of "+" in context of value.
