# Supplementary material for: Lower termite (Coptotermes heimi) gut fibrolytic bacterial consortium: Isolation, phylogenetic characterization, fibre degradation potential and in vitro digestibility
Source: PLoS One. 2025 Mar 10;20(3):e0318090. doi: 10.1371/journal.pone.0318090 (PMC11892857; doi:10.1371/journal.pone.0318090)
Supplement: Attachment — Submitted filename: Response to comments.docx [file pone.0318090.s005.docx]

**RESPONSE TO COMMENTS**

**Journal Name:** PLOS One

**Manuscript No:** PONE-D-24-05915

**Manuscript title**: "Lower termite (*Coptotermes heimi*) gut fibrolytic bacterial consortium: Isolation, fiber degradation potential, phylogenetic dynamics and *in vitro* digestibility"

**Dear Editor**,

Thank you for your useful suggestions on the structure of our manuscript. The suggestions were helpful in improving our manuscript and we have addressed them in the revised version.

Here we are also providing the detailed responses to the comments

| **Comments** | **Response to comments** |
| --- | --- |
| Line 14: Please provide the full name of the author | Agreed and complied |
| Line 15: spacing before "Principal". | Corrected |
| Line 19: change to "The present study was aimed to..." | Sentence modified as suggested |
| LIne 20: missing a spacing - of anaerobic; from gut; termite Coptotermes; inhabiting under; semi arid. | Needful corrections done |
| Line 21: missing a spacing before "A total". | Needful corrections done |
| Line 22: missing a spacing before "isolates". | Needful corrections done |
| Line 23: activity. | Corrected as suggested |
| Line 24: The highest........the lowest... | Sentence modified as suggested |
| Line 25: missing a spacing before "phylogenetic". | Needful corrections done |
| Line 26: missing a spacing before "In vitro"; between "substrate was". | Needful corrections done |
| Line 27: change to "TM6 revealed the highest". | Needful corrections done |
| Line 28: missing a spacing - the gut of C. heimi. | Needful corrections done |
| Line 30: missing a spacing before "utilization". | Needful corrections done |
| Line 33: to improve. | Needful corrections done |
| Line 33: poor quality of fibres. | Needful corrections done |
| Line 34: missing a spacing before Coptotermes, gut. | Needful corrections done |
| Line 37: into. | Corrected as suggested |
| Line 40: higher and lower? incomplete sentence. | Sentence completed |
| Line 41:harbour. | Corrected as suggested |
| Line 41: no comma before and after "while". | Needful corrections done |
| Line 41: contain.. | Corrected as suggested |
| Line 43: work, help. | Corrected as suggested |
| Line 43: breaking down complex | Corrected as suggested |
| Line 48: poor-quality. Delete "adding". | Corrected as suggested |
| Line 50: have the ability. | Corrected as suggested |
| Line 51: missing a spacing before "Furthermore". | Corrected as suggested |
| Line 52: missing a comma between acetogenesis and methanogenesis. rewrite the last sentence.  Line 54: typo error - heimi. | Sentence rewritten as suggested |
| Line 55: C. heimi. | Needful corrections done |
| Line 56: C. heimi. | Needful corrections done |
| Line 56: harbour a diverse. | Corrected as suggested |
| Line 58: from the gut. | Corrected as suggested |
| Line 59: of C. heimi. | Corrected as suggested |
| Line 59: delete microbiota never studied before | Corrected as suggested |
| Line 60: roughage-based | Corrected as suggested |
| Line 65: C. heimi. | Corrected as suggested |
| Line 66: what type of nest? What are the criteria in the site/tree selection? | The Coptotermes is part of family Rhinotermitidae are construct their nests within the wood  they consume. These termites carve out intricate networks of tunnels within the wood, devoid  of any visible external entrances, except for temporary ones formed during swarming periods.  The tunnels within the nests are divided by partitions composed of their waste material and  are often covered or lined with a plaster-like substance also made from their waste.  The criteria used to identifying termite infested trees are as followed  1. Visible Damage  2. Frass or Termite Droppings  3. Mud Tubes  4. Wood Density and Moisture Content |
| Line 67: delete "trees". Replace "on March" with "in March". How did you collect the samples? | Corrected as suggested |
| Line 80: 48 h. | Corrected as suggested |
| Line 82: was repeated..... | Corrected as suggested |
| Line 93: The experiment was conducted to estimate the enzymatic activity instead of the enzyme itself. Please rephrase the sentence. | Corrected as suggested |
| Line 96: activities are expressed. | Corrected as suggested |
| Line 104-105: rewrite. | Sentence rewritten as suggested |
| Line 107: 39°C. | Corrected as suggested |
| Line 110: rewrite. | Sentence rewritten as suggested |
| Line 111-112: rewrite. | Sentence rewritten as suggested |
| Line 113-116: rewrite. | Sentence rewritten as suggested |
| Line 123: 10 d. | Corrected as suggested |
| Line 125: was measured. | Corrected as suggested |
| Line 128: Get the correct name of the kit. | Needful corrections done |
| Line 133: missing a spacing between a numeric and a unit. | Corrected as suggested |
| Line 134: How many different types of conditions? | The conditions are mentioned in text and rewritten for clarity. |
| Line 135: 72ºC | Needful corrections done |
| Line 136: 1.5 Kb | Needful corrections done |
| Line 145: isolates were processed.....replicates... | Text rewritten for clarity. |
| Line 147: How many types of content? | Text rewritten for clarity. |
| Line 150: each bottle........condition. | Needful corrections done |
| Line 152: 100 mL. | Needful corrections done |
| Line 154: 24 h. | Needful corrections done |
| Line 156: italic - in vitro. the samples were strained..... | Needful corrections done |
| Line 157: -20°C | Needful corrections done |
| Line 160: missing a spacing - 6 ft. | Needful corrections done |
| Line 160: SP-1200 and 1% phosphoric acid. | Needful corrections done |
| Line 162: 150°C | Needful corrections done |
| Line 163: define gases air. | Needful corrections done |
| Line 164: missing a spacing - CFAs mixture, 65 mM. Small letter for acetic acid. | Corrected as suggested |
| Line 173: missing a spacing before "All". | Corrected as suggested |
| Line 174: missing a spacing - their ability. | Corrected as suggested |
| Line 175: missing a spacing - which confirms.  Line 176: missing a spacing before "All". | Corrected as suggested |
| Line 182: rewrite the sentence. | Sentence rewritten for clarity |
| Line 185: The endoglucanase activity of TM23 (Clostridium puniceum strain BL 70/20) was ... | Corrected as suggested |
| Line 186: missing a spacing - and the highest. Delete "was showed by isolate TM23. | Corrected as suggested |
| Line 186-197: only the scientific names are written in italic. The coding names should be written in a regular format. | Agreed and complied |
| Line 191: The maximum activity was observed in TM18... | Corrected as suggested |
| Line 198: missing a spacing - degradation potential. | Corrected as suggested |
| Line 199: hours or h. Please standardise. | Agreed and complied |
| Line 201: The coding names should be written in a regular format. | Agreed and complied |
| Line 204; missing a spacing - 1500 bp. Explain why only 20 isolates were processed molecular identification. | Corrected as suggested. All 24 isolates were processed for molecular identification, 20 was typographical error. |
| Line 205: missing a spacing - to nucleotide blast. | Needful corrections done |
| Line 206-217: The coding names should be written in a regular format. | Agreed and complied |
| Line 217: 24 sequences represent how many bacterial isolates? | Sequences of 24 isolates were submitted to NCBI GenBank |
| Line 221: no italic for "hay". missing a spacing - straw by. | Agreed and complied |
| Line 222: The coding names should be written in a regular format. | Agreed and complied |
| Line 225 - 226: the highest, the lowest. | Needful corrections done |
| Line 238 - 243: check all the spacing between words. A lot of grammatical errors. Rewrite the last sentence. | Sentence rewritten for clarity |
| Line 250-251: spacing. | Needful corrections done |
| Line 252-259: no italic for sp. or spa. | Needful corrections done |
| Line 259-262: rewrite. | Sentence rewritten for clarity |
| Line 263: from the gut of C. heimi. | Corrected as suggested |
| Line 267: from the gut of . | Corrected as suggested |
| Line 271-272: rewrite. | Sentence rewritten |
| Line 272: to extract. | Corrected as suggested |
| Line 295: the most......populations which include.. | Corrected as suggested |
| Line 296: delete "family". | Corrected as suggested |
| Line 297: in the handgun of. | Corrected as suggested |
| Line 298: a key role. | Corrected as suggested |
| Line 299: in the fermentation processes. | Corrected as suggested |
| Line 302: in the midgut of. | Needful corrections done |
| Line 304: Firmicutes as the most. | Corrected as suggested |
| Line 305: it was reported. | Corrected as suggested |
| Line 307: small letter - revealed. | Corrected as suggested |
| Line 308: had enhanced the. | Corrected as suggested |
| Line 309: spacing between texts. | Needful corrections done |
| Line 310: the highest. | Needful corrections done |
| Line 319: from the gut. | Corrected as suggested |
| Line 320: this is the first report. | Needful corrections done |
| Line 321: the gut of C. heimi | Needful corrections done |

Regards

Artabandhu Sahoo
