# Supplementary material for: Lower termite (Coptotermes heimi) gut fibrolytic bacterial consortium: Isolation, phylogenetic characterization, fibre degradation potential and in vitro digestibility
Source: PLoS One. 2025 Mar 10;20(3):e0318090. doi: 10.1371/journal.pone.0318090 (PMC11892857; doi:10.1371/journal.pone.0318090)
Supplement: Attachment — Submitted filename: PLOS ONE final Main Manuscript.docx [file pone.0318090.s002.docx]

**Lower termite (*Coptotermes heimi*) gut fibrolytic bacterial consortium: Isolation, fiber degradation potential, phylogenetic dynamics and *invitro* digestibility**

Pankaj Kumar Kumawat^1,3^, Srobana Sarkar^1^,Satish Kumar^3,4^,A. Sahoo^1,2*^

^1^ICAR- Central Sheep and Wool Research Institute, Avikanagar, Rajasthan

^2^ ICAR- National Research Centre on Camel, Bikaner, Rajasthan

^3^Mewar University, Rajasthan

**^*^Corresponding author:** Artabandhu Sahoo, Email address: [sahooarta1@gmail.com](mailto:sarkarsrobana@gmail.com)

Phone No.: 01437- 220162, Fax: 01437- 220163

**Pankaj Kumar Kumawat**: PhD scholar, Mewar University, Rajasthan and Senior Research Fellow, ICAR- Central Sheep and Wool Research Institute, Avikanagar, Rajasthan.

**Srobana Sarkar**: Scientist, ICAR-Central Sheep and Wool Research Institute, Avikanagar, Rajasthan.

**A. Sahoo**: Principal Scientist &Director, ICAR- National Research Centre on Camel, Bikaner, Rajasthan.

**Satish Kumar**:Principal, Mata Basanti Devi School of Biosciences & Biotechnology, Agra.

**^4^Present address**- Mata Basanti Devi School of Biosciences & Biotechnology, Agra

**Abstract**

Lower termites produce wide array of fibrolytic enzymes and serves as prospective microbial enzymes source for enhancing biodegradability of poor quality fibrous feeds. Present study aimed isolation and characterization ofanaerobic fibrolytic bacteria fromgut of termite*Coptotermes heimi* inhabitingunder semiarid conditions for selecting a promising isolate to improve fiber digestibility in ruminants.A total of 141 isolates were obtained from 97 termite samples,isolates were obligatory anaerobes and catalase negative except, TM8, TM9, TM14 and TM22. Overall fibrolytic enzyme activities was highest in isolate TM23, TM6 and TM22 than other isolates. Highest FPase activity was observed in isolate TM5 (12.05 U/ml) while lowest in TM19 (6.41 U/ml).Phylogenetic analysis depicted four major families i.e., *Clostridiales, Bacillales, Lactobacillales* and *Enterobacterales* under phyla *Firmicutes* and *Proteobacteria*.*In vitro* dry matter digestibility of the substratewas increased by 9.4 to 36.0% with administration of isolated bacterial strains. Isolate TM6revealed highest ability to improve nutritive value of feed. The isolates screened from *Coptotermes heimi*gut showed promising fibrolytic enzyme activities and improvement in nutrient digestibility. Administration of these isolates can serve as a biological treatment for improving utilization of low nutritive value feeds in ruminants.Utilization of poor quality feeds plays a pivotal role in sustainable animal agriculture therefore, enhancement of their ruminal degradation is essential. The findings of the present study revealed that fibrolytic bacteria isolated from termite gut can be used as a biological treatment for improve utilization of poor quality fibres.

**Keywords** Lower termites,*Coptotermes heimi,*gut, 16s rDNA, fibrolytic enzymes, digestibility

**Introduction**

Termites acts as “ecosystem engineers” because they help in efficient biodegradation of complex plant biomass in to resourceful bio energy than other invertebrates [1,2]. The potential to degrade fibrous polysaccharide depends on dual enzymatic machinery of termite and its gut microbial symbionts, which makes termite an ideal source of microbial enzymes [3,4]. Termites have the ability to degrade around 65 to 87% hemicellulose and 74 to 99% cellulose from lignocellulosics [5]. Termites are intricately grouped into higher and lower [6]. Higher termites harbours only prokaryotes and lacks protists [7,8,5], while, lower termites contains both protists and bacteria in their gut [9], although scanty information is available about the bacterial population in lower termite gut. These microbes in termite gut works in consortium and helps in breakdown of complex carbohydrate [10,11]. However, the abundance of microbes inhabiting in gut of termites not only depends on their hierarchy but also on geographical location and available vegetation [12].

Utilization of agro-industrial by-products and poor quality fibrous feeds in animal nutrition plays a pivotal role in sustainable agriculture. Effect of several treatments including physical, chemical and biological have been studied for enhancing utility of poor quality feed resources in ruminants [13,14]. Adding exogenous fibrolytic enzymes helps to improve fibre digestibility in ruminants [15]. Earlier studies reported that bacteria isolated from termite gut have potential ability to disintegrate lignin barrier and digest carbohydrate polymers [13,16-18].Furthermore, resemblance between rumen and termite gut environment such as anaerobiosis, reductive acetogenesis methanogenesis and volatile fatty acid production, makes bacteria isolated from termite gut more suitable microbial feed additives to improve utilization of poor quality fibers [16].

*Coptotermes hemi* are the most predominant lower termite in semi-arid regions of India [19]. Till date, little information is available regarding origin and evolution of *Coptotermes hemi*, however, no study has been conducted on the abundance of its gut microbiota. It is hypothesized that *Coptotermes hemi* harbours diverse species of fiber degrading bacteria and these indigenous isolates would function assecondary fibrolytic enzyme source. Therefore, the present study was undertaken to isolate and identify novel fibrolytic bacteria from gut of*Coptotermes hemi* microbiota never studied before and to assess their fibre degrading potential in terms of enzymatic activity and digestibility of roughage based diet for improving lignocellulose biodegradability in ruminants.

**Materials and methods**

**Specimen collection**

Present study was conducted in the semi-arid region of ICAR-Central Sheep and Wool Research Institute campus, Rajasthan, India and surrounding areas. Termite samples of *Coptotermes heimi*  were collected from nests on *[Ailanthus excelsa](https://en.wikipedia.org/wiki/Ailanthus_excelsa)*, [*Azadirachta indica*](https://en.wikipedia.org/wiki/Azadirachta_indica)*,* [*Albizia lebbeck*](https://en.wikipedia.org/wiki/Albizia_lebbeck)*,* [*Cassia fistula*](https://en.wikipedia.org/wiki/Cassia_fistula), [*Mangifera indica*](https://en.wikipedia.org/wiki/Mangifera_indica) and [*Eucalyptus camaldulensis*](https://en.wikipedia.org/wiki/Eucalyptus_camaldulensis) trees. Ninety seven samples of wood eating termites were collected on March 2019. After collection, samples were transported to laboratory and washed with water to remove dirt. Termite’s samples were washed with 70% ethanol followed by autoclaved distilled water for surface sterilization. Heads were removed using syringe needle and entire gut was excised out from all termites [20]. Gut samples were macerated and further processed for isolation of fibre degrading bacteria.

**Isolation of fibre degrading bacteria**

For isolation of fiber degrading bacteria, M10 medium used with slight modification which consisted of 15 ml mineral solution-I (0.3% K_2_HPO_4_^)^, 15 ml mineral solution –II (0.3% KH_2_PO_4_, 0.6% (NH_4_)_2_SO_4,_ 0.6% NaCl, 0.06% MgSo_4_.7H_2_O, 0.0795% CaCl_2_.2H_2_O), 0.05% tryptone, 0.05% yeast extract powder, 0.1% resazurine, 0.2% microcrystalline cellulose, 0.1 ml Pfenning trace elements, 0.31 ml volatile fatty acid mixture, 0.8% sodium bicarbonate (NaHCO_3_), 20 ml clarified rumen liquor, 0.05% cystine hydrochloride (Cys-HCL). Collected samples were serially diluted in anaerobic diluent up to 10^8^ cells/mL. From each dilution, about 0.5 mL of inoculum was inoculated in M10 agar medium (1.5% agar) in Hungate tubes (5 mL) using disposable syringe. The agar tubes were rolled on ice bed for solidification and incubated for 48h at 39°C. Presumptive colonies of different colour, size and shape were picked and inoculated in anaerobically prepared M10 broth medium for sub-culturing in an anaerobic chamber (Bioxia, India). The above process were repeated for 4 times till monoculture observation. The pure cultures were stored in 80% anaerobic glycerol stock at -80°C for further use.

**Morphological and biochemical characterization of isolates**

Isolates were morphologically characterized by Gram staining [21] followed by biochemical test such as anaerobiosis, catalase, gas production, motility, H_2_S production, indole, methyl red and sugar fermentation attributes [22]. The results obtained were compared with Bergey’s Manual of Determinative Bacteria for identification [23].

**Fibrolytic enzyme activity**

For determining the activity of fibrolytic enzymes, 96 h old bacterial culture in three replicates were treated with lysozyme and sonicated for 6 minutes with a 30-second pulse rate under ice-cool condition and centrifuged at 14,000 G[22]. The supernatant collected was used as crude enzyme extract for estimation of fibrolytic enzymes like carboxymethyl cellulase (CMCase), endoglucanase, avicelase (Exoglucanase), α-Amylase (1,4-Gluanohydrolase), Xylanase (Endo-1,4-ß-xylanase) and β-glucosidase, filter paper assay (FPase) was determined [24,25]. The endoglucanase, exoglucanase, FPase, and α-Amylase enzyme activity is expressed as µmol glucose released per minute per mL (U/mL) and the xylanase enzyme activity is expressed as µmole xylose released per minute per mL (U/mL).

For endoglucanase, exoglucanase, α-Amylase, and xylanase enzyme activity estimation, a common procedure is used with the different substrates [24]. The 1 % of carboxymethyl cellulose (Sigma-Aldrich, USA), avicel (Sigma-Aldrich, USA), starch (Sigma-Aldrich, USA), and xylan (Sigma-Aldrich, USA) are used as a substrate for endoglucanase, exoglucanase, α-Amylase, and xylanase enzymes respectively. The FPase activity was estimated using 50 mg Whatman filter paper as the substrate.

To calculate the activity of endoglucanase, exoglucanase, α-Amylase, and FPase enzyme, 0.1% glucose (Sigma-Aldrich, USA) and for xylanase enzyme, 0.1% xylose (Sigma-Aldrich, USA) used as standard. For the test, the reaction involved 0.05 mL of enzyme extract, 0.05 ml of the substrate, and 0.1 mL of phosphate buffer mixed well in tubes and kept the tubes at 39 °C for 60 minutes. The control sample was prepared by denaturing crude enzyme extract in a boiling water bath and mixed with a substrate, phosphate buffer, and 0.5 mL Di-nitro salicylic acid (DNS) reagent (Sigma-Aldrich, USA). Following incubation, 0.5 mL DNS reagent was added to the test. Kept all tubes (control and test) for 10 minutes in a boiling water bath. After boiling, 0.1 mL Rochelle salt was added to the test, and control tubes and cool them at room temperature. The absorbance read against blank at 575 nm.

For the determination of β-glucosidase enzyme activity, the test, 0.1 mL of enzyme extract incubated for 10 minutes with 0.9 mL of 0.1% PNPG (p-nitrophenol ß-D-glucopyranose) (Hi-media, India) as a substrate. For control, denatured the enzyme by keeping it in a boiling water bath for 3 minutes, mixing 0.1 mL of a denatured enzyme, 0.9 mL of 0.1% PNPG solution, and 1.0 mL of 2% sodium carbonate. After incubation, 1.0 ml 2% sodium carbonate was added to the test to terminate the reaction. The standard curve was prepared with 0.002% p-nitrophenol (Sigma-Aldrich). Absorbance read at 400 nm against reagent blank. Enzyme activity expressed as µmole p-nitrophenol released per minute per mL.

**Filter Paper Degradation Potential**

For assessing crystalline cellulose degrading potential, M10 medium was prepared anaerobically and 25x5 mm strip of filter paper (Whatman No. 1) was added in the media followed by autoclaving. The test cultures were inoculated in roll tubes containing filter paper media and incubated at 39°C for 10d. Degradation of crystalline cellulose was determined as amount of filter paper degraded at an interval of 24 h up to 10 d till complete degradation. Degradation potential of test cultures were measured after comparison with un-inoculated control samples [22].

**Molecular characterization and sequence homology analysis of 16srDNA gene**

Genomic DNA was extracted from isolates with fibre degradation potential by using DNeasy Power lyser Power soil Kit (Qiagen, Germany) as per manufacturer’s instructions. The 16s rDNA gene amplified from isolated genomic DNA with 27F 5’-AGAGTTTGATCCTGGCTCAG-3’ (forward) and 1495R 5’ CTACGGCTACCTTGTTACG-3’ (reverse) universal 16S rDNA primers. Polymerase chain reaction (PCR) was performed in an Eppendorf thermal cycler (Germany). Each reaction mixture (50μl) consisted of 10X reaction buffer, 10mM of each primer, 10mM of dNTPs, 1.5U Taq DNA polymerase (Thermo Scientific, Germany) and up to 100ng DNA template. Conditions of PCR cycling were 1 cycle at 94ºC (5 min), 35 cycles at 94ºC (30 sec), 52ºC (30 sec), and 72ºC (45 sec); and a final cycle at 72 ºC for 10 min. The amplified PCR products were checked by gel electrophoresis in 1.2% (w/v) agarose gel containing 0.5 μg/mL ethidium bromide. The 1.5Kb amplified PCR products were gel purified with GeneJET Gel extraction kit (Cat#K0691, Thermo Scientific, Germany) as per the manufacturer's instructions. Purified reactions were sequenced for 16s rDNA region from Eurofins Genomics, India, Pvt. Ltd sequencing services. The sequences obtained were verified in Editseq software and both strands were aligned using BLAST algorithm [26]. Aligned 16s rDNA sequences were further analyzed in BLAST tool on the basis of homologous characteristics with other sequences and phylogenetic analysis was conducted by 1,000 bootstrap and neighbour joining using MEGA 10.0. The aligned sequences were submitted to NCBI GenBank using BankIt submission tool for accession number.

**In vitro true digestibility (IVTD)**

Screened isolatesprocessed in three replicate to determine *in vitro* dry matter (DM) digestibility of feed [27,28]. One litre Mc Dougall’s buffer along with reducing agent was heated on low flame in a 2 L capacity round bottom flask. Contents of the flask were cooled and continuously bubbled with carbon dioxide. Thereafter, 37.5 ml of buffer was added to each serum bottles containing 0.5 g of substrate (*Cenchrus ciliaris* and *Vigna mungo* straw-; 80:20). Bacterial inoculums (2.5 ml culture with OD600 equal to 0.6) along with 10 ml rumen liquor were transferred to each bottles following strict anaerobic conditions. In case of control, 2.5 ml sterile medium was used instead of bacterial culture along with 10 ml rumen. All bottles were properly sealed with rubber stoppers and incubated for 48 h to determine *in vitro* digestibility. The residue was refluxed with 100mL of neutral detergent solution (NDS) for 1h and filtered through sintered glass crucible and weighed after oven drying for 24h to estimate dry matter digestibility [29].

**Short chain fatty acid production (SCFAs)**

After in vitro fermentation, the samples strained with muslin cloth and collected in scintillation vials. The samples preserved in -20 °C by adding 0.25% m-phosphoric acid (Hi-media) in 9:1 ratio. For SCFAs, estimation samples centrifuged at 5000 rpm for 10 minutes and supernatant collected separately and kept on ice. The SCFAs estimated in Gas chromatography (Dani Make, GC-1000, Italy) equipped with stainless steel column of 6ft length and 1/8 inch diameter, packed with 10% SP-1200, 1% phosphoric acid (Supelco). The set temperature for injector and detector was 220°C and 250°C, respectively. The oven temperature was set and standardized with gradient from 115°C to 150 °C for separation of each fatty acid molecule. The nitrogen gas used as carrier gas with 0.8 bar pressure. The other gases air and hydrogen pressure were 1.0 and 0.8 bar, respectively. Standard SCFAsmixture prepared by mixing 65mM, 20 mM, 3 mM, 8 mM, 2 mM and 2 mM of Acetic acid, propionic acid, iso-butyric acid, butyric acid, iso-valeric acid and valeric acid, respectively.

**Statistical analysis**

Data obtained were analyzed using one-way analysis of variance (ANOVA) and Tukey's b test was used for the post-hoc comparison of different treatment means and statistical differences were considered significant at P<0.05.

**Results**

**Morphological and Biochemical Characterization**

Out of 141 isolates, 24 were selected presumptively on the basis of clear zones formed on Congo red plates and designated as TM1-24 for microscopic and biochemical characterization (Table 1).All isolates were tested for anaerobiosis to study theirability to tolerate anoxic environment. Twenty isolates showed positive anaerobiosis and negative catalase reaction whichconfirms their obligate anaerobic characteristics, while, remaining isolates were facultative anaerobes and catalase positive.All isolates produced hydrogen sulphide gas from reduction of sulphur compound to sulphide except TM12. In case of indole test, all isolates tested negative as they failed to produce indole by reductive deamination of tryptophan. All isolates grew with maximum intensity in the presence of mannose, sucrose, glucose, carboxy-methyl cellulose, lactose, arabinose, starch, maltose, fructose, xylose and xylan except ribose sugar (Table 2).

**Fibrolytic Enzyme Activity**

In the present study, enzyme assay performed to assess the fibrolytic potential were endoglucanase, exoglucanase, FPase, β-Glucosidase, α-amylase and xylanase (Table 3). All the isolates showed varying degree of fibrolytic enzyme activity with different substrates like carboxymethyl cellulose, avicel, filter paper, p-nitrophenyl-β-glucoside, starch and xylan.The endoglucanase activity was ranged from 4.83 to 7.24 units per ml andhighest activity was showed by isolate TM23 (*Clostridium puniceum strain BL 70/20*). Range of exoglucanase activityamong isolates was from 4.24 to 8.40 U/mL. Highest exoglucanase activity was showed by TM6 (*Pilibacter termitis strain TI-1*) and lowest by TM19 (*Bacillus circulans strain ATCC 4513*). In case of FPase activity, highest activity of 12.05 U/mLwas observed in isolate TM5 (*Bacillus flexus strain IFO15715*), while, lowest activity of 6.41 U/mL in isolate TM19 (*Bacillus circulans strain ATCC 4513*). Diverse range of β-glucosidase activity was also observed among the isolates, maximum activity was observed TM18 (*Lactococcus nasutitermitis strain M19)* and lowest in TM1 (*Bacillus megaterium strain IAM 13418)*. Isolates showed α-amylase activity ranging from 2.04 to 12.42 U/mL, where, highest and lowest activity was found in isolate TM22 (*Brevibacillus brevis strain DSM 30*) and TM3 (*Citrobacter farmeri strain CDC 2991-8*), respectively. In the present study, bacteria isolated from *Coptotermes heimi*showed xylanase activity ranging from 0.055 to 12.00 U/ml. Maximum activity was observed in TM5 (*Bacillus flexus strain IFO15715*), whereas, minimum activity in TM20 (*Bacillus circulans strain ATCC 4513*).

**Filter paper degradationpotential**

In the present study, filter paper degradation potential was determined at regular interval of 24 hours up to 10 dafter inoculation of bacterial isolates in filter paper strip media (Table 4). Among the isolates, TM5 (*Bacillus flexus strain IFO15715*), TM9 (*Bacillus cereus strain ATCC 14579*)and TM13 (*Clostridium termitidis*) degraded filter paper in 7 days and also had good FPase activity than others.

**Molecular identification and evolutionary relationships of isolates**

Amplified product size of ̴ 1500bp by 16S rDNA PCR analysis (Figure 1) was considered for all20 isolates (Table 5). The forward and reverse sequence data were aligned and subjected tonucleotide blast to compare the similarity with database sequences. The isolates TM1 and TM7 were 99% similar to *Bacillus megaterium IAM 13418*. Isolates TM2 and TM4 had 98% similarity *Citrobacter freundii JCM1657*. Isolates TM10 and TM13 were 99% and 98% similar to *Clostridium termitidis,* respectively. Isolates TM16 and TM 18 were 97% and 99% similar to *Lactococcus nasutitermitis M19*, while, isolates TM17 and TM24 showed 90% and 99% similarity with *Clostridium sporogenes JCM 1416*. Isolates TM19 and TM20 were 98 % to *Bacillus circulans ATCC 4513*. Similarity of TM3 was 99% with*Citrobacter farmeri CDC 2991*, TM5 was 99% similar with *Bacillus flexus Tl-1*, TM8 was 99% similar to *Bacillus licheniformis ATCC14580*, TM9 was 98% similar to *Bacillus cereus ATCC 14579*, TM11 was 100% similar to *Clostridium saccharobutylicum P262*, TM12 was 97% similar to *Clostridium indolis 7*, TM14 was 98% similar to *Bacillus oleronius DSM9356*, TM15 was 96% similar to *Clostridium cellulovorans 743B*, TM21 was 98% similar to *Ruminiclostridium papyrosolvens DSM 2782*, TM22 was 96% similar to *Brevibacillus brevisDSM 30* and TM23 was 96% similar to *Clostridium puniceum BL 70*. A total of 24 sequences were submitted to NCBI gene bank and gene bank accession no. MT356127 to MT356150 was received and details have been provided in Appendix 1.

**In vitrodry matter digestibility**

As depicted in Figure 5, inoculation of bacteria isolated from termite gut enhanced (P<0.05) dry matter (DM) digestibility of substrate containing *Cenchrus ciliaris hay* and *Vigna mungo* strawby 9 to 36% in comparison with the control. Inoculation of isolate TM6 (*Pilibacter termitis strain TI-1*) resulted in maximum improvement of DM digestibility than control and inoculations with other bacteria.

**Short chain fatty acid productions (SCFAs)**

Total short chain fatty acids produced by these isolates were 64.19 mM to 117.73 mM (Table 6). Acetate, propionate, iso-butyrate, butyrate, iso-valerate and valerate were produced by these isolates ranging from 50.37 mM to 83.59 mM, 6.92 mM to 17.87 mM, 1.10 mM to 2.77 mM, 3.08 mM to 10.34 mM, 0.70 mM to 1.56 mM and 0.39 mM to 1.61 mM, respectively. The isolate T6 *Pilibacter termitis* produced highest acteate (83.59 mM), propionate (17.87 mM), iso-bytyrate (2.77 mM), butyrate (10.34 mM), iso-valerate (1.56 mM) and valerate (1.61 mM) that contribute to significantly higher SCFAs (117.73 mM) (p<0.01) than other isolates. The lowest SCFAs (64.19 mM) produced by isolate TM7 *Bacillus megaterium*. Lowest propionate and butyrate produced by isolates TM5 *Bacillus flexus* (6.65mM and 3.08 mM) and TM11 *Clostridium saccharobutylicum* (6.92 mM and 3.67 mM). Among the SCFAs highest (5.93) branch chain fatty acid produced by isolate T6 *Pilibacter termitis* while lowest (2.44) produced by isolate TM18 *Lactococcus nasutitermitis*. Acetate to propionate ratio (C2:C3) was significantly higher in isolate TM5 *Bacillus flexus* (8.97) and TM11 *Clostridium saccharobutylicum* (8.05) than other isolates.

**Discussion**

Termite gut has a diversesymbiotic bacterial population and they participate in degradation of lignocellulose, oligosaccharides and aromatic compounds, which implies these bacteria plays a key role in fibre digestion [30,31]. Till date, several natural polymer-degrading microbes have been isolated and characterized from termite gut for degradation of plant biomass to biofuel. However, the present study aimed identification of promising anaerobic fibre degrading bacteria from wood feeding lower gut termite inhabiting under semi-arid conditions so that these could be manipulated for improving utilisation of fibrous ruminant feeds.

Isolates showed diverse colony characteristics in terms of size, shape and colour. Fourteen isolates were gram-positive rods, while, remaining 8 were gram negative rods and 2 were gram positive cocci. Similarly, the previous study reported bacilli and cocci bacteria from different species of termites having cellulolytic property [32-35]. After morphological screening, isolates were subjected to biochemical characterization and illustrated on the basis of Bergey’s manual of determinative bacteriology [36]. In consistent with the results of the present study, several studies have reported similar biochemical and carbohydrate characterization from gut of termites *Reticulitermes santonensis*[37], *Zootermopsis angusticollis*[38],*Anacanthotermes vagans*[39] and *Microcerotermes diversus*[13].

Termites degrade fibre either by their own enzymatic machinery or from enzymes of symbiotic microbes residing in their gut. The association between termites and microbial symbionts in gut makes it an efficient source of enzymes like cellulase, hemicellulase and auxillary enzymes [30]. Similar to the present findings, previous studyobserved endoglucanase activity ranging from 0.5 to 6.8 U/mL from bacteria isolated from gut of wood feeding higher termite (*Nasutitermes spp.*) [40]. Furthermore, researcher isolated *Bacillus sp*. with endoglucanase activity of 0.29 and 1.78 U/mL respectively from wood feeding termites *Neotermes spp* and *Cryptotermes brevis,* respectively [34,41]. For exoglucanase activity, avicel was used as a substrate because plant cell wall consists of biopolymer interwoven microcrystalline cellulose [42].In line of present findings, the exoglucanase activity reported of 7.93 to 18.76 U/mL in bacteria isolated from hindgut of wood-feeding termite *Amitermes evuncifer Silvestri* [33]. FPase activity of 0.196 U/mL and 16.18 U/mL reported in bacterial isolated from gut of insect and termite *Odontotermes formosanus* respectively [43,44]. In the present study, bacteria isolated from gut of *Coptotermes heimi* have higher exoglucanase activity than endoglucanase activity, which depicts that these isolates are better degraders of natural fiber that contains high crystalline cellulose. β-glucosidase activity is found in digestive system of many insects and lower termites, previous study reported that this enzyme is mostly present in salivary gland of termites rather than gut and participates in energy metabolism of termites [45]. However, the bacteria isolated from gut of higher termite *Macrotermes annandalei* and revealed β-glucosidase genes which participate potentially in host's fiber digestion [46]. Likewise, *Bacillus spp.*isolated from termite gut with β-glucosidase activity ranging from 0.6 to 1.5 U/mL [47]. In lower termites, 81% of amylase activity was detected in salivary glands [48], however, microbial origin amylases were also found in termite gut produced by the symbiotic bacteria inhabiting in gut [49]. Several studies have reported that higher termites have gut microbial population which produces amylase in line with the present findings [45, 50-52]. The role of amylases in termite gut is to extracting organic matter from wood and soil, which is favoured by gut environment. Degradation of hemicelluloses requires enzyme endo-1, 4-xylanase and studies have also reported community of bacteria with xylanolytic potential in termite gut [53]. Similar to the present findings, the bacteria isolated that belonging to *Bacillus* group with promising xylanase activity from wood feeding termite (*Cryptotermes brevis*) and these are potential bio degraders of cellulose and hemicellulose [34].

Filter paper contains both amorphous and crystalline cellulose. Degradation of filter paper by any bacteria indicates its cellulolytic potential. Previous studies also revealed the filter paper degradation potential from bacterial species isolated from termites, however, their filter paper degradation potential was lower as compared to the present findings [54,55]. The exoglucanase enzyme is mainly responsible for degradation of crystalline cellulose and it is directly related to filter paper degradation potential of the isolates. In the present study, increased filter paper degrading potential in comparison to previous reports is justified by higher exoglucanase activity of the isolates.

The evolutionary study revealed that 87% of the isolates belonged to phyla *Firmicutes* and remaining to *Proteobacteria* (Figure 2). Between these two phyla, isolates mostly lied within the order *Clostridiales, Bacillales, Lactobacillales and Enterobacterales*. The isolates under order *Clostridiales* were identified as member of family *Hungaticlostridiaceae, Clostridiaceae* and *Lachnospiraceae*. Other isolates under the order *Bacillales, Lactobacillales* and *Enterobacterales* were identified as members of family *Bacillaceae, Streptococaceae, Enterococcaceae* and *Enterobacteriaceae*, respectively (Figure 3). In the present study, isolates were mostly from *Clostridiaceae* family followed by *Bacillaceae, Enterobacteriaceae, Streptococcaceae* and *Enterococcaceae*. In consistent with the present findings, study reported that, majority of bacteria in gut of wood eating lower termites mostly belonged to phylum *Firmicutes* and order *Clostridiales* [56]. The 16s rDNA metagenomic study (Figure 4) also revealed that Fimicutes phyla and *Clostridia* clustar was most abundant among all bacterial population and included major representatives of *Peptococcaceae, Gracilibacteraceae, Clostridiaceae* and *Ruminococcaceae family*. Moreover, previous study reported that *Bacteroidetes, Fiberobacters* and *Proteobacteria* were also found in hindgut of wood eating termites. The *Clostridales* group included *Clostridiaceae* family which plays key role in plant biomass degradation, signifying cellulolytic ability among isolates [57]. The members of *Clostridia* group are involved in fermentation processes within termite gut and also showed potential to convert lignocellulosic biomass to fermented products like alcohol, acetone, acetate, lactate, hydrogen and carbon dioxide [40,57].*Firmicutes* phyla particularly *Lachnospiraceae* members are predominant among the bacterial community in mid gut of termite *Nasutitermes corniger*. *Lachnospiraceae* includes diverse species, which can degrade cellulose and hemicellulose [58].*Firmicutes* as most abundant phylum in genomic library of xylan fed termites and suggested that bacteria belonging to *Firmicutes* have the potential to degrade hemicellulose [59]. Furthermore,it wasreported that the 16S rRNA metagenomic analysis of the community structures of bacteria in gut of termite *Bulbitermes sp*. Revealed *Proteobacteria* group in foregut with high abundance of Enterobacteriales [57].

In this study, inoculation of screened isolates enhanced nutrient digestibility of the substrate. Loosening of structural fibresby microbial enzymesmakes more available sugars for rumen microbes and thereby improves digestibility of nutrients [60]. In the present study, isolate TM6 had highest endo and exoglucanase activity, which resulted in, enhanced DM digestibility. Likewise, a study reported improved nutrient digestibility of wheat straw and date leaves by inoculation of symbiotic bacteria isolated from termite gut [13]. However, no effect was observed on *in vitro* rumen fermentation and fibre degradation when lignocellulose-degrading bacteria from termite was transferred to rumen fluid of sheep [62]. Several studies have reported that there exists a symbiotic relationship between facultative and obligate anaerobes in the termite gut, where, facultative anaerobes scavenge oxygen that penetrates from gut exoskeleton which is essential for fibrolytic activity of obligatory anaerobes [61,62].

**Conclusions**

Till date, anaerobic fibrolytic isolates from gut of wood eating termite *Coptotermes heimi* has not been reported before and, to our knowledge, this the first report on the presence of isolates with diverse fibrolytic enzyme activity in *Coptotermesheimi*gut. Phylogenetic analysis depicted four major families i.e., *Clostridiales, Bacillales, Lactobacillales* and *Enterobacterales* under phyla *Firmicutes* and *Proteobacteria* with cellulolytic and xylanolytic enzyme producing potential. Furthermore, inoculation of isolated bacteria enhanced digestibility of roughage-based diet up to 36%. However, studies are required on interaction of these isolates with other gut microbiota to understand the ecology of these isolates for obtaining a superior fibrolytic isolate from termite gut as bio- treatment for improving degradation of poor quality roughages. In addition, *in vivo* studies are also required to validate the effect of these exogenous isolates in rumen environment for confirming their efficacy to convert low nutritive biomass as ruminant feed.

**Author’s contributions:** PKK performed isolation and characterisation of isolates, genomic and phylogenetic analysis, PKK and SS wrote the manuscript, analysed the data and prepared the figures, SK contributed to text preparation and revised the manuscript, AS helped in conceptualization, arranged funds and supervised the study. All authors read and approved the final version of the manuscript.

**Institutional Review Board Statements:** Not Applicable

**Informed Consent Statement:** Not Applicable

**Data Availability Statement:** The data are available from the corresponding author upon request.

**Acknowledgments:** Authors are thankful to the Vice Chancellor, Mewar University, Chittorgarh and Director, ICAR-Central Sheep and Wool Research Institute for providing necessary research facilities required during this study.

**Conflicts of Interest:**The authors declare no competing interest.

**References**

1. Febrianto F, Pranata AZ, Septiana D, Arinana A, Gumilang A, Hidayat W, Jang J, Lee S, Hwang W and Kim N. Termite resistance of the less known tropical woods species grown in West Java Indonesia. J Korean Wood Sci Technol. 2015; 43:248-257.
2. Thapa S, Mishra J, Arora N, Mishra P, Li H O′ Hair J, Bhatti S and Zhou S. Microbial cellulolytic enzymes: diversity and biotechnology with reference to lignocellulosic biomass degradation. Rev Environ Sci Biotechnol. 2020;19:621-648.
3. Auer L, Lazuka A, Sillam-Dussès D, Miambi E, O'Donohue M and Hernandez-Raquet G. Uncovering the potential of termite gut microbiome for lignocellulose bioconversion in anaerobic batch bioreactors. Front microbial. 2017;8:2623.
4. Victorica MR, Soria MA, Batista-García RA, Ceja-Navarro JA, Vikram S, Ortiz M Ontañon O, Ghio S, Martínez-Ávila L, García OJQ and Etcheverry C. Neotropical termite microbiomes as sources of novel plant cell wall degrading enzymes. Sci rep. 2020;10:1-14.
5. Brune A. Symbiotic digestion of lignocellulose in termite guts. Nat Rev Microbiol. 2014;12:168-180.
6. Inward D, Beccaloni G, Eggleton P. Death of an order: a comprehensive molecular phylogenetic study confirms that termites are eusocial cockroaches. Biol lett. Jun. 2007;22:331-5.
7. Slaytor M. Cellulose digestion in termites and cockroaches: what role do symbionts play?. Com Biochem Physiol Part B: Com Biochem. 1992;103:775-784.
8. Tokuda G and Watanabe H. Hidden cellulases in termites: revision of an old hypothesis. Biol lett. 2007;3:336-339.
9. Ni J and Tokuda G. Lignocellulose-degrading enzymes from termites and their symbiotic. microbiota Biotechnol adv. 2013;31:838-850.
10. Husseneder C, Ho HY, Blackwell M. Comparison of the bacterial symbiont composition of the Formosan subterranean termite from its native and introduced range. The open microbiol jour. 2010; 4:53.
11. Peterson BF, Scharf ME. Lower termite associations with microbes: synergy, protection, and interplay. Front microbiol. 2016;8:7:422.
12. Khan MA, Ahmad W, Paul B. Ecological impacts of termites. Termi Sustain Managem (201-216). Springer, Cham. 2018
13. Azizi-Shotorkhoft A, Mohammadabadi T, Motamedi H, Chaji M and Fazaeli H. Isolation and identification of termite gut symbiotic bacteria with lignocellulose-degrading potential and their effects on the nutritive value for ruminants of some by-products. Anim Feed Sci Technol. 2016;221:234-242.
14. Nasehi M, Torbatinejad NM, Zerehdaran S and Safaei AR. Effect of (Pleurotus florida) fungi on chemical composition and rumen degradability of wheat and barley straw. Iran J Appl Anim Sci. 2014;4(2):257-261.
15. Krause DO, Denman SE, Mackie RI, Morrison M, Rae AL, Attwood GT and McSweeney CS. Opportunities to improve fiber degradation in the rumen: microbiology ecology and genomics. FEMS microbiol rev. 2003;27(5):663-693.
16. Borji M. The survey possibility of straw polysaccharides and lignin degradation by isolated microbiota from termites (Doctoral dissertation PhD Thesis Tarbiat Modares University Tehran (Iran). 2003
17. Kato K, Kozaki S and Sakuranaga M. Degradation of lignin compounds by bacteria from termite guts. Biotechnol let. 1998;20(5):459-462.
18. Watanabe H and Tokuda G. Cellulolytic systems in insects Annu rev entomo.2010;55.
19. Nagaraju DK, Kalleshwaraswamy CM, Iyyanar D, Singh M, Jain RK, Kasturi N, Ranjith M, Mahadevaswamy HM and Asokan R. First interception of two wood feeding potential invasive Coptotermes termite species in India. Int J Tropi Insect Sci. 2020;1-10.
20. El-Sebay Y. A modified El-Sebay trap for subterranean termites fourth Arab. In Congress of Plant Protection Cairo Egypt. 1991;245-247.
21. Coico R. Gram staining Curr protoc immunol. 1997;23(1) A-3O
22. Agarwal N, Agarwal I, Kamra DN, Chaudhary LC. Diurnal variations in the activities of hydrolytic enzymes in different fractions of rumen contents of Murrah buffalo. J App Anim Res. 1;18(1):73-80.
23. Dye DW, Lelliott RA, Buchanan R and Gibbons N (1974) Genus II Xanthomonas Dowson 1939 187 Bergey's Manual of Determinative Bacteriology 89.
24. Sahu NP, Kamra DN and Paul SS. Effect of cellulose degrading bacteria isolated from wild and domestic ruminants on in vitro dry matter digestibility of feed and enzyme production. Asian austral J Anim Sci. 2004;17(2):199-202.
25. Gilna VV, Khaleel KM. Cellulase enzyme activity of Aspergillus fumigatus from mangrove soil on lignocellulosic substrate. Rec Resear Sci Technol. 2011;13;3(1).
26. Altschul SF, Madden TL, Schäffer AA, Zhang J, Zhang Z, Miller W, Lipman DJ. Gapped BLAST and PSI-BLAST: a new generation of protein database search programs. Nucleic Acids Res. 1997;25:3389–3402.
27. Tilley JMA and Terry DR. A two‐stage technique for the in vitro digestion of forage crops. Grass Forage Sci. 1963;18(2):104-111.
28. Goering HK and Van Soest PJ. Forage fiber analyses: apparatus reagents procedures and some applications (No 379) Agricultural Research Service US Department of Agriculture. 1970.
29. Van Soest PJ, Robertson JB. A laboratory manual for Animal Science 612 Cornell University USA. 1985.
30. Zhou J, Duan J, Gao M, Wang Y, Wang X and Zhao K. Diversity roles and biotechnological applications of symbiotic microorganisms in the gut of termite. Curr microbial. 2019;76(6):755-761.
31. Afzal M, Farman M, Rasib KZ and Qureshi NA. Biocidal action of silver oak (Grevillea robusta) leaf extract on the termite Heterotermes indicola Wasmann (*Blattodea: Rhinotermitidae*). Int Biodeterior Biodegradation. 2019;139:1-10.
32. Butera G, Ferraro C, Alonzo G, Colazza S and Quatrini P. The gut microbiota of the wood-feeding termite Reticulitermes lucifugus. (*Isoptera; Rhinotermitidae*) Annal microbial. 2016;66(1) 253-260.
33. Femi-Ola TO and Oye bamiji BA. Molecular characterization and cellulolytic activities of bacterial isolates from the hindgut of wood-feeding termites *amitermes evuncifersilvestri*.  Adv Microbiol. 2019;1-10.
34. Tsegaye B, Balomajumder C and Roy P. Isolation and characterization of novel lignolytic cellulolytic and hemicellulolytic bacteria from wood-feeding termite Cryptotermes brevis Int Microbiol. 2019;22(1):29-39.
35. Nelson K, Muge E and Wamalwa B. Cellulolytic Bacillus species isolated from the gut of the desert locust Schistocerca gregaria Scientific African pe00665. 2021.
36. Holt J, Krieg N, Sneath P, Staley J and Williams S. Bergey's manual of systematic bacteriology 9rd edition The Williams and Wilkins Co Baltimore. 1994.
37. Kuhnigk T and König H. Degradation of dimeric lignin model compounds by aerobic bacteria isolated from the hindgut of xylophagous termites. J Basic Microbiol. 1997;37(3):205-211.
38. Wenzel M, Schönig I, Berchtold M, Kämpfer P and König H. Aerobic and facultatively anaerobic cellulolytic bacteria from the gut of the termite *Zootermopsis angusticollis*. J app microbial. 2002;92(1):32-40.
39. Jalali M. Isolation and Identification of Gut Symbiont Bacteria in the Termite Anacanthotermes Vagans Hagen (Isopetra: Hodotermitidae) and Effects of Two Biopesticide Compound on the Termite MSc Thesis Shahid Chamran University of Ahvaz Iran. 2014.
40. Tokuda G, Mikaelyan A, Fukui C, Matsuura Y, Watanabe H, Fujishima M and Brune A. Fiber-associated spirochetes are major agents of hemicellulose degradation in the hindgut of wood-feeding higher termites PNAS 115(51) E11996-E12004. 2018.
41. Chantarasiri A. Aquatic Bacillus cereus JD0404 isolated from the muddy sediments of mangrove swamps in Thailand and characterization of its cellulolytic activity.  Egypt J Aquat Res. 2015;41(3):257-264.
42. Johnsen HR and Krause K. Cellulase activity screening using pure carboxymethylcellulose: application to soluble cellulolytic samples and to plant tissue prints. Int J Mol Sci. 2014;15(1):830-838.
43. Gupta P, Samant K and Sahu A. Isolation of cellulose-degrading bacteria and determination of their cellulolytic potential. Int j microbial. 2012.
44. Liu B, Lin JD, Song SL and Wu DL. The Microbes Cultured and Their Function in the Gut of Termites.  Bio Dis Sci. 2012.
45. Purwadari T, Ketaren PP, Sinurat AP, and Sutikno I. Identification and evaluation of fiber hydrolytic enzymes in the extract of termites (Glyptotermes montanus) for poultry feed application. Indo Journ Agri Sci. 2003;4,40-47.
46. Zhang M, Liu N, Qian C, Wang Q, Long Y, Huang Y, Zhou Z and Yan X. Phylogenetic and functional analysis of gut microbiota of a fungus-growing higher termite: Bacteroidetes from higher termites are a rich source of β-glucosidase genes.  Micro ecol. 2014;68(2):416-425.
47. Katiyar P, Srivastava SK and Tyagi VK. Measurement of cellulolytic potential of cellulase producing bacteria. Annu Res Rev Biol. 2018;1-9.
48. Veivers PC, O'Brien RW and Slaytor M. Role of bacteria in maintaining the redox potential in the hindgut of termites and preventing entry of foreign bacteria, J Insect Physiol. 1982;28(11):947-951.
49. Mattéotti C, Bauwens J, Brasseur C, Tarayre C, Thonart P, Destain J, Francis F, Haubruge E De Pauw E, Portetelle D and Vandenbol M. Identification and characterization of a new xylanase from Gram-positive bacteria isolated from termite gut (*Reticulitermes santonensis*). Protein Expr and Pur. 2012;83(2):117-127.
50. Mulyani PD, Hamid RM, Janatunaim RZ, Purwestri YA. Amylolytic ability of bacteria isolated from termite (Coptotermes sp.) gut. Indo Journ Biotechnol. 2018;23(1):14-20.
51. Sakolvaree J and Deevong P. Isolation and Characterization of Cellulase Producing Bacteria from the Gut of a Higher Termite *Termes propinquus*. 2016.
52. Tarayre C, Brognaux A, Bauwens J, Brasseur C, Mattéotti C, Millet C, Destain J, Vandenbol M, Portetelle D, De Pauw E and Eric H. Isolation of amylolytic xylanolytic and cellulolytic microorganisms extracted from the gut of the termite Reticulitermes santonensis by means of a micro-aerobic atmosphere. World J Microbiol Biotechnol. 2014;30(5):1655-1660.
53. Breznak JA and Brune A. Role of microorganisms in the digestion of lignocellulose by termites. Annu rev entomol. 1994;39:453-487.
54. Ramin M, Alimon AR, Sijam K and Abdullah N. Filter paper degradation by bacteria isolated from local termite gut Res J Microbiol. 2008;3(8):565-568.
55. Shinde VS, Shinde RM, Agrawal T and Kotasthane AS. Extracellular cellulase activity of termite gut bacteria isolated from different geographical locations of chhattisgarh (CG). India Plant Archiv. 2017;17(1):601-607.
56. Yang H, Schmitt‐Wagner D, Stingl U and Brune A. Niche heterogeneity determines bacterial community structure in the termite gut (*Reticulitermes santonensis*). Environ Microbiol. 2005;7(7):916-932.
57. Chew YM, Lye S, Salleh MM and Yahya A. 16S rRNA metagenomic analysis of the symbiotic community structures of bacteria in foregut midgut and hindgut of the wood-feeding termite. Bulbitermes sp Symbiosis. 2018;76(2):187-197.
58. Köhler T, Dietrich C, Scheffrahn RH and Brune A. High-resolution analysis of gut environment and bacterial microbiota reveals functional compartmentation of the gut in wood-feeding higher termites (*Nasutitermes spp*). AEM. 2012;78(13):4691-4701.
59. Miyata R, Noda N, Tamaki H, Kinjyo K, Aoyagi H, Uchiyama H and Tanaka H. Influence of feed components on symbiotic bacterial community structure in the gut of the wood-feeding higher termite *Nasutitermes takasagoensis*. Biosci biotechnol biochem. 2007;71(5):1244-1251.
60. Okano K, Ohkoshi N, Nishiyama A, Usagawa T and Kitagawa M. Improving the nutritive value of madake bamboo Phyllostachys bambusoides for ruminants by culturing with the white-rot fungus *Ceriporiopsis subvermispora*. Anim Feed Sci Technol. 2009;152(3-4):278-285.
61. Azizi A, Sharifi A and Fazaeli H. Effect of one produced slow-release urea component on gas production fermentation nutrient disappearance and activity of microbial enzymes using rumen liquor of sheep. Anim Sci J. 2019;32(122):279-290.
62. Adams L, Boopathy R. Isolation and characterization of enteric bacteria from the hindgut of Formosan termite. Bioresour Technol. 2005;96:1592–8.

**Table 1** Morphological and biochemical characteristics of different isolates

| Isolates | Gram Stain | Shape | Gas Production  Test | Anaerobiosis  Test | Catalase  Test | Motility  Test | H_2_S Production  Test | Indole Production  Test | Methyl Red  Test |
| --- | --- | --- | --- | --- | --- | --- | --- | --- | --- |
| TM1 | + | Rod | - | + | - | Motile | + | - | - |
| TM2 | - | Rod | + | + | - | Motile | + | - | + |
| TM3 | - | Rod | + | + | - | Motile | + | - | + |
| TM4 | - | Rod | + | + | - | Motile | + | - | + |
| TM5 | - | Rod | - | + | - | Motile | + | - | - |
| TM6 | + | Rod | - | + | - | Non motile | + | - | + |
| TM7 | + | Rod | - | + | - | Motile | + | - | - |
| TM8 | + | Rod | + | - | + | Non motile | + | - | + |
| TM9 | + | Rod | - | - | + | Motile | + | - | + |
| TM10 | + | Rod | - | + | - | Motile | + | - | + |
| TM11 | + | Rod | + | + | - | Non motile | + | - | - |
| TM12 | + | Rod | - | + | - | Motile | - | - | + |
| TM13 | + | Rod | - | + | - | Motile | + | - | + |
| TM14 | - | Rod | + | - | + | Non motile | + | - | + |
| TM15 | - | Rod | + | + | - | Non motile | + | - | + |
| TM16 | + | Cocci | + | + | - | Non motile | + | - | + |
| TM17 | + | Rod | - | + | - | Motile | + | - | + |
| TM18 | + | Cocci | + | + | - | Non motile | + | - | + |
| TM19 | + | Rod | - | + | - | Non motile | + | - | + |
| TM20 | + | Rod | - | + | - | Non motile | + | - | + |
| TM21 | - | Rod | + | + | - | Motile | + | - | + |
| TM22 | - | Rod | - | - | + | Non motile | + | - | + |
| TM23 | + | Rod | + | + | - | Non motile | + | - | + |
| TM24 | + | Rod | + | + | - | Motile | + | - | - |

(-) Non-utilizers, (+) Utilizers

**Table 2** Utilization of various carbohydrates by different isolates

| Isolates | 1 | 2 | 3 | 4 | 5 | 6 | 7 | 8 | 9 | 10 | 11 | 12 | 13 |
| --- | --- | --- | --- | --- | --- | --- | --- | --- | --- | --- | --- | --- | --- |
| TM1 | + | + | + | + | - | - | + | + | + | + | + | + | + |
| TM2 | + | + | + | + | + | + | + | + | + | + | + | + | + |
| TM3 | + | + | + | + | + | + | + | + | + | + | + | + | - |
| TM4 | + | + | + | + | + | + | + | + | + | + | + | + | - |
| TM5 | + | + | + | + | + | + | + | + | + | + | + | + | - |
| TM6 | + | - | + | + | + | + | + | + | + | + | + | + | - |
| TM7 | + | + | + | + | - | - | + | + | + | + | + | + | - |
| TM8 | + | + | + | + | + | + | + | + | + | + | + | + | - |
| TM9 | + | + | + | + | + | + | + | + | + | + | + | + | - |
| TM10 | - | + | + | + | + | - | + | + | + | + | + | + | + |
| TM11 | + | + | + | + | + | + | + | + | + | + | + | + | + |
| TM12 | + | - | + | + | + | + | + | + | + | + | + | + | + |
| TM13 | - | + | + | + | + | + | + | - | + | + | + | + | + |
| TM14 | + | + | + | + | + | + | + | - | + | + | + | + | + |
| TM15 | - | + | + | + | - | - | + | - | + | + | + | + | + |
| TM16 | + | **+** | + | + | + | + | + | + | + | + | + | + | - |
| TM17 | + | + | + | + | + | + | + | + | + | + | + | + | + |
| TM18 | - | + | - | + | + | + | + | - | + | + | + | + | - |
| TM19 | + | + | + | + | + | + | + | + | + | + | + | + | + |
| TM20 | - | + | + | + | + | - | + | - | + | + | + | + | - |
| TM21 | - | + | + | + | + | + | + | - | + | + | + | + | + |
| TM22 | + | + | + | + | + | + | + | + | + | + | + | + | - |
| TM23 | + | + | + | + | + | + | + | + | + | + | + | + | + |
| TM24 | + | + | - | + | + | + | + | - | + | + | + | + | + |

(-) Non-utilizers, (+) Utilizers

1. Galactose, 2. Mannose, 3. Sucrose, 4. Glucose, 5. Xylose, 6. Xylan, 7. Carboxy-methyl cellulose, 8. Fructose, 9. Lactose, 10. Arabinose, 11. Starch, 12. Maltose, 13. Ribose

**Table 3**	Fibrolytic enzyme activities of bacterial isolates

| Isolates | 1 | 2 | 3 | 4 | 5 | 6 |
| --- | --- | --- | --- | --- | --- | --- |
| TM1 | 5.21^abcd^ | 7.08^efghi^ | 8.58^bcdef^ | 0.009^a^ | 5.17^cdef^ | 1.72^abcd^ |
| TM2 | 5.00^ab^ | 6.53^defghi^ | 8.03^abcde^ | 0.071^d^ | 3.35^abc^ | 8.44^kl^ |
| TM3 | 5.69^abcdef^ | 5.87^bcdef^ | 8.93^cdef^ | 0.015^ab^ | 2.04^a^ | 3.11^cdefg^ |
| TM4 | 5.51^abcdef^ | 6.16 ^cdef^ | 8.45^bcdef^ | 0.026^abc^ | 4.16a^bcd^ | 2.88^bcdef^ |
| TM5 | 4.83^a^ | 5.57^bcde^ | 12.05^g^ | 0.041^abcd^ | 5.32c^defg^ | 12.00^m^ |
| TM6 | 7.60^h^ | 8.40^j^ | 8.67^bcdef^ | 0.059^cd^ | 7.34f^ghi^ | 5.55^hij^ |
| TM7 | 5.97^bcdefg^ | 7.08^efghi^ | 8.58^bcdef^ | 0.009^a^ | 5.17^cdef^ | 1.72^abcd^ |
| TM8 | 6.31^efg^ | 7.64^ghij^ | 8.35^bcdef^ | 0.072^d^ | 5.68^cdefgh^ | 3.44^defg^ |
| TM9 | 6.83^gh^ | 7.49^fghij^ | 9.64^ef^ | 0.031^abcd^ | 4.36^abcd^ | 1.11^abc^ |
| TM10 | 6.36^fg^ | 8.14^ij^ | 8.93^cdef^ | 0.055^bcd^ | 6.03^defgh^ | 0.83^ab^ |
| TM11 | 5.69^abcdef^ | 6.22^cdefg^ | 8.41^bcdef^ | 0.040^abcd^ | 2.19^ab^ | 2.88^bcdef^ |
| TM12 | 5.90^bcdefg^ | 7.69^hij^ | 7.81^abcd^ | 0.371^fg^ | 10.73^jk^ | 5.11^ghij^ |
| TM13 | 5.30^abcde^ | 5.37^abcde^ | 9.42^def^ | 0.396^gh^ | 5.02^cdef^ | 2.11^abcde^ |
| TM14 | 5.92^cdefg^ | 6.38^cdefgh^ | 8.80^bcdef^ | 0.384^fgh^ | 9.67^ij^ | 10.16^lm^ |
| TM15 | 5.37^bcdef^ | 6.11^cdef^ | 7.12^ab^ | 0.407^gh^ | 8.10^hi^ | 0.66^a^ |
| TM16 | 5.25^abcd^ | 5.12^abc^ | 8.76^bcdef^ | 0.501^k^ | 2.34^ab^ | 5.83^ij^ |
| TM17 | 6.21^defg^ | 5.53^bcde^ | 7.67^abc^ | 0.454^ij^ | 11.23^jk^ | 3.66^defgh^ |
| TM18 | 5.63^abcdef^ | 5.55^bcde^ | 7.44^abc^ | 0.541^l^ | 3.35^abc^ | 5.00f^ghij^ |
| TM19 | 5.65^abcdef^ | 4.24^a^ | 6.41^a^ | 0.465^jk^ | 3.76a^bcd^ | 3.92^efghi^ |
| TM20 | 5.17^abc^ | 4.79^ab^ | 7.52^abc^ | 0.423^hi^ | 7.85^ghi^ | 0.55^a^ |
| TM21 | 5.32^abcde^ | 5.20^abcd^ | 7.24^abc^ | 0.349^f^ | 4.69^bcde^ | 7.1^jk^ |
| TM22 | 6.28^efg^ | 6.38^cdefgh^ | 9.92^f^ | 0.124^e^ | 12.42^k^ | 8.27^kl^ |
| TM23 | 7.24^h^ | 6.18^cdef^ | 7.95^abcde^ | 0.051^abcd^ | 6.36^defgh^ | 6.77^jk^ |
| TM24 | 5.37^abcdef^ | 6.64^defgh^ | 8.40^bcdef^ | 0.073^d^ | 3.08^abc^ | 10.22^lm^ |
| SEM | 0.08398 | 0.12864 | 0.13664 | 0.0222 | 0.33731 | 0.37590 |
| pValue | 0.000 | 0.000 | 0.000 | 0.000 | 0.000 | 0.000 |

1. Endoglucanase Activity (µmol glucose/h/ml or units/ml), 2. Exoglucanase Activity (µmol glucose/h/ml or units/ml), 3. FPase Activity (µmol glucose/h/ml or units/ml), 4. β-Glucosidase Activity (µmol p-Nitrophenol/min/ml or units/ml), 5. α-Amylase Activity (µmol glucose/h/ml or units/ml), 6. Xylanase Activity (µmol xylose/h/ml or units/ml), ^a-m^ means bearing superscript differs significantly in a column (P < 0.05).

**Table 4** Filter paper degradation potential of different isolates

| SN | 24h | 48h | 72h | 96h | 5D | 6D | 7D | 8D |
| --- | --- | --- | --- | --- | --- | --- | --- | --- |
| TM1 | + | ++ | ++ | +++ | ++++ | +++++ | ++++++ | CD |
| TM2 | + | + | ++ | +++ | ++++ | +++++ | ++++++ | CD |
| TM3 | + | ++ | ++ | +++ | ++++ | +++++ | ++++++ | CD |
| TM4 | + | ++ | ++ | +++ | ++++ | +++++ | ++++++ | CD |
| TM5 | ++ | +++ | ++++ | +++++ | +++++ | ++++++ | CD |  |
| TM6 | + | ++ | +++ | ++++ | +++++ | +++++ | ++++++ | CD |
| TM7 | + | ++ | ++ | +++ | ++++ | +++++ | ++++++ | CD |
| TM8 | + | + | ++ | +++ | ++++ | +++++ | ++++++ | CD |
| TM9 | + | ++ | +++ | ++++ | +++++ | ++++++ | CD |  |
| TM10 | + | ++ | +++ | ++++ | +++++ | +++++ | ++++++ | CD |
| TM11 | + | ++ | ++ | +++ | ++++ | +++++ | ++++++ | CD |
| TM12 | + | + | ++ | +++ | ++++ | +++++ | ++++++ | CD |
| TM13 | + | ++ | +++ | ++++ | +++++ | ++++++ | CD |  |
| TM14 | + | + | ++ | +++ | ++++ | +++++ | ++++++ | CD |
| TM15 | + | + | ++ | +++ | ++++ | +++++ | ++++++ | CD |
| TM16 | + | + | ++ | +++ | ++++ | +++++ | ++++++ | CD |
| TM17 | + | ++ | ++ | +++ | ++++ | +++++ | ++++++ | CD |
| TM18 | + | ++ | ++ | +++ | ++++ | +++++ | ++++++ | CD |
| TM19 | + | + | ++ | +++ | ++++ | +++++ | ++++++ | CD |
| TM20 | + | + | ++ | +++ | ++++ | +++++ | ++++++ | CD |
| TM21 | + | + | +++ | +++ | ++++ | +++++ | ++++++ | CD |
| TM22 | + | ++ | ++ | +++ | ++++ | +++++ | ++++++ | CD |
| TM23 | + | + | ++ | +++ | ++++ | +++++ | ++++++ | CD |
| TM24 | + | ++ | ++ | +++ | ++++ | +++++ | ++++++ | CD |

*CD= Complete Degradation, h=Hours, D= Days

**Table 5** 16S rDNA sequencing based identification

| **Isolates** | **Nearest Valid Taxon** | **% similarity** | **Sequence base pairs** | **Phylum** | **Family** | **Gene Bank Accession** |
| --- | --- | --- | --- | --- | --- | --- |
| TM1 | *Bacillus megaterium strain IAM 13418* | 99 | 1473 | Firmicutes | Bacillaceae | MT356127 |
| TM2 | *Citrobacter freundii strain JCM 1657* | 98 | 1417 | Proteobacteria | Enterobacteriaceae | MT356128 |
| TM3 | *Citrobacter farmeri strain CDC 2991-81* | 99 | 1472 | Proteobacteria | Enterobacteriaceae | MT356129 |
| TM4 | *Citrobacter freundii strain JCM 1657* | 98 | 1427 | Proteobacteria | Enterobacteriaceae | MT356130 |
| TM5 | *Bacillus flexus strain IFO15715* | 99 | 1530 | Firmicutes | Bacillaceae | MT356131 |
| TM6 | *Pilibacter termitis strain TI-1* | 98 | 1413 | Firmicutes | Enterococcaceae | MT356132 |
| TM7 | *Bacillus megaterium strain IAM* | 99 | 1482 | Firmicutes | Bacillaceae | MT356133 |
| TM8 | *Bacillus licheniformis strainATCC14580* | 99 | 1485 | Firmicutes | Bacillaceae | MT356134 |
| TM9 | *Bacillus cereus strain ATCC 14579* | 98 | 1524 | Firmicutes | Bacillaceae | MT356135 |
| TM10 | *Clostridium termitidis* | 99 | 1446 | Firmicutes | Hungateiclostridiaceae | MT356136 |
| TM11 | *Clostridium saccharobutylicum strain P262* | 100 | 1353 | Firmicutes | Clostridiaceae | MT356137 |
| TM12 | *Clostridium indolis strain 7* | 97 | 1414 | Firmicutes | Lachnospiraceae | MT356138 |
| TM13 | *Clostridium termitidis* | 98 | 1446 | Firmicutes | Hungateiclostridiaceae | MT356139 |
| TM14 | *Bacillus oleronius strain DSM 9356* | 98 | 1477 | Firmicutes | Bacillaceae | MT356140 |
| TM15 | *Clostridium cellulovorans 743B* | 96 | 1447 | Firmicutes | Clostridiaceae | MT356141 |
| TM16 | *Lactococcus nasutitermitis strain M19* | 97 | 1446 | Firmicutes | Streptococaceae | MT356142 |
| TM17 | *Clostridium sporogenes strain JCM 1416* | 90 | 1535 | Firmicutes | Clostridiaceae | MT356143 |
| TM18 | *Lactococcus nasutitermitis strain M19* | 99 | 1440 | Firmicutes | Streptococaceae | MT356144 |
| TM19 | *Bacillus circulans strain ATCC 4513* | 98 | 1482 | Firmicutes | Bacillaceae | MT356145 |
| TM20 | *Bacillus circulans strain ATCC 4513* | 98 | 1482 | Firmicutes | Bacillaceae | MT356146 |
| TM21 | *Ruminiclostridium papyrosolvens DSM 2782* | 98 | 1382 | Firmicutes | Hungateiclostridiaceae | MT356147 |
| TM22 | *Brevibacillus brevis strain DSM 30* | 96 | 1447 | Firmicutes | Paenibacillaceae | MT356148 |
| TM23 | *Clostridium puniceum strain BL 70/20* | 96 | 1481 | Firmicutes | Clostridiaceae | MT356149 |
| TM24 | *Clostridium sporogenes strain JCM 1416* | 99 | 1489 | Firmicutes | Clostridiaceae | MT356150 |

**Table 6 Fermentation attributes total short chain fatty acid production**

| S.no. | Acetate(C2) mM | Propionate (C3) mM | Iso-Butyrate (C4i) mM | Butyrate (C4) mM | Iso-Valerate (C5i) mM | Valerate (C5) mM | Total SCFAs mM | BcFA | NGR | C3:(C2+C4) | C2:C3 |
| --- | --- | --- | --- | --- | --- | --- | --- | --- | --- | --- | --- |
| TM1 | 56.74^i^ | 14.26^f^ | 1.71^bcd^ | 7.34^g^ | 0.96^def^ | 1.18^g^ | 82.19^hij^ | 3.85^cde^ | 4.16^ab^ | 0.22^defg^ | 3.98^abc^ |
| TM2 | 70.72^k^ | 14.09^ef^ | 2.83^f^ | 6.93^defg^ | 1.15^fg^ | 1.33^g^ | 97.05^k^ | 5.32^fg^ | 4.64^bc^ | 0.18^b^ | 5.03^cd^ |
| TM3 | 60.88^j^ | 14.10^ef^ | 1.96^cde^ | 7.30^fg^ | 0.92^def^ | 0.98^f^ | 86.15^j^ | 3.87^cde^ | 4.42^abc^ | 0.21^cd^ | 4.32^abc^ |
| TM4 | 56.96^i^ | 13.96^ef^ | 1.86^bcd^ | 7.16^fg^ | 1.01^efg^ | 1.27^g^ | 82.23^hij^ | 4.14^e^ | 4.18^ab^ | 0.22^def^ | 4.09^abc^ |
| TM5 | 61.35^j^ | 6.93^a^ | 2.82^f^ | 3.08^ab^ | 0.96^def^ | 0.39^b^ | 75.54^efg^ | 4.17^e^ | 6.55^e^ | 0.11^a^ | 8.97^f^ |
| TM6 | 83.59^l^ | 17.87^g^ | 2.77^f^ | 10.34^h^ | 1.56^h^ | 1.61^h^ | 117.73^l^ | 5.93^g^ | 4.63^bc^ | 0.19^bc^ | 4.68^bcd^ |
| TM7 | 44.82^b^ | 10.61^b^ | 1.37^bcd^ | 5.92^def^ | 0.73^bcd^ | 0.74^cde^ | 64.19^b^ | 2.84^bc^ | 4.43^abc^ | 0.21^cd^ | 4.23^abc^ |
| TM8 | 51.47^efg^ | 13.43^def^ | 1.54^bcd^ | 7.15^fg^ | 0.93^def^ | 0.96^f^ | 75.50^efg^ | 3.44^bcde^ | 4.11^ab^ | 0.23^defh^ | 3.84^ab^ |
| TM9 | 49.26^de^ | 13.43^def^ | 1.42^bcd^ | 7.03^defg^ | 0.80^bcde^ | 0.97^f^ | 72.91^cdef^ | 3.19^bcde^ | 4.01^ab^ | 0.24^efghi^ | 3.67^ab^ |
| TM10 | 44.82^b^ | 10.61^b^ | 1.37^bcd^ | 5.92^def^ | 0.73^bcd^ | 0.74^cde^ | 64.19^b^ | 2.84^bc^ | 4.43^abc^ | 0.21^cd^ | 4.23^abc^ |
| TM11 | 55.17^hi^ | 6.92^a^ | 2.60^e^ | 3.67^bc^ | 1.23^g^ | 0.56^c^ | 70.15^bcde^ | 4.39^ef^ | 5.96^de^ | 0.12^a^ | 8.05^e^ |
| TM12 | 63.17^j^ | 11.29^bc^ | 2.05^de^ | 4.46^c^ | 1.13^fg^ | 0.90^ef^ | 83.00^ij^ | 4.08^de^ | 4.97^c^ | 0.17^b^ | 5.61^d^ |
| TM13 | 51.44^efg^ | 13.14^cdef^ | 1.53^bcd^ | 6.74^defg^ | 0.82^bcde^ | 0.97^f^ | 74.65^defg^ | 3.32^bcde^ | 4.15^ab^ | 0.23^defg^ | 3.92^ab^ |
| TM14 | 44.26^b^ | 12.10^bcde^ | 1.37^bcd^ | 6.14^defg^ | 0.71^bcd^ | 0.79^def^ | 65.39^b^ | 2.88^bcd^ | 3.98^ab^ | 0.24^fghi^ | 3.67^ab^ |
| TM15 | 50.37^ef^ | 14.85^f^ | 1.44^bcd^ | 7.41^g^ | 0.90^cdef^ | 0.98^f^ | 75.96^efgh^ | 3.32^bcde^ | 3.78^a^ | 0.26^i^ | 3.40^a^ |
| TM16 | 55.47^hi^ | 14.10^ef^ | 1.54^bcd^ | 7.15^fg^ | 0.93^def^ | 0.96^f^ | 80.16^ghij^ | 3.44^bcde^ | 4.20^abc^ | 0.23^defg^ | 3.96^abc^ |
| TM17 | 45.67^bc^ | 13.23^cdef^ | 1.30^abcd^ | 6.55^defg^ | 0.71^bcd^ | 0.75^cde^ | 68.20^bcd^ | 2.76^bc^ | 3.86^ab^ | 0.25^hi^ | 3.46^a^ |
| TM18 | 48.24^cde^ | 11.22^b^ | 1.17^abc^ | 5.74^de^ | 0.65^bc^ | 0.62^cd^ | 67.63^bc^ | 2.44^b^ | 4.55^abc^ | 0.21^cd^ | 4.30^abc^ |
| TM19 | 49.25^de^ | 13.89^ef^ | 1.20^abc^ | 6.65^defg^ | 0.71^bcd^ | 0.78^def^ | 72.48^cdef^ | 2.70^bc^ | 3.93^ab^ | 0.25^ghi^ | 3.55^a^ |
| TM20 | 49.96^def^ | 13.34^def^ | 1.10^ab^ | 6.91^defg^ | 0.73^bcd^ | 0.81^def^ | 72.85^cdef^ | 2.64^bc^ | 4.16^ab^ | 0.23^deghi^ | 3.75^ab^ |
| TM21 | 50.66^efg^ | 14.02^ef^ | 1.18^abc^ | 6.91^defg^ | 0.78^bcde^ | 0.76^cde^ | 74.30^defg^ | 2.71^bc^ | 4.02^ab^ | 0.24^fghi^ | 3.62^ab^ |
| TM22 | 46.86^bcd^ | 11.75^bcd^ | 1.20^abc^ | 5.68^d^ | 0.63^b^ | 0.65^cd^ | 66.77^bc^ | 2.48^b^ | 4.27^abc^ | 0.22^defg^ | 3.99^abc^ |
| TM23 | 53.80^ghi^ | 14.54^f^ | 1.21^abc^ | 7.09^efg^ | 0.75^bcde^ | 0.79^def^ | 78.19^fghi^ | 2.76^bc^ | 4.09^ab^ | 0.24^efghi^ | 3.70^ab^ |
| TM24 | 53.12^fgh^ | 13.22^cdef^ | 1.29^abcd^ | 6.93^defg^ | 0.70^bcd^ | 0.81^def^ | 76.07^efgh^ | 2.79b^c^ | 4.36^abc^ | 0.22^def^ | 4.02^abc^ |
| Control | 24.63^a^ | 5.66^a^ | 0.58^a^ | 2.04^a^ | 0.15^a^ | 0.19^a^ | 33.26^a^ | 0.92^a^ | 4.50^abc^ | 0.21^cde^ | 4.35^abc^ |
| SEM | 1.21 | 0.318 | 0.071 | 0.05 | 0.03 | 0.03 | 1.64 | 0.12 | 0.07 | 0.03 | 0.155 |

Branch − chain fatty acids (BcFA) = valerate + iso − valerate + iso − butyrate (C5 + C5i + C4i).NGR ­Non - glucogenic: Glucogenicratio‑ = [(Acetate + 2 × Butyrate + BcFA ÷ ­Propionate + BcFA‑ ] or [ ­C2 + 2C4 + C5 + C5i + C4i‑ ÷ ­C3 + C5 + C5i + C4i‑ ].a,b,c Means bearing different superscript differ significantly (p < .05). SCFA, short-chain fatty acids; and SEM, standard error of mean.

**Figure 1.**16s rDNA amplification of isolates (TM1-TM24) of size ̴1500 bp and L is 1Kb Ladder

|  |  |
| --- | --- |
| Firmicutes | 87.5 |
| Proteobacteria | 12.5 |

**Figure 2.** Main phyla abundance of fibrolytic bacteria isolated from gut of wood eating lower termite (*Coptotermes heimi*)

|  |  |
| --- | --- |
| Bacillaceae | 33.3333 |
| Enterobacteriaceae | 12.5 |
| Paenibacillaceae | 4.16667 |
| Lachnospiraceae | 4.16667 |
| Streptococaceae | 4.16667 |
| Enterococcaceae | 4.16667 |
| Clostridiaceae | 37.5 |

**Figure3.** Distribution of fibrolytic bacteria identified in the gut of wood eating lower termite (*Coptotermes heimi*) at family level

**Figure 4** Phylogentic tree based on the 16S rRNA partial sequences of the isolates. Scale bar=1 inferred nucleotide substitution per 50 nucleotides.

Digestibility (%)

| Isolates | IVDMD (%) |
| --- | --- |
| C | 33.0327 |
| TM1 | 37.1327 |
| TM2 | 43.0452 |
| TM3 | 39.6452 |
| TM4 | 40.5577 |
| TM5 | 39.2577 |
| TM6 | 44.9327 |
| TM7 | 38.5952 |
| TM8 | 41.6077 |
| TM9 | 41.1827 |
| TM10 | 36.1327 |
| TM11 | 36.9702 |
| TM12 | 42.4106 |
| TM13 | 41.6981 |
| TM14 | 38.6231 |
| TM15 | 39.1106 |
| TM16 | 43.1606 |
| TM17 | 40.5481 |
| TM18 | 38.0106 |
| TM19 | 40.1981 |
| TM20 | 41.1731 |
| TM21 | 41.1356 |
| TM22 | 39.4356 |
| TM23 | 38.5481 |
| TM24 | 41.1606 |

**Figure5** Effect of inclusion of bacterial isolates on *in vitro* dry matter (IVDMD) digestibility at 48h post incubation.
