# Supplementary material for: Lower termite (Coptotermes heimi) gut fibrolytic bacterial consortium: Isolation, phylogenetic characterization, fibre degradation potential and in vitro digestibility
Source: PLoS One. 2025 Mar 10;20(3):e0318090. doi: 10.1371/journal.pone.0318090 (PMC11892857; doi:10.1371/journal.pone.0318090)
Supplement: Attachment — Submitted filename: Response.docx [file pone.0318090.s007.docx]

**RESPONSE TO COMMENTS**

**Journal Name:** PLOS One

**Manuscript No:** PONE-D-24-05915R1

**Manuscript title**: "Lower termite (*Coptotermes heimi*) gut fibrolytic bacterial consortium: Isolation, phylogenetic characterization, fibre degradation potential and in vitro digestibility"

**Dear Editor**,

Thank you and the reviewers for the useful suggestions on the structure of our manuscript. The suggestions were helpful in improving our manuscript and we have addressed them in the revised version.

Here we are also providing the detailed responses to the comments

| **Comments** | **Response to comments** |
| --- | --- |
| **Reviewer #1** | |
| Line 15: Please provide the full name of the author. | Needful corrections made |
| Line 40: Delete comma | Corrected as suggested |
| Line 172: Double full stops | Corrected as suggested |
| Reviewer #2 | |
| Line 23: The term ‘isolates’ is repeated after (TM1 to TM24). Please correct it. | Needful corrections made |
| Line 24: You mentioned that all isolates were obligatory anaerobes except four (TM8, TM9, TM14 and TM22). Kindly mention whether these four isolates are facultative anaerobes or aerobes. | Sentence modified as suggested |
| Line 48: There is no spacing between fiber. Please correct it. | Needful corrections made |
| Line 55: Double full stop after abundance. Please correct it. | Needful corrections made |
| Line 74: Whether it is Pfenning trace elements or Pfennig trace elements. Kindly confirm. | Needful corrections made |
| Line 82: -80°C (minus 80°C) should be written combinedly. | Corrected as suggested |
| Line 153: There is no spacing between g for. Please correct it. | Corrected as suggested |
| Line 173: Double full stop. Please correct it. | Corrected as suggested |
| Line 183: There is no spacing between (12.05 U/ml) and. Kindly correct it. | Needful corrections made |
| Line 186: There is no spacing between TM5and. Kindly correct it. | Needful corrections made |
| Line 283: Double full stop. Please correct. | Corrected as suggested |
| Line 286: Whether it is termites fed xylan or termites fed on xylan. Kindly correct it. | Corrected as suggested |
| Kindly recheck the manuscript for spacing and punctuation mistakes. | Agreed and complied |

Regards

Artabandhu Sahoo
