# Supplementary material for: Lower termite (Coptotermes heimi) gut fibrolytic bacterial consortium: Isolation, phylogenetic characterization, fibre degradation potential and in vitro digestibility
Source: PLoS One. 2025 Mar 10;20(3):e0318090. doi: 10.1371/journal.pone.0318090 (PMC11892857; doi:10.1371/journal.pone.0318090)
Supplement: Attachment — Submitted filename: Response_auresp_3.docx [file pone.0318090.s008.docx]

**RESPONSE TO COMMENTS**

**Journal Name:** PLOS One

**Manuscript No:** PONE-D-24-05915R2

**Manuscript title**: "Lower termite (*Coptotermes heimi*) gut fibrolytic bacterial consortium: Isolation, phylogenetic characterization, fibre degradation potential and *in vitro* digestibility"

**Dear Editor**,

Thank you and the reviewers for the useful suggestions on the structure of our manuscript. The suggestions were helpful in improving our manuscript and we have addressed them in the revised version.

Herewith we are also providing the detailed responses to the comments

| **Comments** | **Response to comments** |
| --- | --- |
| **Reviewer** | |
| Could author explain in further detail the rationale behind using particularly *Coptotermes heimi* for this study. | Wood-eating termites possess remarkable potential in various biotechnological applications due to their ability to efficiently break down recalcitrant biomass. *Coptotermes heimi* are the most predominant lower termites the in semi-arid regions of India and there exists similarity between the environments in the rumen and termites' gut. This unique capability makes them valuable source of fiber-degrading enzymes which could be utilized to enhance fiber digestion in ruminants, improving feed efficiency and overall productivity in livestock systems. |
| Line 157-158, authors should provide the concentration of samples used for analysis  with GC | Needful corrections made as suggested |
| In Table 3, based on trends and value of fibrolytic enzyme activites observed, could  author comment on finding some interesting correlation between activity and  isolates. | Agreed and added in the discussion |
| In Table 4, could author comment on providing some quantitative/numerical data for  filter paper degradation potential. Also, please provide remarks on significance of “+”  in context of value | This is a qualitative test hence, no numerical data is available. Although, the significance of ‘+’ is added as a footnote below the Table. |

**Regards**

Artabandhu Sahoo
